# Deposition of lignin in four species of *Saccharum*

**DOI:** 10.1038/s41598-019-42350-3

**Published:** 2019-04-10

**Authors:** Juan Pablo Portilla Llerena, Raquel Figueiredo, Michael dos Santos Brito, Eduardo Kiyota, Juliana Lischka Sampaio Mayer, Pedro Araujo, Flavia Camila Schimpl, Murali Dama, Markus Pauly, Paulo Mazzafera

**Affiliations:** 1 Departamento de Biologia Vegetal, Instituto de Biologia, CP 6109, Universidade Estadual de Campinas, Campinas, SP 13083-970 Brazil; 2 Instituto de Ciência e Tecnologia, Universidade Federal de São Paulo, Campus São José dos Campos, São José dos Campos, SP 12231-280 Brazil; 3 Departamento de Genética Evolução e Bioagentes, Instituto de Biologia, CP 6109, Universidade Estadual de Campinas, Campinas, SP 13083-970 Brazil; 4 Heinrich Heine University, Institute for Plant Cell Biology and Biotechnology, D-40225 Düsseldorf, Germany; 5 Departamento de Produção Vegetal, Escola Superior de Agricultura Luiz de Queiroz, Universidade de São Paulo, Piracicaba, SP Brazil

## Abstract

We used primers designed on conserved gene regions of several species to isolate the most expressed genes of the lignin pathway in four *Saccharum* species. *S. officinarum* and *S. barberi* have more sucrose in the culms than *S. spontaneum* and *S. robustum*, but less polysaccharides and lignin in the cell wall. *S. spontaneum*, and *S. robustum* had the lowest S/G ratio and a lower rate of saccharification in mature internodes. Surprisingly, except for CAD, 4CL, and CCoAOMT for which we found three, two, and two genes, respectively, only one gene was found for the other enzymes and their sequences were highly similar among the species. *S. spontaneum* had the highest expression for most genes. *CCR* and *CCoAOMT B* presented the highest expression; *4CL* and *F5H* showed increased expression in mature tissues; *C3H* and *CCR* had higher expression in *S. spontaneum*, and one of the CADs isolated (*CAD* B) had higher expression in *S*. *officinarum*. The similarity among the most expressed genes isolated from these species was unexpected and indicated that lignin biosynthesis is conserved in *Saccharum* including commercial varieties Thus the lignin biosynthesis control in sugarcane may be only fully understood with the knowledge of the promotor region of each gene.

## Introduction

Bioethanol can be produced from starch and sucrose (first-generation ethanol – 1GE) but also from lignocellulosic biomass (second-generation ethanol – 2GE). For the production of 2GE the sugars used in the fermentation are from the depolymerization of the carbohydrates present in the cell wall, cellulose and hemicellulose^1,2^. Depending on the plant species, tissue wall material can represent between 40% and 80% of the plant biomass^3,4^. Grasses with C4 metabolism, especially those belonging to the subfamily Panicoideae, such as sugarcane (*Saccharum* spp.), sorghum (*Sorghum bicolor*), species of *Miscanthus*, and *Panicum virgatum*, represent plants with the greatest potential for 2GE production due to their large capacity for carbon fixation and biomass accumulation^5^.

Lignocellulosic biomass used in 2GE production is composed of cellulose, hemicellulose, and lignin, which are arranged in a chemically ordered manner in the wall. Cellulose is organized into crystalline microfibrils that are embedded in a matrix of hemicellulose, which is covalently linked to the complex structure of lignin. In 2GE production the chemical bonds between wall polymers must be broken to release sugars for downstream fermentation processes. Usually, a chemical pretreatment is needed to allow the access of enzymes to the wall polysaccharides^6^. One of the main difficulties in accessibility to the polysaccharides is the presence of lignin, which is highly resistant to degradation due to a diversity of low reactivity linkages, making this phenolic the main polymer responsible for the cell wall recalcitrance^7–9^. In addition, pretreatments can release lignin residues that can inhibit the fermentation process.

Lignin is a complex heteropolymer formed by oxidative combinatorial coupling of three alcohols that are synthesized in the cytoplasm of plant cells: p-coumaryl, coniferyl, and sinapyl alcohol. These alcohols differ in their degree of methoxylation^10^ and are transported from the cytoplasm to the apoplast, where they are oxidized by peroxidases and/or laccases into radicals, that are then incorporated by random radical reactions into the preformed polymer^11^. After the incorporation, the monolignol residues are called *p*-hydroxyphenyl (H), guaiacyl (G), and syringyl (S), respectively, and their proportion in the lignin structure varies significantly between the type of plant cells, tissues, and species^12,13^. Lignin present in gymnosperms consists of G units and small amounts of H units, whereas in angiosperms they are composed of G units, S units, and only trace amounts of H units. In monocotyledons, both S units and G units are presented at similar levels and the amount of H units is higher than in dicotyledons^14^.

The S/G ratio and the inter-monomeric linkages in the lignin polymer are important characteristics to predict the degree and nature of the condensation of the polymer and, consequently, about plant biomass recalcitrance^15^. In addition, the complexity of the lignin structure and its recalcitrance can be affected by other phenylpropanoids that can be incorporated into the polymer structure to different levels^16^. For example, a recent structural characterization of cell walls of several monocotyledons showed that the flavonoid tricin is part of native lignin^17,18^, and this monomer may act in the formation of a nucleation site for the beginning of lignin biosynthesis^18–20^.

Several species of plants have been genetically modified to change the content and composition of lignin, and the degree of modification depends on the responsible gene and on the position of the encoded enzyme in the biosynthetic pathway^21,22^. In general, changes in the expressions of *C3H*, *HCT*, or *4CL* lead to quantitative changes in the levels of lignin, while the regulation of *F5H* and *COMT* leads to changes in the S/G ratio and, consequently, in the type of lignin^7,23,24^. The recent identification of another lignin biosynthesis enzyme, Caffeoyl Shikimate Esterase (CSE), adds another step in this metabolic pathway that can be manipulated^25,26^. Indeed, transgenic poplar plants silenced for CSE showed reduced lignin content, altered S and G composition, and increased saccharification yields^27^.

Many of the studies on the biosynthetic pathway of lignin monomers were conducted in some dicotyledons (e.g. *Alfalfa* and *Populus*) and model plants such as *Arabidopsis thaliana* and *Nicotiana tabacum*^28^, in which a high degree of conservation was observed. The information obtained with these plants has been applied in studies of monocotyledons used for 2GE production^28–30^, but studies with monocotyledons are still proportionally smaller in number. The study of lignin biosynthesis in sugarcane has been conducted recently in a systematic manner^13,31–36^ and transgenic plants of sugarcane silenced for *COMT* and *CAD*^37–39^ were produced.

The genus *Saccharum* comprises more than 10 species^40^ and the term sugarcane is generally used to define complex hybrids originated from the species *S. officinarum* and *S. spontaneum*, which appear to have contributed with 90% and 10%, respectively, to its genotype^41^. Sugarcane is a C4 grass, which is highly efficient in the production of photoassimilates and biomass accumulation^42^, in addition to storing up to 18% of sucrose (wet basis) in its culms^43^. The sucrose-rich syrup obtained by crushing the culms is used in the alcoholic fermentation and production of 1GE^2,44^. The residual biomass called “bagasse” – composed primarily of cellulose (39%), hemicellulose (25%), and lignin (23%) – has huge potential for 2GE production^45–47^. However, the use of sugarcane bagasse to produce 2GE has several technical hurdles, among them the recalcitrance of the lignocellulosic material mainly due to the presence of lignin, which drastically decreases the efficiency of saccharification yield for downstream fermentation^33^.

A new type of cane, called energy cane, with lower accumulation of sucrose in the stem and richer in fiber has been considered for 2GE production^48^. The term energy cane has been used generically for the species *S. spontaneum* as well as for its hybrids with commercial varieties of sugarcane. In addition to its application in biofuel production (first and second generation ethanol) energy cane can be burned to generate electricity^42^ because of its high lignin content and the greater heating value of this polymer^49^.

To date, a systematic study related to lignin biosynthesis and cell wall biochemistry has only been conducted on *S. officinarum*, but not on any other *Saccharum* species^33^. Some species of the genus have different sucrose and fiber contents, such as *S. spontaneum*, *S. officinarum*, *S. robustum*, and *S. barberi*. *S. officinarum* and *S. spontaneum* differ in sucrose and fiber content whereby the first accumulates more sucrose but has a lower fiber content. *S. officinarum* is the only species within the genus *Saccharum* whose chromosome number is not variable between individuals^50^ and it is believed that it originates from *S. robustum*. On the other hand, *S. spontaneum* is a complex, highly polymorphic species, and the most primitive of the species of the genus *Saccharum*. The high genetic variability of this species has been used in genetic breeding programs seeking to develop commercial varieties with potential for biomass production^51^. Abundant molecular evidence indicates that *S. spontaneum* is genetically very different compared to the other species of *Saccharum*^52,53^. Similarly to *S. spontaneum* plants of *S. robustum* have culms that are rich in fiber and poor in sucrose, and although the plants are vigorous, they are susceptible to abiotic and biotic stresses^54^. Although *S. robustum* has potential to be used in breeding programs because its vigor, its use has been restricted to Hawaii^51^. Apparently, the species *S. barberi* originated from the natural hybridization of *S. officinarum* with *S. spontaneum*^55^. This species has been cultivated and has moderate content of sucrose, displaying resistance to stresses and high content of fibers in relation to *S. officinarum*. Currently, there is little interest in using *S. barberi* in breeding programs, mainly due to the difficulty of flowering and flower sterility.

Because of differing fiber content and the potential for E2G production of these species, this study aims at investigating the cell wall components, the content and type of lignin, as well as to determine and evaluate the relative expression of the genes related to lignin biosynthesis in *S. spontaneum, S. officinarum, S. robustum*, and *S. barberi*. Such information may help not only in a better understanding of the accumulation of lignin within the genus *Saccharum* but also provide useful information for the adoption of these species for 2GE production.

## Results

### Cell wall polysaccharides

**Figure 1:**
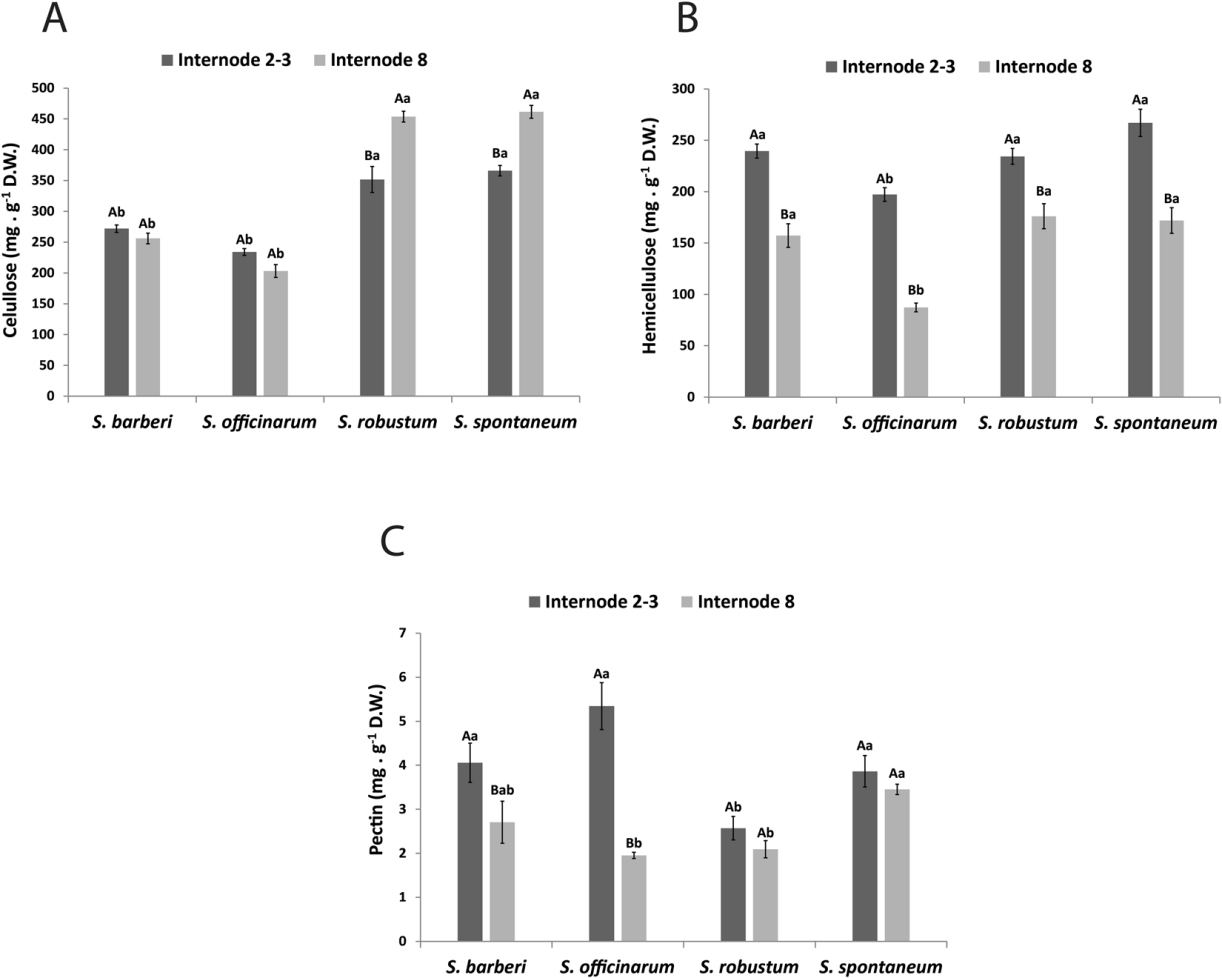
Content of (**A**) cellulose (**B**) hemicellulose and (**C**) pectin in internodes of *Saccharum* species. Different capital letters denote significant differences (p < 0.05) between internodes of different stages of development within the same species. Different lowercase letters indicate differences (p < 0.05) between internodes of the same stage of development of the different species. The averages were compared by Tukey’s test. Vertical bars indicate the standard error of the means of five replicates.

Irrespective of culm age, the cellulose content was higher in *S. spontaneum* and *S. robustum* than in *S. officinarum* and *S. barberi* (Fig. 1A). In the first two species, the highest content was observed in culms of internode 8. Hemicellulose content was always higher in internodes 2 + 3 (Fig. 1B) and the species with the lowest content was *S. officinarum*. The other species showed similar values for the culms of different ages. Pectin content (Fig. 1C) was higher in the younger internode of *S. barberi* and *S. officinarum* compared with the mature internode and similar between the internodes in the other two species. The highest pectin content was found in internodes 2 + 3 of *S. officinarum* and the highest content in internode 8 was found in *S. spontaneum*.

### Non-structural carbohydrates and starch

**Figure 2:**
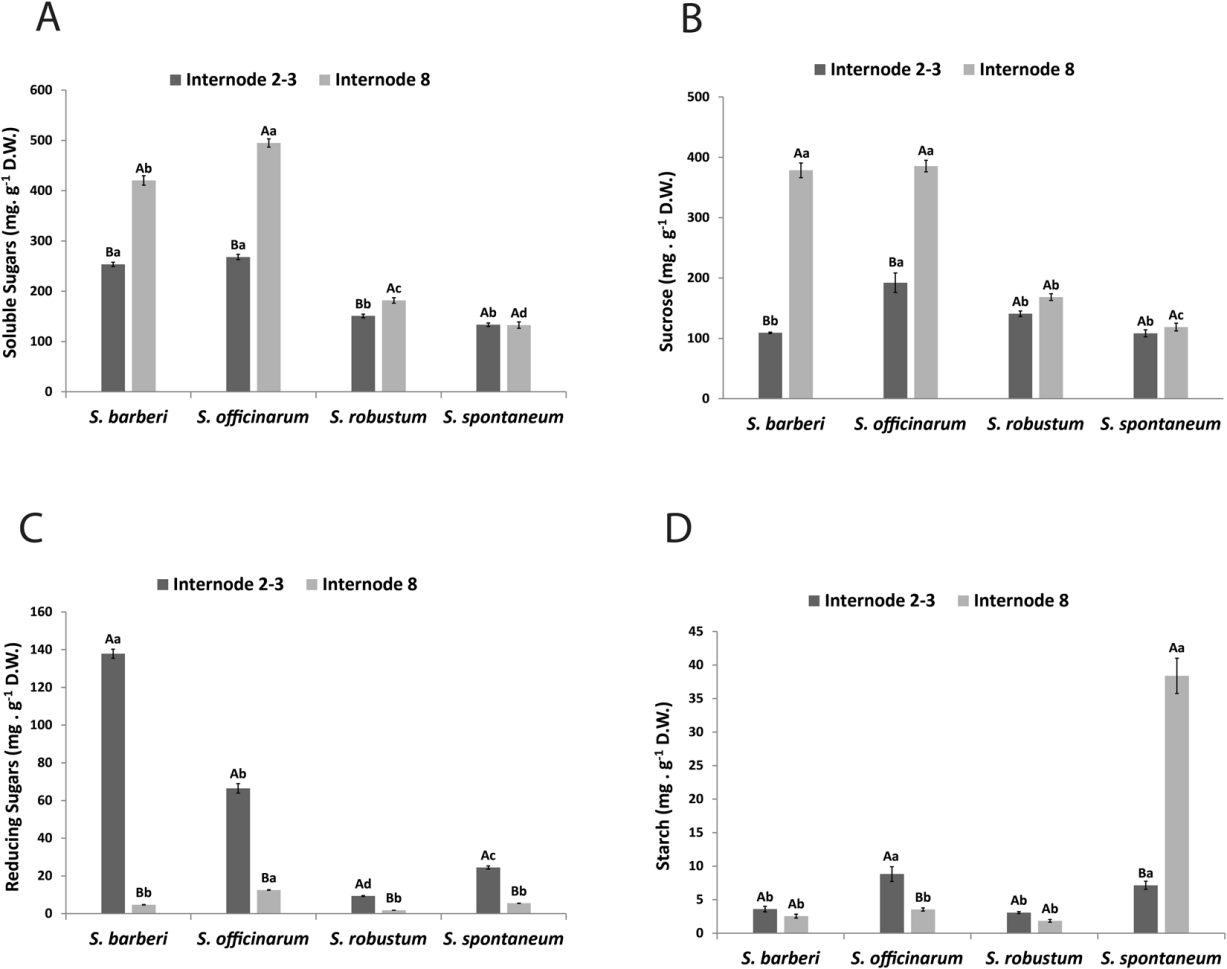
Content of (**A**) total soluble sugars, (**B**) sucrose, (**C**) reducing sugars and (**D**) starch in internodes of *Saccharum* species. Different capital letters denote significant differences (p < 0.05) between internodes of different stages of development within the same species. Different lowercase letters indicate significant differences (p < 0.05) between internodes of the same stage of development of the different species. The means were compared by the Tukey test. Vertical bars indicate the standard error of the means of five replicates.

The highest contents of total sugars (Fig. 2A) and sucrose (Fig. 2B) were found in mature culms of *S. officinarum* and *S. barberi*. These two species were also those that accumulated more reducing sugars in young culms (Fig. 2C). While sucrose contents were similar in new and mature culms of *S. robustum* and *S. spontaneum*, reducing sugar contents in these species were higher in new culms. Starch content in S. *spontaneum* was more than eight fold higher than in the other species in mature culms (Fig. 2D). Comparatively, new culms of *S. officinarum* accumulated more starch than mature culms.

### Total phenols

**Figure 3:**
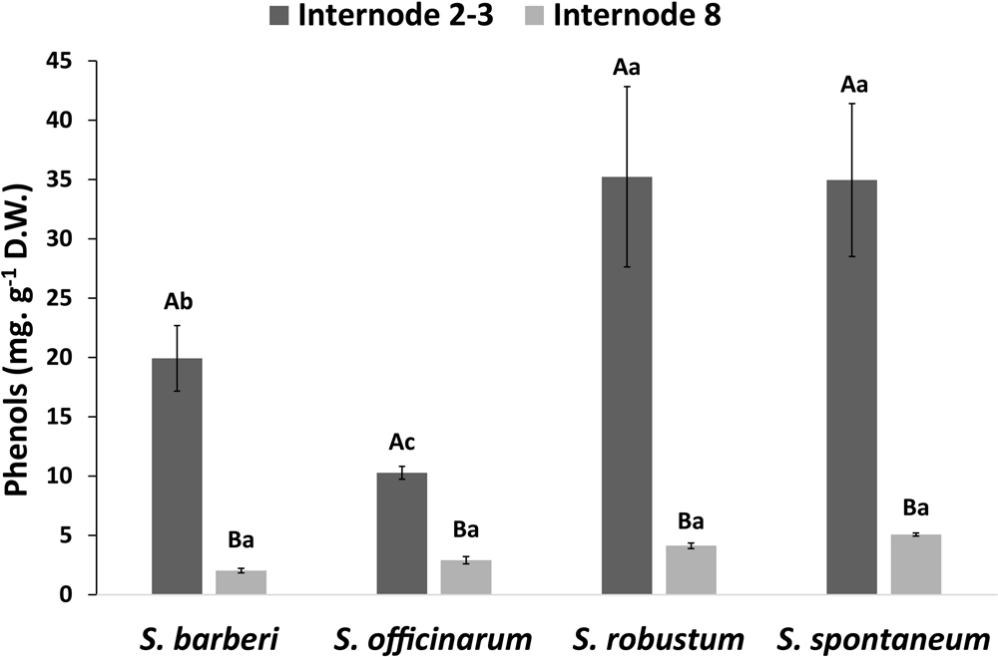
Total phenol content in internodes of *Saccharum* species. Different capital letters denote significant differences (p < 0.05) between internodes of different stages of development within the same species. Different lowercase letters indicate significant differences (p < 0.05) between internodes of the same stage of development of the different species. The means were compared by the Tukey test. Vertical bars indicate the standard error of the means of five replicates.

The highest phenol contents were found in newer internodes, and the highest values were those of the species *S. robustum* and *S. spontaneum* (Fig. 3). *S. officinarum* presented the lowest phenol content in new internodes. The four species did not differ as to the content in mature internodes.

### Soluble and insoluble lignin

**Figure 4:**
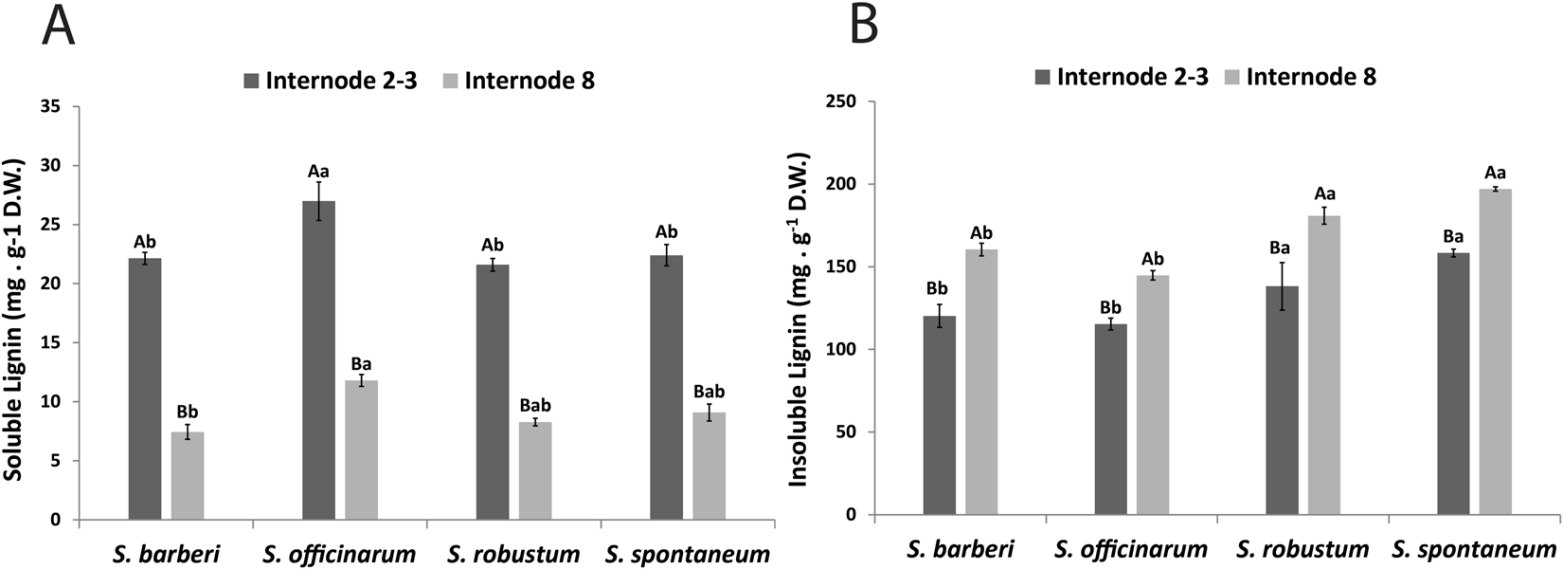
Content of (**A**) soluble lignin and (**B**) insoluble in internodes of *Saccharum* species. Different capital letters denote significant differences (p < 0.05) between internodes of different stages of development within the same species. Different lowercase letters indicate significant differences (p < 0.05) between internodes of the same stage of development of the different species. The means were compared by the Tukey test. Vertical bars indicate the standard error of the means of five replicates.

In the four species the highest soluble lignin contents were found in new internodes (Fig. 4A), and *S. officinarum* had the highest content, which was equal among the others. *S. officinarum* also showed the highest content in mature culms. On the other hand, insoluble lignin content was higher in mature internodes in the four species (Fig. 4B), and in the two internode stages analyzed the highest contents were found in *S. spontaneum* and *S. robustum*.

### Saccharification yield

**Figure 5:**
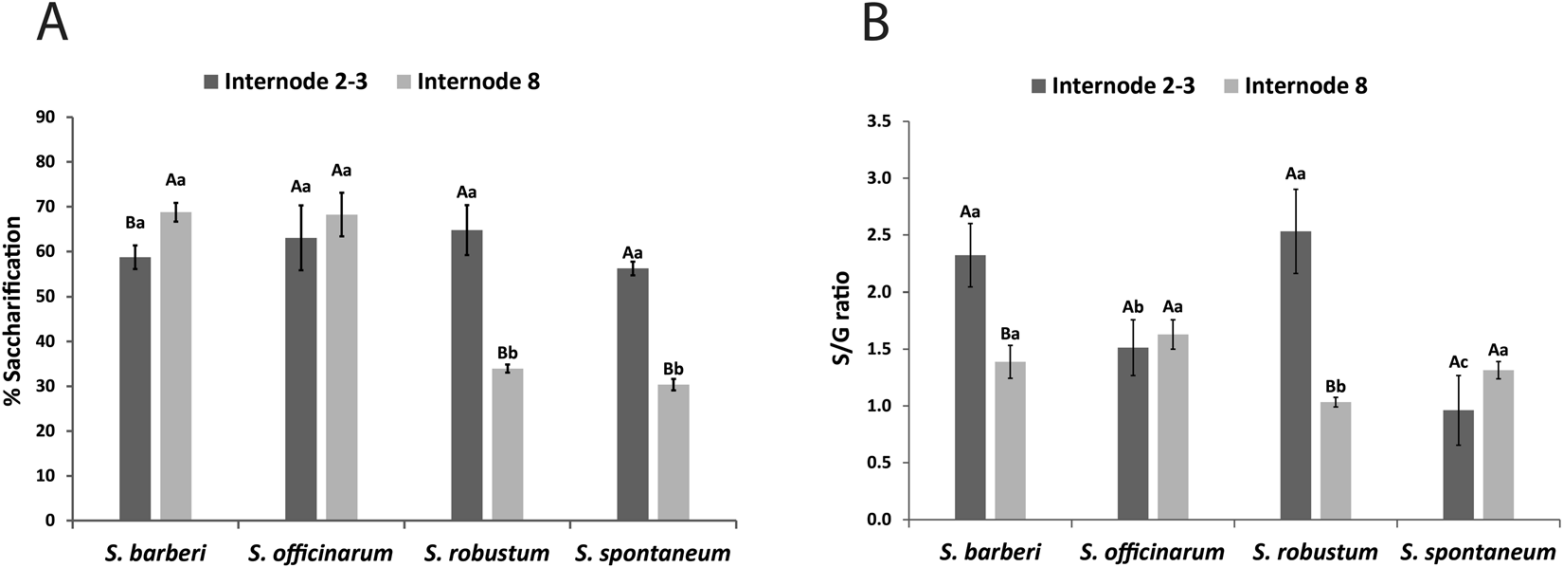
Saccharification yield (**A**) and S/G ratio (**B**) in internodes of *Saccharum* species. Different capital letters denote significant differences (p < 0.05) between internodes of different stages of development within the same species. Different lowercase letters indicate significant differences (p < 0.05) between internodes of the same stage of development of the different species. The means were compared by the Tukey test. Vertical bars indicate the standard error of the means of five replicates.

A similar pattern could be observed in saccharification yield of *S. barberi* and *S. officinarum*, and of *S. robustum* and *S. spontaneum*, constituting two distinct groups (Fig. 5A). While in the first two species the saccharification yields between the two stages of internodes were similar, they were quite different in the second group. Saccharification in mature internodes of S. *spontaneum* and *S. robustum* was nearly halfed that of young internodes. In general, the percentage of saccharification in young culms was close in the four species, around 65%.

### S/G ratio of lignin

S/G ratio was higher in new internodes than in mature internodes of *S. robustum* and *S. barberi* but did not differ in the other two species (Fig. 5B). When comparing only new internodes, the first two species also showed higher values than the other two. However, mature internodes of *S. barberi*, *S. officinarum*, and *S. spontaneum* showed higher values than that of *S. robustum*.

### Profile of soluble lignin oligomers

Soluble lignin monomers and oligomers were identified through comparison with data from a library^15^, using retention times, m/z ratio, and MS/MS fragmentation pattern. We found linkage structures belonging to the groups β-aryl ether (8*-*O-4), phenylcoumarin (8-5), and resinol (8-8), and it was possible to identify the S aromatic unit involved in each of these linkage structures (Table 1). In the species investigated we identified 11 structures: one aldehyde sinapyl, one monolignol (S), four dimers, and five trimers. Two of the dimers and two of the trimers presented 8-5 linkages, with G monomers, which are more recalcitrant linkages. The other linkages present in the trimers (8-*O*-4) are characterized as of easier cleavage. Two of the dimers (*m/z* = 357 [G(8-5)G] and *m/z* = 387 [S(8-5)G; S(8-8)G]) presented stereoisomerism (retention time = 3.39, 4.08 and 3.42, 3.39 respectively). This characteristic was also presented for two of the five trimers identified in this study (*m/z* = 583 [G(8-*O*-4)S(8-5)G] and *m/z* = 643 [S(8-*O-*4)S(8-8)S]), retention times: 3.57, 3.66 and 3.82, 4.2 respectively).

**Table 1:** Oligomer precursors of lignins, retention time and their respective *m/z* obtained by UPLC-MS/MS in internodes of *Saccharum* species.

Unit	Struture	*m/z*	Retention Time(min)
Monomers	Sinapylaldehyde Sinapylalcohol	207 209	3.26 2.75
Dímers	G(8-5)G	357	3.39/4.08
	G(8-O-4)G	375	3.76
	S(8-5)G; S(8-8)G	387	3.42 e 3.90
	G(8-O-4)S	405	3.20
Trímers	G(8-O-4)G(8-5)G	553	3.70
	G(8-O-4)S(8-5)G	583	3.57/3.66
	G(8-O-4)S(8-O-4)G	601	3.58
	S(8-O-4)S(8-O-4)G	631	3.65
	S(8-O-4)S(8-8)S	643	3.82/4.20

**Figure 6:**
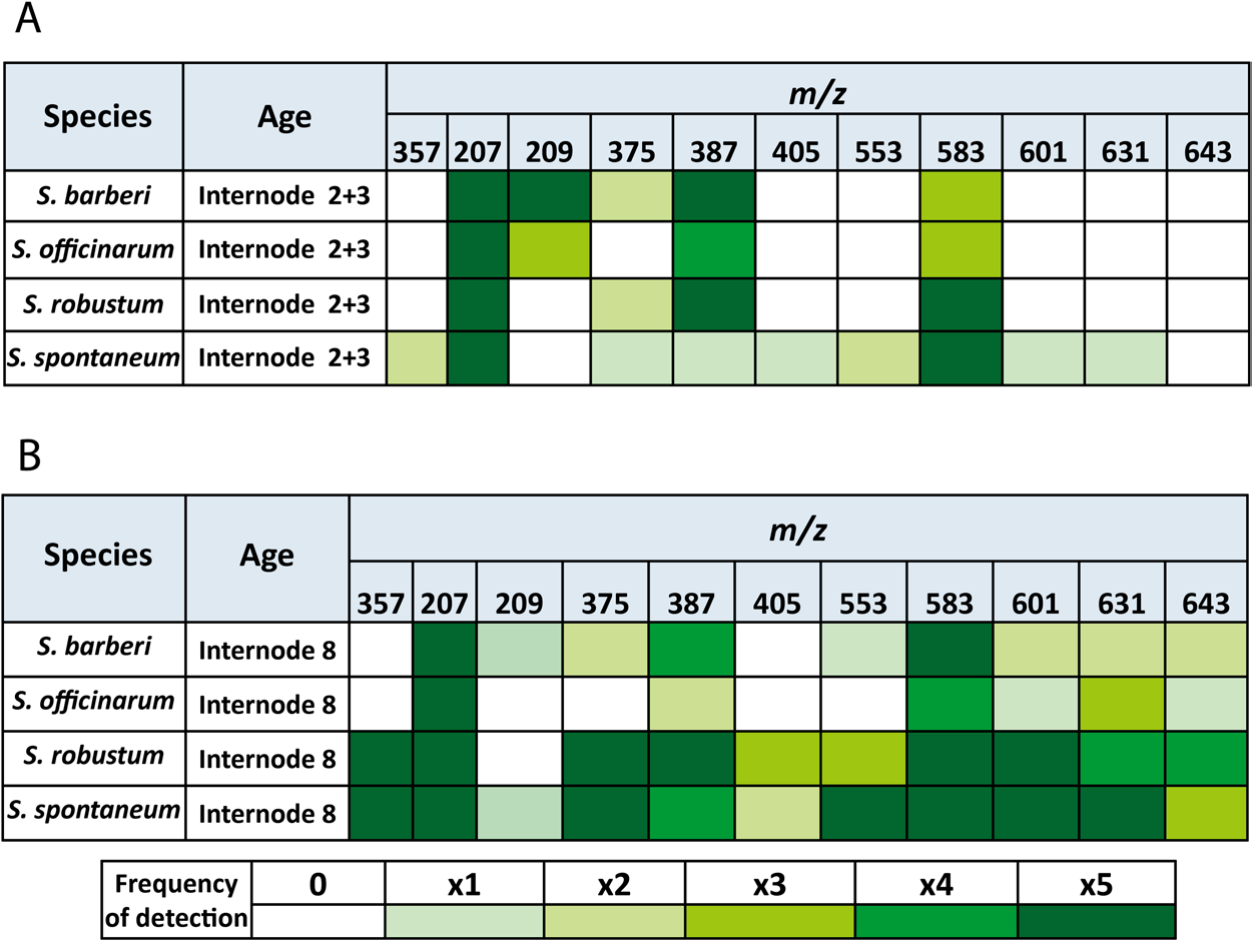
Distribution of lignin precursor oligomers and their respective *m/z* in internodes of different ages of *Saccharum* species. The frequency of each structure is represented in the diagram by different intensities of green colour, going from not found (0 - white) until found in all five samples analysed (x5 - intense dark green).

Figure 6 shows that, irrespective of internode age, *S. spontaneum* and *S. robustum* have the greatest diversity and frequency of oligomers compared with *S. officinarum* and *S. barberi*. Aldehyde sinapyl (*m/z* = 207) was found both in young internodes and in mature internodes of all species, whereas S monolignol (*m/z = *209) was found only in young internodes of *S. officinarum* and *S. barberi*, more frequently in the latter species. In the species *S. robustum* and *S. spontaneum*, lignin dimers show a tendency to be more frequent in mature internodes, contrary to what was found for *S. officinarum* and *S. barberi*, where dimers were more frequent in young internodes. Trimers were found preferably in mature internodes of the four species, and with remarkable frequency in *S. spontaneum* and *S. robustum*. Comparing all the oligomers identified, the dimer *m/z = *387 [S(8-5)G; S(8-8)G] and the trimer *m/z = *583 [G(8-*O*-4)S(8-5)G)] were the most frequent structures, and contrarily, the dimer *m/z* = 405 [G(8-*O*-4)S)] was the least frequent. The dimer *m/z* = 357 [G(8-5)G] was found only in the species *S. robustum* and *S. spontaneum*. The dimers *m/z* = 357 [G(8-5)G] and *m/z* = 405 [G(8-O-4)S)] were identified neither in the young internode nor in the mature internode of the species *S. officinarum* and *S. barberi*.

### Composition of monosaccharides, of lignin, and acetyl groups substituent of cell wall xylan

**Figure 7:**
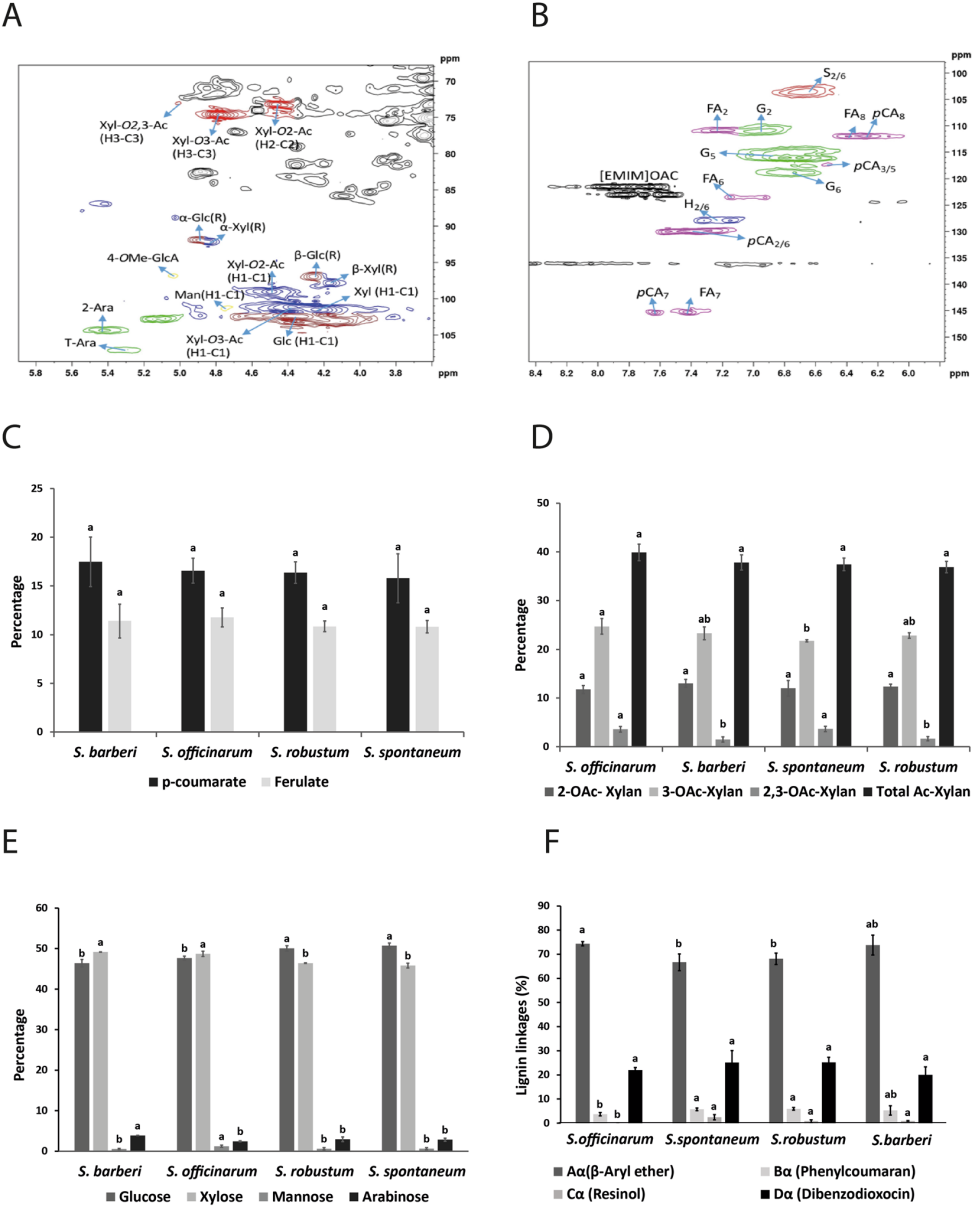
2D HSQC NMR (^1^H (x-axis)/^13^C (y-axis)) spectra of the anomeric region (**A**) and aromatic region of lignin (**B**) in shoots of *Saccharum* species. Percentage of *p*-hydroxycinnamates (**C**), O-acetyl substituents (**D**), monosaccharides (**E**) and lignin linkages (**F**) groups on stems of *Saccharum* species. The different lowercase letters indicate significant differences (p < 0.05) between the stems of *Saccharum* species for one type of *p*-hydroxycinnamate, O-acetyl substituent group or monosaccharide. The means were compared by the Tukey test. The vertical bars indicate the standard error of the means of three replicates.

Figure 7A,B show the expansion of the 2D-HSQC NMR spectrum (^1^H (x-axis)/^13^C (y-axis)) of the lignin aromatic region and anomeric region, respectively, of a stem wall sample, taking as example one of the *Saccharum* species. Prominent peaks corresponding to known polysaccharide linkages connections are tagged^56,57^. The compositions of *p*-hydroxycinnamates, O-acetyl substituent groups in xylan, and monosaccharides are shown in Fig. 7C–E. There was no significant difference as to p-coumarate and ferulate (Fig. 7C). In relation to the relative abundance of O-acetyl substituent groups of xylans (Fig. 7D), *S. officinarum* had a significantly higher percentage of 3-O-Ac substituent groups in xylan in relation to the other species under study. On the other hand, as for the 2,3-O-Ac group, there were significant differences between the species under study, and the highest percentage was found in *S. officinarum* and *S. spontaneum*. There were no significant differences between the species under study in relation to the total relative abundance of the acetylated groups and of the 2-O-Ac substituent group. *S. spontaneum* and *S. robustum* showed significantly highest glucose content, in relation to the species S. *officinarum* and S. *barberi*. In opposition to what was found for glucose, xylose percentage was significantly higher in the species *S. officinarum* and *S. barberi*. *S. officinarum* presents significantly greatest abundance of mannose when compared with the other species under study. As for the case of arabinose, *S. barberi* was the species that presented the highest percentage of this monosaccharide. *S. spontaneum*, *S. robustum*, and *S. officinarum* showed no significant differences with respect to the monosaccharide arabinose (Fig. 7E). β aryl ether and dibenzodioxocin were the main linkages detected in the four species while resinol and phenylcoumaran were found in lower amounts (Fig. 7F). *S. officinarum* showed the highest percentage for β aryl ether and the lowest for resinol and phenycoumaran.

### Histochemical analyses

**Figure 8:**
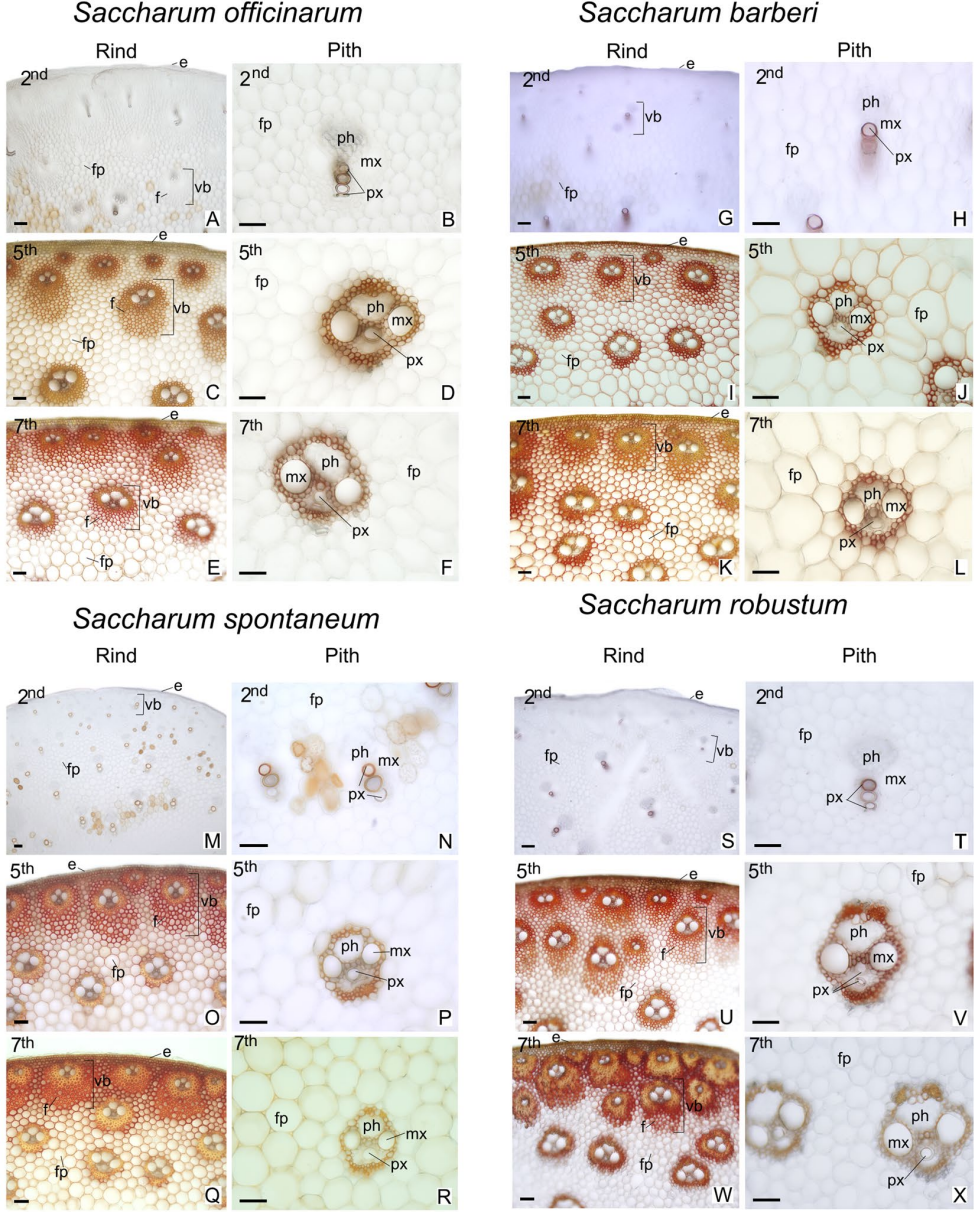
Cross sections of different regions and internodes of *Saccharum* species submitted to Maüle reaction for detection of lignin S and G. 2nd = immature internode, 5th = intermediate internode, 7th = mature internode. Right columns: peripheral region (Rind); Left Columns = central region (Pith). e = epidermis; f = fibers; fp = fundamental parenchyma; mx = metaxylem; ph = phloem; px = protoxylem; vb = vascular bundle. Scale bars = 50 μm.

**Figure 9:**
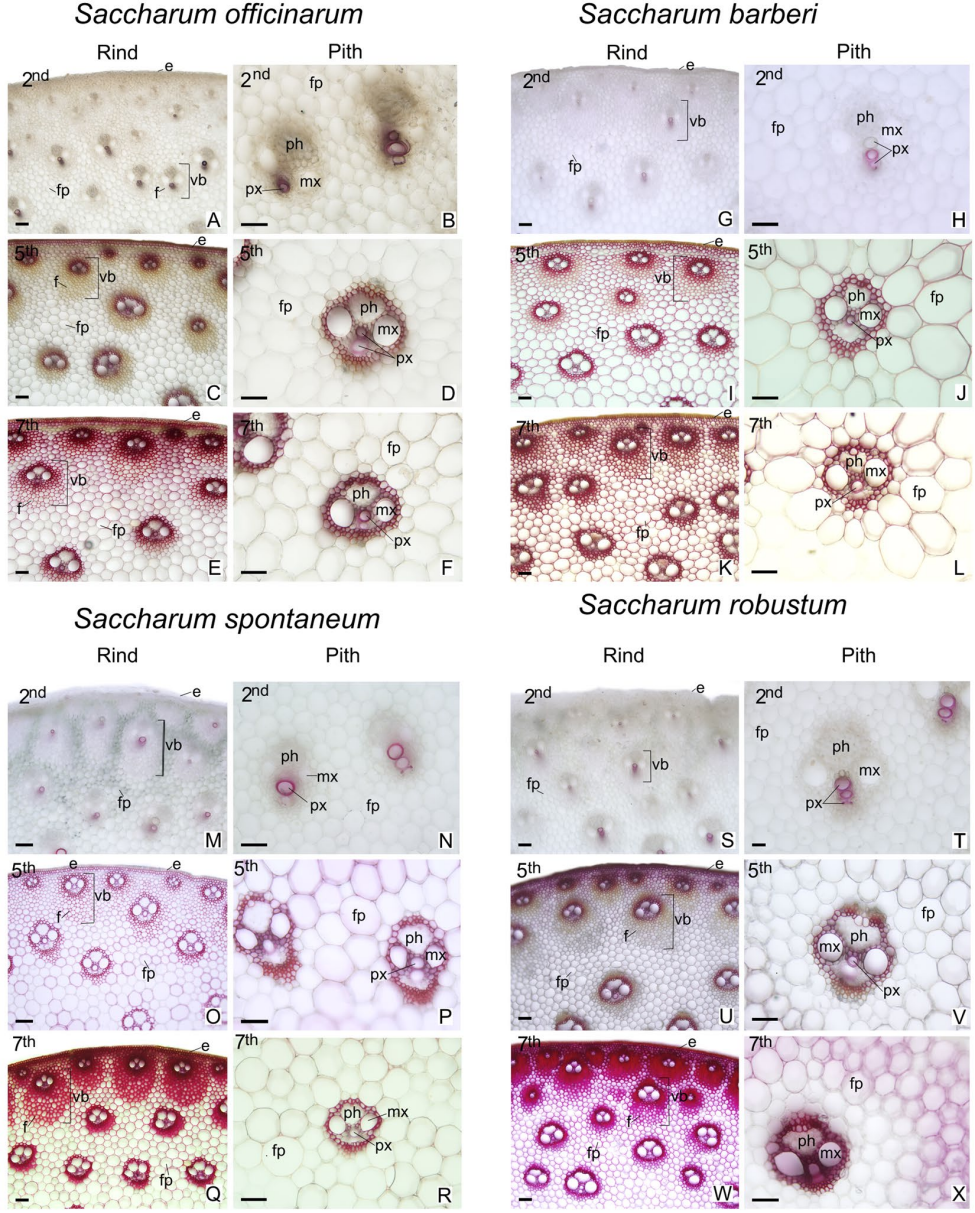
Cross sections of different regions and internodes in *Saccharum* species with Fluoroglucinol reagent for total lignin detection. 2nd = immature internode, 5th = intermediate internode, 7th = mature internode. Right columns: peripheral region (Rind); Left Columns = central region (Pith). e = epidermis; f = fibers; fp = fundamental parenchyma; mx = metaxylem; ph = phloem; px = protoxylem; vb = vascular bundle. Scale bars = 50 μm.

In all species analyzed the peripheral region adjacent to the epidermis presented a greater concentration of vascular bundles wrapped in several layers of fibers (Figs 8 and 9; left columns). In the central region of the culm, the species have vascular bundles scattered between fundamental parenchyma cells (Figs 8 and 9; right columns). In the second internode just below the stem apex, the tissues are in differentiation. In the xylem only the conducting cells of the protoxylem are differentiated and with lignified secondary cell wall (Figs 8 and 9; A-B, G-H, M-N, S-T). The epidermis and vascular bundles are differentiated in the fifth internode. The fibers around the bundles already show early deposition of secondary cell wall (Figs 8 and 9; C-D, I-J, O-P, U-V). In the seventh internode the tissues are differentiated and the parenchyma cells are completely expanded (Figs 8 and 9; E-F, K-L, Q-R, W-X). The Phloroglucinol-HCl reagent evidences the presence of lignin with red coloration (Fig. 9) and the Maüle reagent evidences syringyl (S) lignin with red coloration and guaiacyl (G) lignin with yellow coloration (Fig. 8). In the different stages of development analyzed, there was an increase in tissue lignification in the fifth and seventh internodes, with the second internode, still immature, showing little lignification. In the seventh internode of *S. officinarum* (Fig. 8E) and *S. barberi* (Fig. 8K) parenchyma cells and cells of the vascular bundles of the peripheral region of the culm show a predominance of red coloration with Maüle reagent, indicating S lignin. In the central region, parenchyma cells of *S. officinarum* are not lignified (Fig. 8F) and in *S. barberi* they are lignified and have yellowish coloration, indicating G lignin (Fig. 8L). In the seventh internode of *S. spontaneum* (Fig. 8Q) and *S. robustum* (Fig. 8W) there are fibers in the peripheral region of the vascular bundles, on which there is lignin deposition. The innermost fiber layers of the bundles show yellowish coloration, indicating G lignin, while the outermost layers have reddish coloration, of S lignin. In the two species, the fibers of the vascular bundles and parenchyma cells of the central region show yellowish coloration of G lignin (Fig. 8R,X).

**Figure 10:**
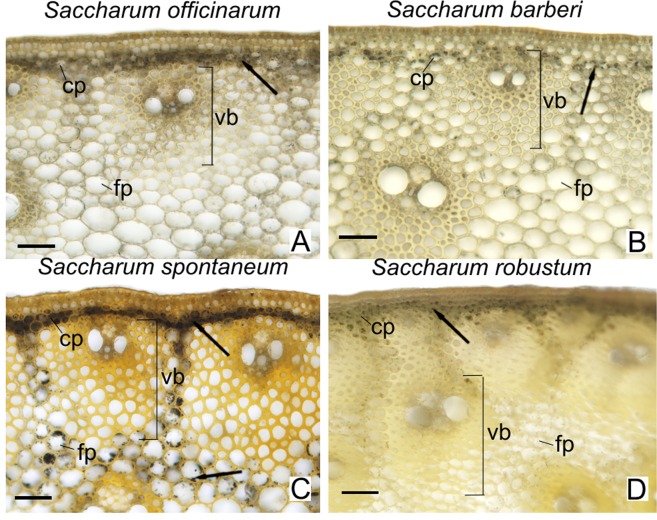
Cross sections of the stem peripheral region at the 7th internode of *Saccharum* species treated with lugol (I2 + KI) for detection of starch grains. Cp = chlorophyll parenchyma; fp = fundamental parenchyma; vb = vascular bundle; arrow = starch grains. Scale bars = 50 μm.

Starch grains, stained black, were observed in the chlorophyll parenchyma cells in the peripheral region of the culm of all species analyzed (Fig. 10). However, in the fundamental parenchyma cells the starch grains were only observed in abundance in *S. spontaneum* (Fig. 10C).

The marked differences found between the species were the thickness of the cell wall of the fibers of the vascular bundles in the peripheral region and the lignification of the parenchyma cells in the central region. In *S. officinarum* (Fig. 9E,F) and *S. barberi* (Fig. 9K,L) the vascular bundles near the epidermis presented fibers with thinner cell wall compared with those present in *S. spontaneum* (Fig. 9Q,R) and *S. robustum* (Fig. 9W,X). In the peripheral region, parenchyma cells of all species are lignified on the seventh internode. However, in the central region of the *S. officinarum* culm the parenchyma cells remain non-lignified.

### Identification and expression of monolignol biosynthesis genes

Bands taken from the gels and sequenced enabled the identification of 13 unigenes in the four *Saccharum* species: 1 *C4H*, 2 *4CL*, 1 *HCT*, 1 *F5H*, 1 *C3H*, 2 *CCoAOMT*, 1 *CCR*, 1 *COMT*, and 3 *CAD*. As two genes were isolated for *CCoAOMT* and *4CL*, they were identified as A and B; and for *CAD* they were called A, B, and C. The *SAS* (Sugarcane Assembled Sequences) of the respective orthologs in sugarcane identified by Bottcher *et al*.^33^ and the abundances of reads observed for each one of the genes identified in this study are shown in Supplementary Table S3. The phylogenetic analyses of the sequences of the genes isolated from the *Saccharum* species of this study and other angiosperms are in Supplementary Figs S1–S9 and the translated sequences for proteins are in Supplementary Figs S10–S18.

### **Gene expression profile in*S. spontaneum*and*S. officinarum***

**Figure 11:**
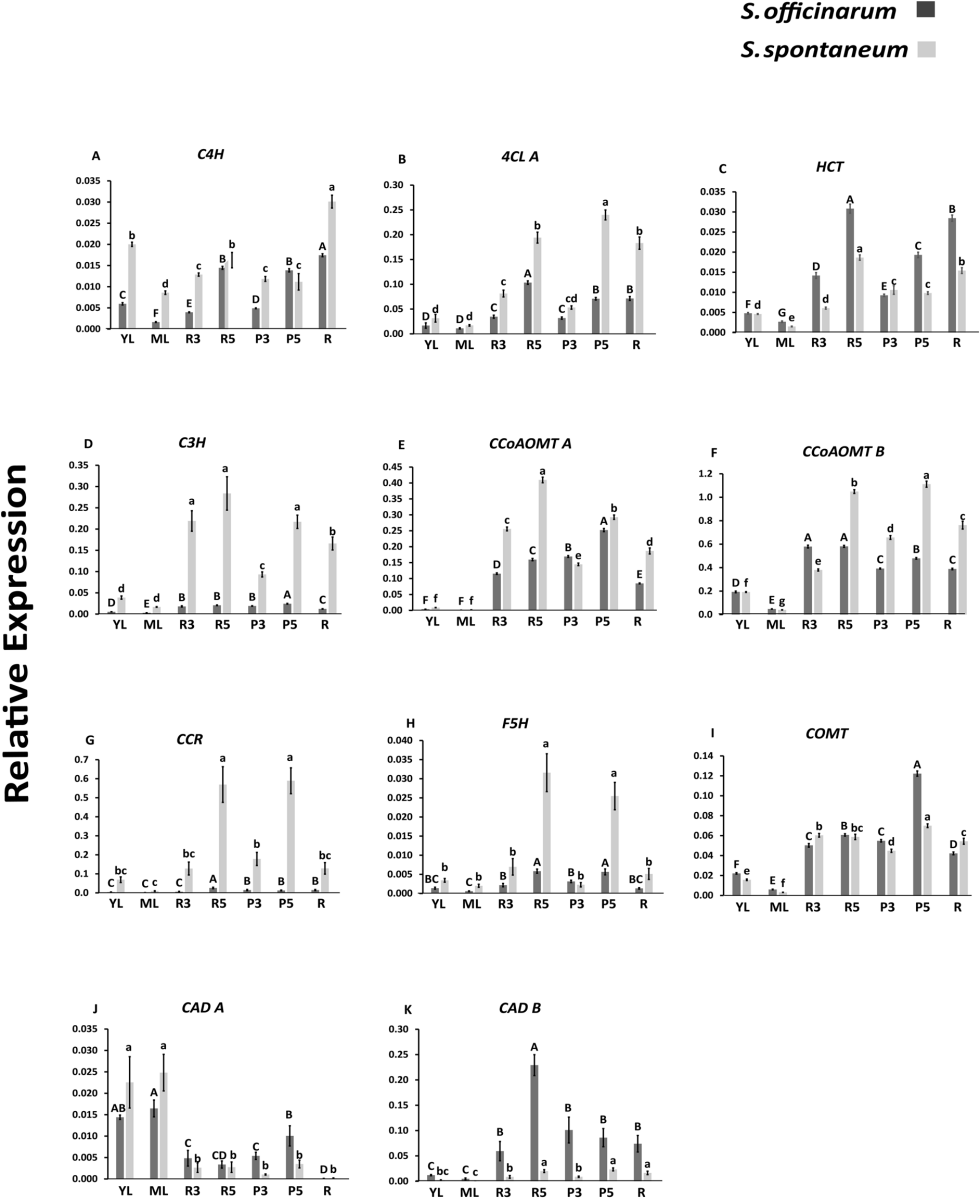
Expression profile of the genes of the biosynthetic pathway of the monolignols analysed by qRT-PCR in *Saccharum* species. YL = young Leaf, ML = mature Leaf, R3 = rind of the internode 3, R5 = rind of the internode 5, P3 = pith of the internode 3, P5 = pith of the internode 5 and R = root. Different letters indicate significant differences (p < 0.05) in relative gene expression among tissues of the same genotype. The means were compared by Tukey’s post-hoc test. The vertical bars indicate the standard deviation of the means of three biological replicates.

Expression of the identified genes were analyzed by qPCR (Fig. 11). In general, most genes were higher expressed in *S. spontaneum*, namely: *C4H*, *4CL A*, *C3H*, *CCoAOMT A* and *B*, *CCR*, and *F5H. S. officinarum* had higher expression of *HCT*, *COMT*, and *CAD B* genes. The *CAD A* gene had varied expression between the tissues, but its highest expression was in young and mature leaf (Fig. 11J). Internodes 3 and 5 showed a difference in rind and pith. *C4H* was equally expressed in pith and rind of *S. officinarum* and decreased from rind to pith in *S. spontaneum* (Fig. 11A). *4CL A* showed no difference between rind and pith in internode 3 in both species, but decreased from rind to pith in internode 5 for *S. officinarum* and increased for *S. spontaneum* (Fig. 11B). *HCT* had higher expression in rind of internodes 3 and 5, but compared with pith the expression in this tissue was lower (Fig. 11C). *C3H* had higher expression in all tissues of *S. spontaneum* compared with *S. officinarum*. The expression of *C3H* was higher in rind than in pith of internode 3 (Fig. 11D). *CCoAOMT A* had higher expression in rind and pith in internode 5 than in internode 3 (Fig. 11E). However, this gene was more expressed in pith (internodes 3 and 5) than in rind in *S. officinarum* and the opposite was observed in *S. spontaneum*. *CCoAOMT B* maintained the expression in rind of internodes 3 and 5 and increased slightly between pith 3 to 5 in *S. officinarum*, in *S. spontaneum* this gene was more expressed in tissues of internode 5 than 3 (Fig. 11F). *CCR* had relatively higher expression in rind and pith in internode 5 in *S. spontaneum* (Fig. 11G). In *S. officinarum* the expression was lower in all tissues, and there was higher expression in rind of internode 5 than in rind of internode 3. Among the genes analyzed *CCR* was one of the most expressed of the lignin biosynthetic pathway, followed by *CCoAOMT B* (Fig. 11F–G). *S. officinarum* showed no difference in expression between rind and pith for internodes 3 and 5 for *F5H*, but higher expression in *S. spontaneum* in rind and pith in internode 5 (Fig. 11H). In *COMT* a higher expression in pith of internode 5 for both species should be noted (Fig. 11I). *CAD* A presented a more specific pattern in young and mature leaves, low expression in roots for both species, and higher expression in pith for internodes 3 and 5 compared with rind, respectively of each internode in *S. officinarum* (Fig. 11J). No differences were observed in the expression of *CAD* A between rind and pith for *S. spontaneum*. *CAD* B in *S. officinarum* showed higher expression in rind of internode 5. In *S. spontaneum* there was higher expression in tissues of internode 5 compared with internode 3 (Fig. 11K). Interestingly, the genes display distinct pattern of expression in the two species, which shows a complex and distinct pattern in the control of the lignin biosynthetic pathway. The *CCR* and *CCoAOMT* B genes were expressed the highest, *4CL* and *F5H* displayed higher expression in more developed tissues, i.e., internode 5; *C3H* and *CCR* in *S. spontaneum*; *CAD* B in *S. officinarum*.

## Discussion

Sugarcane has the capacity of storing soluble, readily fermentable sugars (mostly sucrose) up to 18% of the fresh mass in the stalk^2,58^. The large accumulation of sucrose occurs in the maturation of the culms. Energy cane accumulates half or less sucrose than sugarcane and much of the fixed carbon is shuttled to structural polysaccharides such as cellulose and hemicelluloses^59^. By comparing the mature internodes between the *Saccharum* species studied, the lowest values for cellulose, hemicellulose, and pectin were found in the species *S. officinarum*, and the highest values were found in *S. spontaneum* (Fig. 1). The opposite was observed for sucrose, the primary soluble sugar in mature culms (Fig. 2). With some variation, *S. barberi* had closer levels to those of *S. officinarum*, while *S. robustum* was closer to *S. spontaneum*. This inverse relationship appears to be reflected in the wall monosaccharide composition evaluated by 2D-HSQC NMR spectroscopy. *S. officinarum* and *S. barberi* biomass harbor a higher xylose content, while *S. spontaneum* and *S. robustum* a higher glucose content (Fig. 7D) reflecting the competing sinks for these carbohydrates, hemicellulose and cellulose, respectively^60,61^.

Interestingly, while the cellulose content remained the same in new and mature culms of *S. barberi* and *S. officinarum*, it increased in the other two species. This behavior is opposite to the sucrose levels, that is, the disaccharide increases with maturation in the culms of *S. barberi* and *S. officinarum*, but remains practically the same in *S. robustum* and *S. spontaneum*. On the other hand, the comparison of reducing sugar contents in new and mature culms shows a much greater variation for *S. barberi* and *S. officinarum*, suggesting that reducing sugars in these species are directed towards sucrose synthesis, whereas in the other two species towards structural polysaccharides, in particular cellulose^62^. Similar to *Panicum virgatum*^63,64^, *Brachypodium distachyon*^60,65^, and *Zea mays*^66,67^, during the development of the internodes in *S. spontaneum* and *S. robustum* there was higher accumulation of carbon as unsoluble polysaccharides (cellulose, hemicellulose and pectin) in the cell wall, than the soluble sucrose in the parenchymal cell.

While the starch content was reduced during the maturation of the culms in *S. officinarum*, *S. robustum*, and *S. barberi*, it increased notably in *S. spontaneum* as also visually observed in the histochemical analyses. Starch granules were detected in the fundamental parenchyma of mature internodes of *S. spontaneum*.The presence of starch in *S. spontaneum* had been reported previously^59^, where 215 clones related to the genera *Saccharum*, *Erianthus*, and *Miscanthus* were analyzed. While *S. robustum* was the species with only traces of starch, *S. spontaneum* harbors the highest content. It has been suggested that the accumulation of starch in mature internodes of this species could be due to its capacity for tillering and high metabolic activity and as a strategy to cope with biotic and abiotic stresses^68^.

Lignin is the second largest biopolymer present in the cell walls of grasses^69^. Although it is essential for plant growth and development, lignin is the main factor responsible for the recalcitrance to processing of plant biomass in 2GE, including sugarcane^33^. Lignin content in the *Saccharum* species was determined using the Klason method, which distinguishes the soluble and insoluble fractions together providing a total estimate of lignin^70^. Regarding internode age a negative correlation was observed between these two types of Klason lignin, indicating greater amount of soluble Klason lignin (monomers and oligomers precursors of insoluble lignin polymers) in young internodes, and insoluble lignin in mature internodes. This is not surprising as lignification of the wall is still underway in young internodes. However, most of the lignin biosynthetic genes analyzed had a lower expression in young culms suggesting that the larger amount of soluble lignin in these tissues would be correlated to the polymerization process and not with monolignol production.

In the culm, the rind contains a high percentage of densely packed vascular bundles and is a metabolically active region with high peroxidase activity, therefore polymerizing and thus accumulating lignin^31,33^. When comparing the insoluble lignin content in mature internodes of the four species, *S. spontaneum* (20%) and *S. robustum* (18%) contain higher values than *S. barberi* (16%) and *S. officinarum* (14.5%). This difference was also observed in the histochemical analyses with phloroglucinol-HCl. Compared with *S. officinarum* and *S. barberi*, the rinds of mature internodes of *S. spontaneum* and *S. robustum* have higher density of vascular bundles and the walls of cellular elements such as hypodermis, epidermis, sclerenchyma and vascular fibers seem thicker and more lignified, contributing significantly to the higher content of this polymer. A general analysis of the expression of lignin biosynthesis pathway genes in the tissues of the culms displays a higher expression in *S. spontaneum* compared to *S. officinarum*, and a higher expression in tissues (rind and pith) of internode 5 compared with internode 3, supporting the higher insoluble lignin content in *S. spontaneum* and in mature tissues of the stalk. These gene expression differences, however, varied slightly depending on the species and tissue, for example, *C4H* in *S. spontaneum*, *C3H* in pith of the two internodes, *CAD* A and *CAD* B in rind and pith of *S. officinarum*, *CCoAOMT* A in rind of *S. officinarum*, and *HCT* in pith of *S. spontaneum*.

The nature of inter-monomeric linkages between lignin oligomers and their modifications can be exploited for the production of more degradable lignins^15,71,72^ enabling greater efficiency in fermentation process using cell wall sugars for 2GE production. The linkages 8-O-4 (β aryl ether) are the most common and are characterized as those of easiest cleavage. Lignins rich in G units have more recalcitrant linkages, such as 8-5 (phenylcoumarins), 5-5 (resinols), and 5-O-4, while S lignins are less interlinked and less recalcitrant to hydrolysis^15,73^. Overall, the analyses of the profiles of oligomers obtained by UPLC/MS from the four species studied identified 11 structures, between aldehydes, monomers, dimers, and trimers (Table 1). The distribution of these structures allowed a clear distinction between the internodes of the *Saccharum* species, and there was higher frequency of lignin oligomers in mature internodes than in young internodes. On the other hand, the highest amount of soluble phenols in all species were found in young culms, with markedly higher quantities in *S. robustum* and *S. spontaneum* compared with the other two species. Large quantities of free phenols, such as hydroxynnamic acids and chlorogenic acids, are found in tissues in lignification^10,16,25^. Also mature internodes of *S. robustum* and *S. spontaneum* the highest frequency and diversity of lignin oligomers (dimers and trimers) were found. Morreel *et al*.^74^ commented that the various lignin oligomers in tissues that undergo extensive lignification are derived from the availability of monolignols that are coupled under oxidative conditions for cell wall lignification, justifying the correlation between lignin content and frequency of oligomers.

The 8-O-4 linkage was the most common type of lignin linkage (Fig. 7F). According to Santos *et al*.^75^, this type of linkage is dominant in lignins of grasses, corresponding to 60% of the total. Other works such as those presented by Bottcher *et al*.^33^ and Kiyota *et al*.^15^ also corroborate these results. It was also observed that G units were found more frequently than S units in oligomers of the four *Saccharum* species. We could not find H units, although the lignin of grasses is characterized by having more of these units than the lignin of dicotyledons^76^. The non-detection of H units in the *Saccharum* species could be explained by the fact that these units occur essentially as free terminal, inert phenolic groups, and their incorporation prevent the growth of the lignin polymer. Due to their high oxidative potential they are insoluble in ethyl acetate, which was the solvent used in the extraction of the oligomers^76^. Linkages containing only G units in this study, such as the dimers *m/z = *357 [G(8-5)G] and *m/z = *375 [G(8-O-4)G] and the trimer *m/z = *553 [G(8-O-4)G(8-5)G], were identified notably in mature internodes of *S. spontaneum* and *S. robustum*. This result might explain why mature internodes of these species showed a lower S/G ratio than mature internodes of *S. officinarum* and *S. barberi*. The structures corresponding to the dimers *m/z = *387 [S(8-5)G] and [S(8-8)G] and the trimer *m/z = *583 [G(8-O-4)S(8-5)G] were identified in all internodes, which suggests that these structures are conserved and fulfil an important role in the growth and development. Sinapyl alcohol (S) (*m/z* = 209) was found more frequently in young internodes of the species *S. officinarum* and *S. barberi*. Young internodes of *S. officinarum* and *S. barberi* have a higher soluble Klason lignin content than the other *Saccharum* species, which could be correlated with an increased frequency of the structure *m/z = *209 as soluble Klason lignins are primarily composed of S units^77^. In comparison to the studies of Bottcher *et al*.^33^ and Kiyota *et al*.^15^, only two structures *m/z* = 357 [G(8-5)G] and *m/z* = 583 [G(8-O-4)S(8-5)G] were always present indicating that they are conserved among the species of the genus *Saccharum*.

In sugarcane hybrids it has been observed that the S/G ratio increases with the development of the stalk^33^. The same was observed in other grasses such as *Festuca arundinacea*^78^, *Zea mays*^66^, and *Panicum virgatum*^79^. However, such a ratio increase was not observed here, with the S/G ratio being higher in young internodes of *S. barberi* and *S. robustum* and equal for the other two species. Local growth conditions may have affected the S/G ratio, but in the case of the *S. barberi* and *S. robustum* the low values might be due to the amount of one of the monomers being higher in the pith or rind. We did not separate rind and pith for S/G analysis but based on the histochemical analysis the G amount (stained yellow, see Fig. 8) was elevated in the pith compared to the rind.

Lignin composition (S/G ratio) affects the yield of saccharification^7^ since tissues rich in S are more susceptible to hydrolysis than those rich in G^80^. We found no significant difference in saccharification in young internodes of the four species studied, which is not unexpected, since the lignification process has not been completed based on the content of soluble and insoluble lignin, oligomers, and phenols. However, it is interesting to note that in young tissues there seems to be no relationship between saccharification yield and S/G ratio, since *S. barberi* and *S. robustum* have higher S/G ratio, but saccharification yield is equal. However, mature internodes of *S. spontaneum* and *S. robustum* with lower S/G ratio resulted in a lower yield of saccharification. Therefore, higher yield of saccharification is related to S/G ratio, but only in tissues whose maturity has been reached and, thus, where the secondary cell wall formation process has been completed.

F5H and COMT are thought to be the determinant enzymes in defining S unit content in plants^38,81^. In *P. radiata*, the joint action of the two activities led to an increase of S units, with the increase being smaller when only *F5H* was overexpressed^81^. In sugarcane, the reduction in the expression of *COMT* and *F5H* using RNAi led to different situations^38^. While plants with partial silenced *F5H* did not show a reduction in lignin content, one of the lines had a reduced S/G ratio with a concomitant increased saccharification yield. One of the mutants of *COMT* displayed a reduction in lignin content and improvement in saccharification yield. One of the mutants of *COMT* exhibited a reduction in the S/G ratio. Our data do not indicate a direct relationship between the expression of *COMT* and *F5H* and the S/G ratio. Using *S. spontaneum* as an example, this species had a similar S/G ratio between young and mature internodes; however, the expression of *COMT* and *F5H* was a little higher in pith of mature internodes but equal to the rind of young and mature internodes. On the other hand, the expression of *F5H* was much higher in mature tissues. A similar situation was also observed in *S. officinarum*, but with lower expression values. It cannot be ruled out that other hitherto unidentified isoforms of *COMT* and *F5H* are involved in lignin biosynthesis in these two species, but it is noteworthy that Bottcher *et al*.^33^ isolated only one COMT and one F5H in sugarcane, and its sequences have a high homology with the sequences isolated in the four species studied.

Another factor that has been recognized as negatively affecting plant biomass processing into 2GE is the degree of *O*-acetylation of cell wall polymers, since acetate, when released during pretreatment represents a powerful inhibitor of fermenting microorganisms^82^. *O*-acetylation of hemicelluloses also reduced enzymatic hydrolysis due to steric hinderance of the acetate^83^. Therefore, reducing the content of *O*-acetyl groups in biomasses with bioenergetic potential is desirable^84^. The main hemicelluloses in grasses are xylans^3^ and their degree of *O*-acetylation may vary according to plant species, type of tissue and organ, and state of development^85^. Xylan acetylation occurs more frequently in position O-3 (up to 30%) and less frequently in O-2 (up to 25%), but acetylation in both positions has been reported^85^. In the *Saccharum* species studied here, it was found that the total percentage of acetylation (36.9–39.9%) was similar to values found in other grass biomasses^86^. On the other hand, acetylation in position O-3 was predominant (21.8–24.7%) with respect to the substitution O-2 (11.8–13.0%) and to O-2/O-3 (1.47–3.47%). Analyses by 2D-HSQC NMR spectroscopy showed that *S. robustum* and *S. spontaneum* were the species that presented the lowest percentages of acetylation in position O-3 (21.8% and 22.9%, respectively) and total acetylation (36.9–37.4%, respectively). However, the hypothesis that biomass with a reduced percentage of acetylesters results in higher saccharification yields^87^ could not be supported here. *S. officinarum* and *S. barberi*, with a higher degree of acetylation than *S. spontaneum* and *S. robustum*, exhibited a higher yield of saccharification. Since it is known that in secondary walls xylans are closely associated with cellulose^88^, a lower percentage of acetyl groups in *S. spontaneum* and *S. robustum* could lead to an even tighter association of xylan with cellulose adding to recalcitrance in these species, and limiting the yield of saccharification^83^.

The strategy used in this study to identify genes involved in the lignin biosynthetic pathway in the four *Saccharum* species involved the amplification of fragments produced in RT-PCR reactions using primers designed from conserved regions of gene sequences of sugarcane and of several other close species. Therefore, such primers are likely to amplify sequences of closely related genes encoding similar enzymatic activities. There is a possibility that not all genes of a gene family are amplified. However, the isolated genes represent the highest expressed genes in the tissues is high. Taking into account that the four species studied presented distinct genetic characteristics, it was surprising to observe that the isolated sequences are highly similar among the species and very close in sequence to the ones identified by Bottcher *et al*.^33^ in sugarcane. Such similarities could be explained not only by the evolution of the lignin biosynthetic pathway in terrestrial plants but also by the origin of the genus *Saccharum* and of the commercial cultivars of sugarcane. The parental genomes of *S. officinarum* (80–90%) and *S. spontaneum* (10–20%) contributed to sugarcane hybrids including to some extent recombinant chromosomes^89^. Additionally, the lignin biosynthetic pathway is very conserved between plants and modifications in this pathway generate similar phenotypes between monocotyledons and dicotyledons. The approaches to manipulate lignin in alfalfa^7^ can be transferred to other species such as switchgrass and sugarcane^29,37^. Genes related to sugar accumulation in sugarcane culms arose through differential expression of other regulators suggesting a specific epigenetic control. PAL is highly conserved between plants and seems to precede the divergence of dicotyledons and monocotyledons^90^. Genes related to transcriptional activation are highly conserved in grasses^91^. An example is the gene SND1 which activates several transcription factors: SND3, MYB46, MYB83, MYB85, and MYB105; apparently very conserved during evolution^91^.

## Conclusions

The set of data obtained here enabled the association of patterns to better understand the process of lignin deposition in four *Saccharum* species. The differences between the species studied became evident, whether in relation to structural and non-structural carbohydrates or in the quantity and type of lignin. The data enabled the coherent separation of the two species that have been identified as energy canes, *S. spontaneum* and *S. robustum*, which accumulate more fiber, from the other two, which accumulate more sucrose. Moreover, the first two species contain more insoluble lignin, the lowest S/G ratios, greater abundance of intermonomeric linkages (lignin oligomers), and lower percentages of saccharification. Gene expression analysis of the lignin biosynthesis pathway genes in *S. officinarum* and *S. spontaneum* showed that in general the later species has higher expression in culm tissues especially in mature culms. Surprisingly the sequences of the identified genes showed high conservation in the four *Saccharum* species including the commercial hybrids. This feature is desirable for the genetic manipulation of energy cane, since knowledge has already been gained with low lignin commercial varieties of sugarcane^39,44,92,93^. It has been show in other grasses that lignin biosynthesis has a complex regulation by transcription factors, which can activate or repress the expression of the several genes of the route^93–98^. However, to our knowledge, this is the first report describing that lignin genes are highly conserved among species of the same genus and, consequently, the differences they have regarding the polymer content and composition can be only fully understood after gaining knowledge on the sequencing of the regulatory regions of each gene or at least of a set of genes.

## Methods

### Plant material and growing conditions

Culms of the species *S. spontaneum, S. officinarum, S. robustum*, and *S. barberi* were obtained from the Center of Sugarcane of the Agronomy Institute of Campinas, at Ribeirão Preto, São Paulo State, Brazil. The culms were planted in plastic trays containing vermiculite and kept in a greenhouse and the resulting seedlings were transplanted to 50 L pots containing commercial organic substrate and kept in the greenhouse for approximately one year. For each species 5 replicates were planted (5 pots). After this period, the substrate of the pots was partially replaced, taking care not to damage the root system, and the pots were transferred out of the greenhouse, to the experimental area of our department, under natural sunlight. The pots remained in these conditions for a period of 4 months, with daily irrigation.

Only healthy stems, without any sign of physical injury or disease were collected. For biochemical analyses internodes 2 + 3 (young stage) and internode 8 (mature stage) were separated from the apex. Histochemical analyses were performed on internodes 2, 5, and 7. Internodes 4 to 10 were used for cell wall characterization by 2D-HSQC NMR spectroscopy. To identify the genes of the lignin biosynthetic pathway we made a composite sample, containing a 1/1 (w/w) mixture of young internodes (2 + 3) and mature internodes (8), from five plants. For the expression analyses (quantitative RT-PCR, qPCR) 7 types of tissues were used: young and mature leaves, rinds of internodes 3 and 5, piths of internodes 3 and 5, and roots. A steel blade was used to separate the rind from the pith^31^. In the samplings, the stems were washed in tap water, chopped into small pieces of 1 cm^2^, frozen in liquid nitrogen and grinded and stored in freezer at −80 °C. For biochemical analyses the ground tissues were dried in a freeze-dryer.

### Histochemical analysis

Internodes 2, 5, and 7 of the stems of the four species were used in these analyses. Histochemical tests were made with hand cut sections of ~0.5 mm thickness using a steel blade. The following reagents were used to identify the cell wall components: lignin - fluoroglucinol-HCL^99^; syringyl and guaiacyl monomers - Maüle reagent^100^; and starch - Lugol’s iodine^101^. The staining results were obtained with an Olympus DP71 camera attached to an Olympus BX 51 microscope.

### Cell wall polysaccharides

The protocol of Chen *et al*.^78^ was followed and pectin, hemicellulose fraction and cellulose were determined. Total sugar content in each fraction was determined with phenol-sulfuric reagent, using glucose as standard^102^.

### Non-structural sugars and starch

Samples were extracted with 70% ethanol at 60 °C for three times and the supernatants were pooled after centrifugation. Total soluble sugars and sucrose were determined with the phenol- sulfuric assay^102,103^ and glucose and sucrose were used as standards, respectively. Reducing sugar content was determined according to Nelson^104^ using glucose as standard. Starch content was determined according to Amaral *et al*.^105^. The dried, 70% ethanol extracted samples were treated sequentially with α-amylase from *Bacillus licheniformis* (code E-ANAAM, MEGAZYME, Ireland) and amyloglucosidase from *Aspergillus niger* (code E-AMGPU, MEGAZYME, Ireland) and the resulting glucose was determined with the PAP Liquiform glucose kit (Labtest Diagnóstica S.A.), using an ELISA plate reader (model EL307C, Bio-Tek Instruments, Winooski, Vermont) at 490 nm. Glucose was used as standard.

### Analysis of wall constituents by 2D spectroscopy HSQC NMR

Ball-milled de-starched, alcohol insoluble material (25 mg) was dissolved in 0.75 mL of DMSO-d6 and 10 μL of [Emim] OAc-d14 as previously described^56^. The dissolved lignocellulosics were subjected to a 2D HSQC NMR experiment acquired on a Bruker AVANCE 600 MHz NMR spectrometer equipped with a 5-mm TXI ^1^H/^13^C/^15^N cryo-probe using the pulse sequence ‘hsqcetgpsisp.2’. The experiments were carried out at 25 °C with the following parameters: spectral width 12 ppm in F2 (^1^H) dimension with 4096 data points (TD1) and 160 ppm in F1 (^13^C) dimension with 256 data points (TD2); scan number (SN) of 200; inter scan delay (D1) of 1 s. The chemical shifts were referenced to the DMSO solvent peak (δ_C_ 39.5 ppm, δ_H_ 2.5 ppm). The NMR data was quantified as described previously using Bruker’s Topspin 3.1 software^56,57^. The acetylation on xylan was quantified as described below. In brief, the signals in the aromatic region (H1-C1 signals of 2-*O*-Ac-Xyl, 3-*O*-Ac-Xyl, 2,3-*O*-Ac-Xyl, Xyl (xylan) and reducing ends of Xylan (α/β-Xyl-R)) were summed up to 100%, and the signal in the aliphatic region were integrated separately to calculate the relative content of each form of O-acetyl- xylan unit. The relative content of 2-*O*-Acetyl and 2,3-*O*-Acetyl-Xylan units were calculated from H2-C2 signal and 3-*O*-Acetyl-Xylan unit were calculated from H3-C3 signal. The monosaccharide composition [glucose (Glu), xylose (Xyl) and mannose (Man)] was quantified from their anomeric integrals as a fraction of 100%. The compositions of lignin; S (syringyl), G (guaiacyl), H (*p*-Hydroxyphenyl), FA (ferulate) and pCA (*p*-coumarate) lignin units were quantified from their aromatic lignin integrals as a fraction of 100%.

### Total soluble phenols

The samples were extracted twice with 80% ethanol and the phenols extracted were determined with the Folin-Ciocalteu reagent^106^. Chlorogenic acid was used as standard.

### Lignin content, S/G ratio, and oligomers

Soluble and insoluble lignin was determined according to the TAPPI UM-250 Protocol^107^. Insoluble lignin content was expressed as percentage of dry wall residue, obtained after sample extraction and hydrolysis. For the determination of soluble lignin, the absorbance of the filtrate of the hydrolysis product was determined at 205 nm and the content calculated using an extinction coefficient of 110 l. g^−1^.cm^−1^. To determine the S/G ratio, the samples were treated with NaOH in a heating block at 95 °C/24 h, neutralized with HCl and extracted with ethyl acetate. The residue was dried and then dissolved in H_2_O MilliQ and the hydrolysis products were analyzed by LC-MS using a UHPLC coupled to a triple quadrupole mass spectrometer with ESI ionization source (model ACQUITY, Waters Corp., Manchester, UK), as described by Mokochinski *et al*.^108^. For the analysis of soluble lignin oligomers the samples were twice extracted in 80% ethanol under sonication and the extracts were dried in a concentrator (Concentrator plus-Eppendorf). The dried residue was solubilized in acetonitrile/water (1:2, v/v) just before the analyses. The samples were analyzed in an Acquity UPLC coupled to a TQD triple quadrupole mass spectrometer (Micromass-Waters, Manchester, UK), according to Kiyota *et al*.^15^.

### Saccharification

Saccharification was determined according as described by Brown and Torget^109^ using lyophilized biomass equivalent of 10 mg of cellulose. After addition of sodium citrate buffer (0.1 M, pH 4.8), Na_3_N, and H_2_O MilliQ, the mixture was heated to 50 °C and cellulase (*Trichoderma reesei*) and cellobiohydrolase (*Aspergillus niger*) was added at a 1:4 v/v ratio (Sigma-Aldrich). The samples were incubated in a 160 rpm shaker at 50 °C for 5 days, and then centrifuged at 12,000 rpm for 15 min. Glucose was quantified in the supernatant^102^.

### *In silico* analysis of databases and synthesis of primers for identification of expressed genes

We studied the genes of the following lignin biosynthesis enzymes: 4-hydroxicinnamoyl CoA: ligase (4CL; EC 6.2.1.12), cinnamoyl CoA reductase (CCR; EC 1.2.1.44), ferulate 5-hydroxylase (F5H; EC 1.14.13.-), caffeate O-methyltransferase (COMT; EC 2.1.1.68) cinnamyl alcohol dehydrogenase (CAD; EC 1.1.1.195), caffeoyl CoA 3-O-methyltransferase (CCoAOMT; EC 2.1.1.104), p-coumaroylshikimate 3′-hydroxylase (C3′H; EC 1.14.13.36), cinnamate 4- hydroxylase (C4H; EC 1.14.13.11), hydroxycinnamoyl-CoA: shikimate/quinate p-hydroxycinnamoyl-transferase (HCT; EC 2.3.1.133) The sequences of the genes characterized by Bottcher *et al*.^33^ were used as bait for the search for homologues in the NCBI and Phytozome databases. We selected sequences of sorghum (*Sorghum bicolor*), rice (*Oryza sativa*), corn (*Zea mays*), wheat (*Triticum aestivum*), *Lolium perenne*, and *Arabidopsis thaliana*. We used only full-CDS sequences with a low e-value (<10^−6^). These sequences were aligned in the BioEDIT program^110^ and conserved regions were used for the design of primers (Supplementary Table S1) using the Primer 3 program, having as parameters Tm 57 °C–60 °C, a difference of only 2 °C in Tm values between the primers of a pair and the GC content between 55% and 60%^111^. In some cases, degenerate primers were synthesized. The primers were made in regions that enabled amplifying as many ORFs as possible.

### Total RNA extraction, cDNA synthesis, amplification and sequencing

Total RNA extraction was performed in a 1:1 (w/w) mixture of tissues from young internodes (2 + 3) and mature internodes (8). Total RNA was extracted with Trizol (Tri-Phasis Reagent – BioAgency) and treated with Turbo DNAse-free (Ambion). First-strand cDNA synthesis was performed with SuperScript III (Invitrogen) following the manufacturers’ guidelines. RT-PCR reactions were carried out in a thermal cycler (Veriti 96-Well Thermal Cycler-AB Applied Biosystems) following the parameters of Llerena *et al*.^112^. The amplification products were separated by electrophoresis in a 1% agarose gel containing ethidium bromide and observed by a photo-documenter Gel Doc 2000 (Biorad). Bands with the expected number of bases were recovered from the gel with GeneJET Extraction (Thermo Scientific), inserted into the cloning vector pGEM-T easy (Promega), and cloned in thermocompetent *Escherichia coli* DH10β (Novagen). Some colonies were selected, and the presence of the insert (PureLink Quick Plasmid Miniprep Kit, Invitrogen) and its size were confirmed after digesting the plasmid with EcoRI. The inserts were sequenced using M13 primers. Sequencing reactions were performed using BigDye® Terminator v3.1 Cycle Sequencing Kit (Applied Biosystems) and 3730xl DNA analyzer sequencer (Applied Biosystems). Several colonies were sequenced until 25 good quality sequences (forward and reverse orientation for each sequence) were obtained.

### Phylogenetic analyses

The obtained nucleotide sequences were translated to amino acid sequences *in silico*, and homologous proteins obtained from databases NCBI (http://www.ncbi.nlm.nih.gov/), SUCEST (http://sucest-fun.org), and Phytozome (http://www.phytozome.net/) were selected for phylogenetic analysis. Multiple alignment of amino acid sequences was performed with the ClustalW program^113^. Phylogenetic analyses were performed with the MEGA program version 4.02 and evolutive relations were inferred using the Neighbor-joining algorithm with Bootstrap for 1,000 repetitions. Gap regions were excluded manually.

### Gene expression analysis of the isolated genes

For the gene expression analysis primers specific for the isolated gene sequences were designed (Supplementary Table S2). The efficiency curve of the primers was determined with the Step One Plus Software v2.3 (Life Technologies). Total RNA extraction and first-strand cDNA production were carried out as described above. cDNAs of 7 tissues (new leaf, old leaf, rinds of internodes 3 and 5, piths of internodes 3 and 5, and root) of the species *S. officinarum* and *S. spontaneum* were used in the analysis. The reactions were prepared with iTaq™ universal SYBR^®^ Green supermix (Bio-Rad) and analyzed in a StepOnePlus™ Real-Time PCR System, following the program of 95 °C for 3 min and 40 cycles of 95 °C for 10 s and 60 °C for 30 s. The specificity of the amplified products was evaluated by dissociation curve analysis generated by the equipment. *GAPDH* (glyceraldehyde 3-phosphate dehydrogenase) was used as housekeeping gene^33^. The relative expression was calculated by 2^−ΔCt^ according to Livak and Schmittgen^114^.

### Statistical analyses

For biochemical analyses we conducted factorial Analysis of Variance (ANOVA), where the first level are the species of *Saccharum* and the second level are the sugarcane maturation stages, i.e., young internodes (2 + 3) and mature internodes (8). Comparison between means was performed through the Tuckey test (*α* = 0.05). For gene expression analysis we used ANOVA and for comparison of means the Tuckey test (*α* = 0.05). For the biochemical analyses (soluble sugars, starch, cell wall polysaccharides, total phenols, Klason lignin, and saccharification) we analyzed 5 biological replicates with three technical replicates each. For the analysis of soluble lignin oligomers and S/G ratio, we analyzed 5 replicates and 1 technical replicate each. For the analyses of hydroxycinnamic acids, monosaccharides, and acetylated xylans we analyzed three biological replicates and one technical replicate each. Results of the biochemical analysis were expressed as mean ± standard error. For gene expression, the analyses were expressed as the mean for three biological replicates and three technical replicates each. For the control of error transfer in the calculation of gene expression we used a linear model of error accumulation $({\sigma }_{\Delta Ct}^{2}={\sigma }_{{\rm{C}}t,ref}^{2}+{\sigma }_{{\rm{C}}t}^{2})$ in the calculation of the ∆Ct value and a nonlinear model $({\sigma }_{{2}^{-\Delta Ct}}^{2}={(\frac{d[{2}^{-\Delta Ct}]}{d[\Delta Ct]})}^{2}{\sigma }_{\Delta Ct}^{2})$ in the calculation of the 2^−∆Ct^ value^115^.

## Supplementary information

Supplementary information 1

Supplementary dataset 1

## Data Availability

All data generated or analysed during this study are included in this article (and its Supplementary Information files).

## References

[1] Kang Q, Appels L, Tan T, Dewil R (2014). Bioethanol from lignocellulosic biomass: current findings determine research priorities. Sci. World J..

[2] de Souza AP, Grandis A, Leite DCC, Buckeridge MS (2014). Sugarcane as a bioenergy source: history, performance, and perspectives for second-generation bioethanol. BioEnergy Res.

[3] Pauly M, Keegstra K (2008). Cell-wall carbohydrates and their modification as a resource for biofuels. Plant J..

[4] Welker CM (2015). Engineering plant biomass lignin content and composition for biofuels and bioproducts. Energies.

[5] van der Weijde T (2013). The potential of C4 grasses for cellulosic biofuel production. Front. Plant Sci..

[6] Chandel AK (2014). Multi-scale structural and chemical analysis of sugarcane bagasse in the process of sequential acid–base pretreatment and ethanol production by *Scheffersomyces shehatae* and *Saccharomyces cerevisiae*. Biotechnol. Biofuels.

[7] Chen F, Dixon RA (2007). Lignin modification improves fermentable sugar yields for biofuel production. Nat. Biotechnol..

[8] Van Acker R (2013). Lignin biosynthesis perturbations affect secondary cell wall composition and saccharification yield in *Arabidopsis thaliana*. Biotechnol. Biofuels.

[9] Wilkerson CG (2014). Monolignol ferulate transferase introduces chemically labile linkages into the lignin backbone. Science (80-.).

[10] Boerjan W, Ralph J, Baucher M (2003). Lignin biosynthesis. Annu. Rev. Plant Biol..

[11] Wang Y, Chantreau M, Sibout R, Hawkins S (2013). Plant cell wall lignification and monolignol metabolism. Front. Plant Sci..

[12] Bonawitz ND, Chapple C (2010). The genetics of lignin biosynthesis: connecting genotype to phenotype. Annu. Rev. Genet..

[13] Cesarino I, Araújo P, Domingues Júnior AP, Mazzafera P (2012). An overview of lignin metabolism and its effect on biomass recalcitrance. Brazilian J. Bot.

[14] Vanholme R, Demedts B, Morreel K, Ralph J, Boerjan W (2010). Lignin biosynthesis and structure. Plant Physiol..

[15] Kiyota E, Mazzafera P, Sawaya ACHF (2012). Analysis of soluble lignin in sugarcane by ultrahigh performance liquid chromatography-tandem mass spectrometry with a do-it-yourself oligomer database. Anal. Chem..

[16] Raes J, Rohde A, Christensen JH, de Peer Y, Boerjan W (2003). Genome-wide characterization of the lignification toolbox in *Arabidopsis*. Plant Physiol..

[17] del Río JC (2015). Differences in the chemical structure of the lignins from sugarcane bagasse and straw. Biomass and Bioenergy.

[18] Lan W (2015). Tricin, a flavonoid monomer in monocot lignification. Plant Physiol..

[19] Cesarino, I. *et al*. Building the wall: recent advances in understanding lignin metabolism in grasses. *Acta Physiol. Plant*. **38** (2016).

[20] Eloy N (2017). Silencing chalcone synthase impedes the incorporation of tricin in lignin and increases lignin content. Plant Physiol..

[21] Vanholme R (2010). Engineering traditional monolignols out of lignin by concomitant up-regulation of F5H1 and down-regulation of COMT in *Arabidopsis*. Plant J.

[22] Vanholme R (2012). A systems biology view of responses to lignin biosynthesis perturbations in *Arabidopsis*. Plant Cell.

[23] Li Y, Kajita S, Kawai S, Katayama Y, Morohoshi N (2003). Down-regulation of an anionic peroxidase in transgenic aspen and its effect on lignin characteristics. J. Plant Res.

[24] Ralph J (2006). Effects of coumarate 3-hydroxylase down-regulation on lignin structure. J. Biol. Chem..

[25] Vanholme R (2013). Caffeoyl shikimate esterase (CSE) is an enzyme in the lignin biosynthetic pathway in *Arabidopsis*. Science (80-.).

[26] Ha CM (2016). An essential role of caffeoyl shikimate esterase in *monolignol biosynthesis* in Medicago truncatula. Plant J..

[27] Saleme, M. *et al*. Silencing caffeoyl shikimate esterase affects lignification and improves saccharification. *Plant Physiol*. pp-00920 (2017). 10.1104/pp.17.00920 PMC5664470 28878037

[28] Zhou J, Lee C, Zhong R, Ye Z-H (2009). MYB58 and MYB63 are transcriptional activators of the lignin biosynthetic pathway during secondary cell wall formation in *Arabidopsis*. Plant Cell.

[29] Fu C (2011). Genetic manipulation of lignin reduces recalcitrance and improves ethanol production from switchgrass. Proc. Natl. Acad. Sci..

[30] Jung HG, Engels FM (2002). Alfalfa stem tissues. Crop Sci.

[31] Cesarino I, Araújo P, Mayer JLS, Leme AFP, Mazzafera P (2012). Enzymatic activity and proteomic profile of class III peroxidases during sugarcane stem development. Plant Physiol. Biochem..

[32] Cesarino I (2013). Expression of SofLAC, a new laccase in sugarcane, restores lignin content but not S:G ratio of *Arabidopsis* lac17 mutant. J. Exp. Bot..

[33] Bottcher A (2013). Lignification in sugarcane: biochemical characterization, gene discovery, and expression analysis in two genotypes contrasting for lignin content. Plant Physiol..

[34] dos Santos AB (2015). Water stress alters lignin content and related gene expression in two sugarcane genotypes. J. Agric. Food Chem..

[35] dos Santos AB (2015). Lignin biosynthesis in sugarcane is affected by low temperature. Environ. Exp. Bot..

[36] Ferreira SS (2016). Co-expression network analysis reveals transcription factors associated to cell wall biosynthesis in sugarcane. Plant Mol. Biol..

[37] Jung JH, Fouad WM, Vermerris W, Gallo M, Altpeter F (2012). RNAi suppression of lignin biosynthesis in sugarcane reduces recalcitrance for biofuel production from lignocellulosic biomass. Plant Biotechnol. J..

[38] Bewg WP, Poovaiah C, Lan W, Ralph J, Coleman HD (2016). RNAi downregulation of three key lignin genes in sugarcane improves glucose release without reduction in sugar production. Biotechnol. Biofuels.

[39] Jung JH, Altpeter F (2016). TALEN mediated targeted mutagenesis of the caffeic acid O-methyltransferase in highly polyploid sugarcane improves cell wall composition for production of bioethanol. Plant Mol. Biol..

[40] Cheavegatti-Gianotto A (2011). Sugarcane (*Saccharum officinarum*): A Reference Study for the Regulation of Genetically Modified Cultivars in Brazil. Trop. Plant Biol..

[41] Ming, R. *et al*. Sugarcane Improvement through Breeding and Biotechnology. In *Plant Breeding Reviews* 15–118 (2006).

[42] Tew, T. & Cobill, R. Genetic Improvement of Sugarcane (*Saccharum* spp.) as an Energy Crop. In *Genetic Improvement of Bioenergy Crops SE - 9* (ed. Vermerris, W.) 273–294 (Springer New York, 2008).

[43] Moore PH (1995). Temporal and spatial regulation of sucrose accumulation in the sugarcane stem. Funct. Plant Biol..

[44] Jung H-JG, Samac DA, Sarath G (2012). Modifying crops to increase cell wall digestibility. Plant Sci..

[45] Carroll A, Somerville C (2009). Cellulosic Biofuels. Annu. Rev. Plant Biol..

[46] Rezende CA (2011). Chemical and morphological characterization of sugarcane bagasse submitted to a delignification process for enhanced enzymatic digestibility. Biotechnol. Biofuels.

[47] Szczerbowski D, Pitarelo AP, Zandoná Filho A, Ramos LP (2014). Sugarcane biomass for biorefineries: comparative composition of carbohydrate and non-carbohydrate components of bagasse and straw. Carbohydr. Polym..

[48] Carvalho-Netto OV (2014). The potential of the energy cane as the main biomass crop for the cellulosic industry. Chem. Biol. Technol. Agric.

[49] Van Heiningen A (2006). Converting a kraft pulp mill into an integrated forest biorefinery. Pulp Pap. Canada.

[50] Price S (1963). Cytogenetics of modern sugar canes. Econ. Bot..

[51] Daniels, J. & Roach, B. T. Taxonomy and evolution In *Sugarcane Improv. through breeding* (ed. Heinz, D. J.) 7–84, (Elsevier Science, 1987).

[52] Burnquist WL, Sorrells ME, Tanksley S (1992). Characterization of genetic variability in Saccharum germplasm by means of restriction fragment length polymorphism (RFLP) analysis. Proc. Int. Soc. Sugar Cane Technol.

[53] Vijayan Nair N, Nair S, Sreenivasan TV, Mohan M (1999). Analysis of genetic diversity and phylogeny in Saccharum and related genera using RAPD markers. Genet. Resour. Crop Evol.

[54] Stevenson, G. C. *Genetics and breeding of sugar cane*. (Longman, 1965).

[55] D’Hont A, Paulet F, Glaszmann JC (2002). Oligoclonal interspecific origin of ‘North Indian’ and ‘Chinese’ sugarcanes. Chromosom. Res.

[56] Cheng K, Sorek H, Zimmermann H, Wemmer DE, Pauly M (2013). Solution-state 2D NMR spectroscopy of plant cell walls enabled by a dimethylsulfoxide-d 6/1-ethyl-3-methylimidazolium acetate solvent. Anal. Chem..

[57] Chong, S.-L. *et al*. O-Acetylation of glucuronoxylan in *Arabidopsis thaliana* wild type and its change in xylan biosynthesis mutants. *Glycobiology* cwu017 (2014). 10.1093/glycob/cwu017 24637390

[58] Inman-Bamber G, Jackson P, Bonnett G, Morgan T (2011). Have we reached peak CCS?. Int. Sugar J..

[59] Zhou, M., Kimbeng, C. A., Eggleston, G., Veremis, J. C. & Gravois, K. A. Prospects of breeding for low starch content in sugarcane. In *Proceedings of the International Society of Sugar Cane Technologists* **26**, 724–729 (2007).

[60] Rancour DM, Marita JM, Hatfield RD (2012). Cell wall composition throughout development for the model grass *Brachypodium distachyon*. Front. Plant Sci..

[61] Bekker, J. P. I. *Genetic manipulation of the cell wall composition of sugarcane*. (Stellenbosch: University of Stellenbosch, 2007).

[62] Waclawovsky AJ, Sato PM, Lembke CG, Moore PH, Souza GM (2010). Sugarcane for bioenergy production: an assessment of yield and regulation of sucrose content. Plant Biotechnol. J.

[63] Sarath G, Baird LM, Vogel KP, Mitchell RB (2007). Internode structure and cell wall composition in maturing tillers of switchgrass (*Panicum virgatum. L*). Bioresour. Technol..

[64] Butkutė B, Lemežienė N, Cesevičienė J, Liatukas Ž, Dabkevičienė G (2013). Carbohydrate and lignin partitioning in switchgrass *Panicum virgatum* biomass as a bioenergy feedstock. Zemdirbyste-Agriculture.

[65] Matos DA, Whitney IP, Harrington MJ, Hazen SP (2013). Cell walls and the developmental anatomy of the *Brachypodium distachyon* stem internode. PLoS One.

[66] Jung HG, Casler MD (2006). Maize Stem Tissues: Cell Wall Concentration and Composition during Development. Crop Sci.

[67] Zhang Q (2014). Spatial gradients in cell wall composition and transcriptional profiles along elongating maize internodes. BMC Plant Biol.

[68] Slewinski TL (2012). Non-structural carbohydrate partitioning in grass stems: a target to increase yield stability, stress tolerance, and biofuel production. J. Exp. Bot..

[69] Pauly M, Keegstra K (2010). Plant cell wall polymers as precursors for biofuels. Curr. Opin. Plant Biol..

[70] Hatfield R, Fukushima RS (2005). Can lignin be accurately measured?. Crop Sci.

[71] Ralph J (1997). Abnormal lignin in a loblolly pine mutant. Science.

[72] Grabber JH, Hatfield RD, Lu F, Ralph J (2008). Coniferyl ferulate incorporation into lignin enhances the alkaline delignification and enzymatic degradation of cell walls. Biomacromolecules.

[73] Kishimoto T (2009). Influence of syringyl to guaiacyl ratio on the structure of natural and synthetic lignins. J. Agric. Food Chem..

[74] Morreel K (2010). Mass spectrometry-based sequencing of lignin oligomers. Plant Physiol..

[75] Santos RB, Hart P, Jameel H, Chang H (2013). Wood based lignin reactions important to the biorefinery and pulp and paper industries. BioResources.

[76] Barrière Y (2007). Genetics and genomics of lignification in grass cell walls based on maize as model species. Genes Genomes Genomics.

[77] Yasuda S, Fukushima K, Kakehi A (2001). Formation and chemical structures of acid-soluble lignin I: sulfuric acid treatment time and acid-soluble lignin content of hardwood. J. wood Sci..

[78] Chen L (2002). Lignin deposition and associated changes in anatomy, enzyme activity, gene expression, and ruminal degradability in stems of tall fescue at different developmental stages. J. Agric. Food Chem..

[79] Shen H (2009). Developmental control of lignification in stems of lowland switchgrass variety Alamo and the effects on saccharification efficiency. BioEnergy Res.

[80] Ferrer J-L, Austin MB, Stewart C, Noel JP (2008). Structure and function of enzymes involved in the biosynthesis of phenylpropanoids. Plant Physiol. Biochem..

[81] Wagner A (2015). Syringyl lignin production in conifers: Proof of concept in a Pine tracheary element system. Proc. Natl. Acad. Sci..

[82] Helle S, Cameron D, Lam J, White B, Duff S (2003). Effect of inhibitory compounds found in biomass hydrolysates on growth and xylose fermentation by a genetically engineered strain of *S. cerevisiae*. Enzyme Microb. Technol..

[83] Xiong G, Cheng K, Pauly M (2013). Xylan O-acetylation impacts xylem development and enzymatic recalcitrance as indicated by the *Arabidopsis* mutant tbl29. Mol Plant.

[84] Gille S (2011). O-acetylation of *Arabidopsis* hemicellulose xyloglucan requires AXY4 or AXY4L, proteins with a TBL and DUF231 domain. Plant Cell.

[85] Gille, S. & Pauly, M. O-acetylation of plant cell wall polysaccharides. *Front. Plant Sci*. **3** (2012). 10.3389/fpls.2012.00012 PMC3355586 22639638

[86] Pawar PM-A, Koutaniemi S, Tenkanen M, Mellerowicz EJ (2013). Acetylation of woody lignocellulose: significance and regulation. Front Plant Sci.

[87] Klein-Marcuschamer D, Oleskowicz-Popiel P, Simmons BA, Blanch HW (2010). Technoeconomic analysis of biofuels: A wiki-based platform for lignocellulosic biorefineries. biomass and bioenergy.

[88] Scheller HV, Ulvskov P (2010). Hemicelluloses. Plant Biol..

[89] Piperidis G, Piperidis N, D’Hont A (2010). Molecular cytogenetic investigation of chromosome composition and transmission in sugarcane. Mol. Genet. Genomics.

[90] Rawal, H. C., Singh, N. K. & Sharma, T. R. Conservation, divergence, and genome-wide distribution of PAL and POX A gene families in plants. *Int. J. Genomics* **2013** (2013). 10.1155/2013/678969 PMC3647544 23671845

[91] Zhong R (2011). Transcriptional activation of secondary wall biosynthesis by rice and maize NAC and MYB transcription factors. Plant Cell Physiol.

[92] Poovaiah CR, Nageswara-Rao M, Soneji JR, Baxter HL, Stewart CN (2014). Altered lignin biosynthesis using biotechnology to improve lignocellulosic biofuel feedstocks. Plant Biotechnol. J..

[93] Poovaiah CR, Bewg WP, Lan W, Ralph J, Coleman HD (2016). Sugarcane transgenics expressing MYB transcription factors show improved glucose release. Biotechnol. Biofuels.

[94] Handakumbura PP, Hazen SP (2007). Transcriptional regulation of grass secondary cell wall biosynthesis: playing catch-up with Arabidopsis thaliana. Front. Plant Sci..

[95] Zhao Q, Dixon RA (2011). Transcriptional networks for lignin biosynthesis: more complex than we thought?. Trends Plant Sci.

[96] Gray J, Caparrós-Ruiz D, Grotewold E (2012). Grass phenylpropanoids: regulate before using!. Plant Sci..

[97] Valdivia, E. R. *et al*. Regulation of secondary wall synthesis and cell death by NAC transcription factors in the monocot *Brachypodium distachyon*. *J. Exp. Bot*. ers394 (2013). 10.1093/jxb/ers394 PMC3598421 23386682

[98] Zhong R (2015). Functional Characterization of NAC and MYB Transcription Factors Involved in Regulation of Biomass Production in Switchgrass (*Panicum virgatum*). PLoS One.

[99] Johansen, D. A. *Plant microtechnique*. (McGraw-Hill Publishing Company, Ltd., London, 1940).

[100] Mäule, C. *Das verhalten verholzter membranen gegen kaliumpermanganat, eine holzreaktion neuer art*. (A. Zimmer’s Verlag (Ernst Mohrmann), 1901).

[101] Berlyn, G. P. & Miksche, J. P. *Botanical Microtechnique and Cytochemistry*. (Iowa State University Press, 1976).

[102] DuBois M, Gilles KA, Hamilton JK, Rebers PA, Smith F (1956). Colorimetric method for determination of sugars and related substances. Anal. Chem..

[103] van Handel E (1968). Direct microdetermination of sucrose. Anal. Biochem..

[104] Nelson N (1944). A photometric adaptation of the somogyi method for the determination of glucose. J. Biol. Chem..

[105] Amaral LD, Gaspar M, Costa PMF, Aidar MPM, Buckeridge MS (2007). Novo método enzimático rápido e sensível de extração e dosagem de amido em materiais vegetais. Hoehnea.

[106] Swain T, Hillis WE (1959). The phenolic constituents of Prunus domestica. I.—The quantitative analysis of phenolic constituents. J. Sci. Food Agric..

[107] TAPPI. TAPPI Useful Method UM 250: Acid-soluble lignin in wood and plants. In *TAPPI Useful Methods* (ed. TAPPI) (1985).

[108] Mokochinski JB (2015). A simple protocol to determine lignin S/G ratio in plants by UHPLC-MS. Anal. Bioanal. Chem..

[109] Brown, L. & Torget, R. *NREL analytical procedure: LAP009 enzymatic saccharification of lignocellulosic biomass hydrolysis. Golden, CO: National Renewable Energy Laboratory* (1996).

[110] Hall TA (1999). BioEdit: a user-friendly biological sequence alignment editor and analysis program for Windows 95/98/NT. Nucleic Acids Symp. Ser..

[111] Rozen, S. & Skaletsky, H. Primer3 on the WWW for general users and for biologist programmers. *Bioinforma. methods Protoc*. 365–386 (1999). 10.1385/1-59259-192-2:365 10547847

[112] Llerena JPP, Araujo P, Mazzafera P (2018). Optimization of RT-PCR reactions in studies with genes of lignin biosynthetic route in Saccharum spontaneum. An. Acad. Bras. Cienc..

[113] Thompson, J. D., Gibson, T., Higgins, D. G. Multiple sequence alignment using ClustalW and ClustalX. *Curr. Protoc. Bioinforma*. 2–3 (2002). 10.1002/0471250953.bi0203s00 18792934

[114] Livak KJ, Schmittgen TD (2001). Analysis of relative gene expression data using real-time quantitative PCR and the 2(-Delta Delta C(T)) Method. Methods.

[115] Brown, L. C. & Mac Berthouex, P. *Statistics for environmental engineers*. (CRC press, 2002).

