# Supplementary material for: Deposition of lignin in four species of Saccharum
Source: Sci Rep. 2019 Apr 10;9:5877. doi: 10.1038/s41598-019-42350-3 (PMC6458172; doi:10.1038/s41598-019-42350-3)
Supplement: Supplementary file 1 — Supplementary information 1 [file 41598_2019_42350_MOESM1_ESM.docx]

**Deposition of lignin in four species of *Saccharum***

Juan Pablo Portilla Llerena^1^, Raquel Figueiredo^1^, Michael dos Santos Brito^3^, Eduardo Kiyota^1^, Juliana Lischka Sampaio Mayer^1^, Pedro Araujo^2^, Murali Dama^4^, Markus Pauly^4^,Flavia Camila Schimpl^1^, Paulo Mazzafera^1,5,^*

^1^Departamento de Biologia Vegetal, Instituto de Biologia, CP 6109, Universidade Estadual de Campinas, Campinas, SP 13083-970, Brazil

^2^Departamento de Genética Evolução e Bioagentes, Instituto de Biologia, CP 6109, Universidade Estadual de Campinas, Campinas, SP 13083-970, Brazil

^3^Instituto de Ciência e Tecnologia, Universidade Federal de São Paulo, Campus São José dos Campos, São José dos Campos, SP 12231-280, Brazil

^4^Heinrich Heine University, Institute for Plant Cell Biology and Biotechnology, D-40225 Düsseldorf, Germany

^5^Departamento de Produção Vegetal, Escola Superior de Agricultura Luiz de Queiroz, Universidade de São Paulo, Piracicaba, SP, Brazil

***For correspondence**. Paulo Mazzafera, Prof. Dr.

E-mail [pmazza@unicamp.br](mailto:pmazza@unicamp.br)

Lab. Fisiologia Molecular de Plantas/IB/Unicamp

Lab. Produção Vegetal/ESALQ-USP

Office: +55 19 3429 4148

Cel: +55 19 99694 0718

**Supplementary Table S1**. Sequences of the primer pairs used in the amplification of lignin biosynthetic route genes in *Saccharum* species

| **Gene** | **Forward 5´-->3´** | **Reverse 5´-->3´** | **Amplicon (bp) and %GC** |
| --- | --- | --- | --- |
| ***4CL*** | AGCTKCCGGACATCGASATC  GCCGCTGTTCCACATCTACT  CCCTCTCGCATGTACAGGTT | CTTGTAGAAMACCACCTCCTTKG  -------------------------------------------  ------------------------------------------- | 1340 (65%)  550  550 |
| ***CAD*** | TACTGYGGSATATGCCAC | CGAYGTCGATGACGAAGC | 973 (65%) |
| ***C3H*** | AACCTGCGCCAGATCAAG  ACCTTTCTATGGTGGCCTTC | GTGCCCATGAASGTGACG  ------------------------------------------- | 1343 (58%)  550 |
| ***C4H*** | GTTCGGCGACATCTTCCTC | CTTCTCCGTGGTGTCGATCT | 1281 (63%) |
| ***CCoAOMT*** | CTCAAGAGCGAAGMCCTSTAC | GAGCTGGCAGAYCTCGAC | 649 (63%) |
| ***CCR*** | GSCTCGTGGSTCGTCAAG | GGTTSGAGAACTTGTACGGCT | 823 (68%) |
| ***COMT*** | GTCCATCCTGCCCATGAC | CAGTCGTGGAGGATCCACTT | 756 (66%) |
| ***HCT*** | GGGGTCGGAGATGGTGTA  ATCGACTACTTCGGCGACTTC | CGGAACTTCTCCATGTGCTC  ------------------------------------------- | 1317 (72%)  550 |
| ***F5H*** | CGWTGATGGACCAGCTGAC | TGCTCGTCGATGATCTTGTC | 625 (68%) |

**Supplementary Table S2.** Gene names primers, and size of amplicons used for RT-qPCR analysis in the biosynthetic pathway of lignin in *Saccharum* species

| **Gen** | **Primer Pairs** | **Amplicom ( bp)** |
| --- | --- | --- |
| ***C4H*** | F 5´-CGTTCCTCCGTGGGTATCT-3´  R 5´-CATCACCTTCTTGCGTTCCT-3´ | **97** |
| ***4CL*** | F- 5´-AGCCGTTCCAGGTCAAGTC-3´  R- 5´-ACTCGGGGTCGTTCAGGTA-3´ | **161** |
| ***HCT*** | F- 5´-TCAGACGACACCGCCTTC-3´  R-5´-GTCCGCCCACGAGTTGAT-3´ | **137** |
| ***C3H*** | F-5´-TAGTGCGGAACCACCTTTCT-3´  R-5´-TTCGTCAATGTCACCGTTTG-3´ | **94** |
| ***CCoAOMT A*** | F- 5´-CTCGTGACCGACAAGCAC-3´  R- 5´-AGGGAGTAGCCCGTGAACA-3´ | **130** |
| ***CCoAOMT B*** | F- 5´-ACGCCGACAAGGACAACTAC-3´  R-5´-GCGGTAGAAGCGGATGTACT-3´ | **151** |
| ***CCR*** | F-5´-ACTGTCAAGGGAACCGTCAG-3´  R-5´-CAGATGGCGTCGTAGTCCAG-3´ | **121** |
| ***F5H*** | F-5´-AGACGCAGGACGGAGTGTT-3´  R-5´-AAGAGCTTCATCACGCACAG-3´ | **138** |
| ***COMT*** | F-5´-GAGGACAAGGACGGCAAGTA-3´  R-5´-ACCGCGTCCTTGAGGTAGTA-3´ | **154** |
| ***CAD A*** | F-5´-TCAAGAACGACTGGGGAAAC-3´  R-5´-GCAGGAGCCGACGAAGTA-3´ | **140** |
| ***CAD B*** | F-5´-ATCAGCTCGTCGTCCAAGAA-3´  R-5´-CGATGATGTAGTCCAGCGAGT-3´ | **120** |
| ***GAPDH*** | F-5´-TTGGTTTCCACTGACTTCGTT-3´  R-5´-CTGTAGCCCCACTCGTTGT-3´ |  |

**Supplementary Table S3**. Unigenes list of the biosynthetic pathway of lignin identified to the species of *Saccharum*, relative abundance of sequenced reads and orthologs in sugarcane. Sb= *S. barberi*, So= *S. officinarum*, Sr= *S. robustum*, Ss= *S. spontaneum.*

| **Ortholog in Sugarcane /SAS** | **Identified Gene Name** | **Relative abundance of sequenced reads** |
| --- | --- | --- |
| ***ShC4H1* SCCCCL4009H01.g** | ***SbC4H***  ***SoC4H***  ***SrC4H***  ***SsC4H*** | **100%** |
| ***Sh4CL1* SCCCCL3002A03.g** | ***Sb4CL A*** | **80%** |
|  | ***So4CL***  ***Sr 4CL***  ***Ss4CL*** | **100%** |
| ***Sh4CL3* SCMCRT2102F02.g** | ***Sb4CL B*** | **20%** |
| ***ShHCT1* SCCCCL4009E02.g** | ***SbHCT***  ***SoHCT***  ***SrHCT***  ***SsHCT*** | **100%** |
| ***ShC3H1* SCVPCL6041E07.g** | ***SbC3H***  ***SoC3H***  ***SrC3H***  ***SsC3H*** | **100%** |
| ***ShCCoAOMT2***  **SCJLRT2050C09.g** | ***SbCCoAOMT A***  ***SoCCoAOMT A***  ***SrCCoAOMT A***  ***SsCCoAOMT A*** | **80.00%**  **30.76%**  **26.60%**  **50.00%** |
| ***ShCCoAOMT1* SCCCLR1069B09.g** | ***SbCCoAOMT B***  ***SoCCoAOMT B***  ***SrCCoAOMT B***  ***SsCCoAOMT B*** | **20.00%**  **69.23%**  **73.30%**  **50.00%** |
| ***ShCCR1***  **SCCCRZ2C01A04.g** | ***SbCCR***  ***SoCCR***  ***SrCCR***  ***SsCCR*** | **100%** |
| ***ShF5H1***  **SCJLRT1022E04.g** | ***SbF5H***  ***SoF5H***  ***SrF5H***  ***SsF5H*** | **100%** |
| ***ShCOMT1***  **SCJLRT1023B09.g** | ***SbCOMT***  ***SoCOMT***  ***SrCOMT***  ***SsCOMT*** | **100%** |
| ***ShCAD8* SCEQLR1029E05.g** | ***SbCAD A***  ***SoCAD A***  ***SrCAD A***  ***SsCAD A*** | **86.60%**  **73.30%**  **46.60%**  **80.00%** |
| ***ShCAD2* SCCCLB1001F10.g** | ***SbCAD B***  ***SoCAD B***  ***SrCAD B***  ***SsCAD B*** | **13.30%**  **26.60%**  **26.60%**  **20.00%** |
| ***ShCAD7* SCEPRZ1011A02.g** | ***SrCAD C*** | **26.60%** |

**Supplementary Figures S1-S9**. Phylogenetic analyzes of amino acid sequences of the enzymes of the monolignol biosynthetic pathway, obtained from the NCBI, GeneBank, Phytozome database and homologues identified in this study for *Saccharum* species. The blue annotations show the genes (and their respective SASs) identified by Bottcher et al.^1^

**Supplementary Figure S1 - C4H**


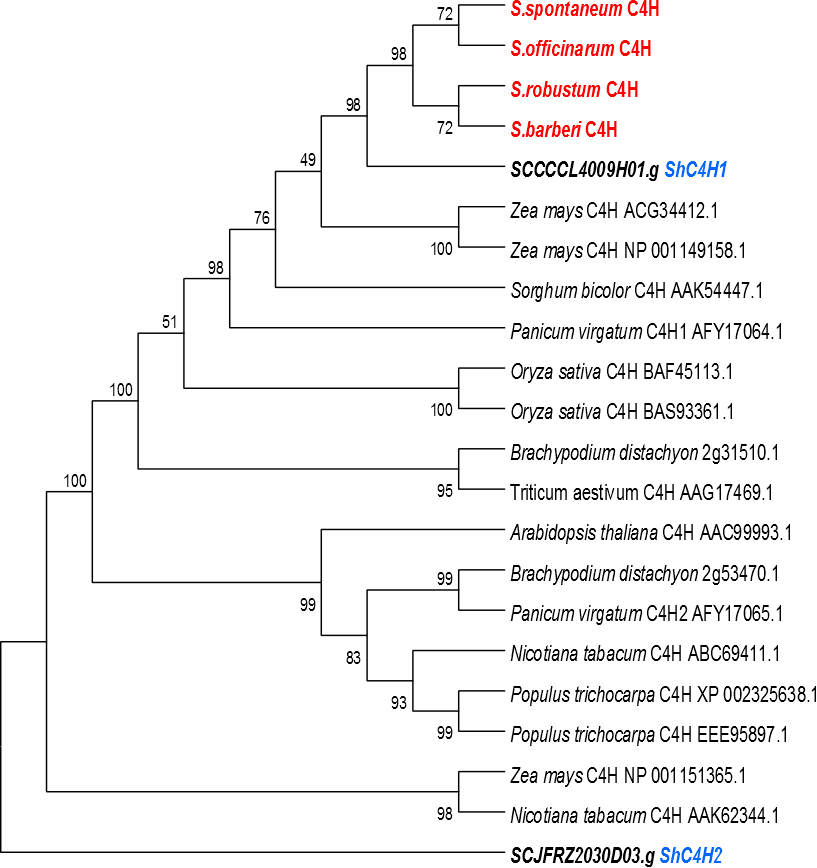


I

II

III

**Supplementary Figure 2S – 4CL**


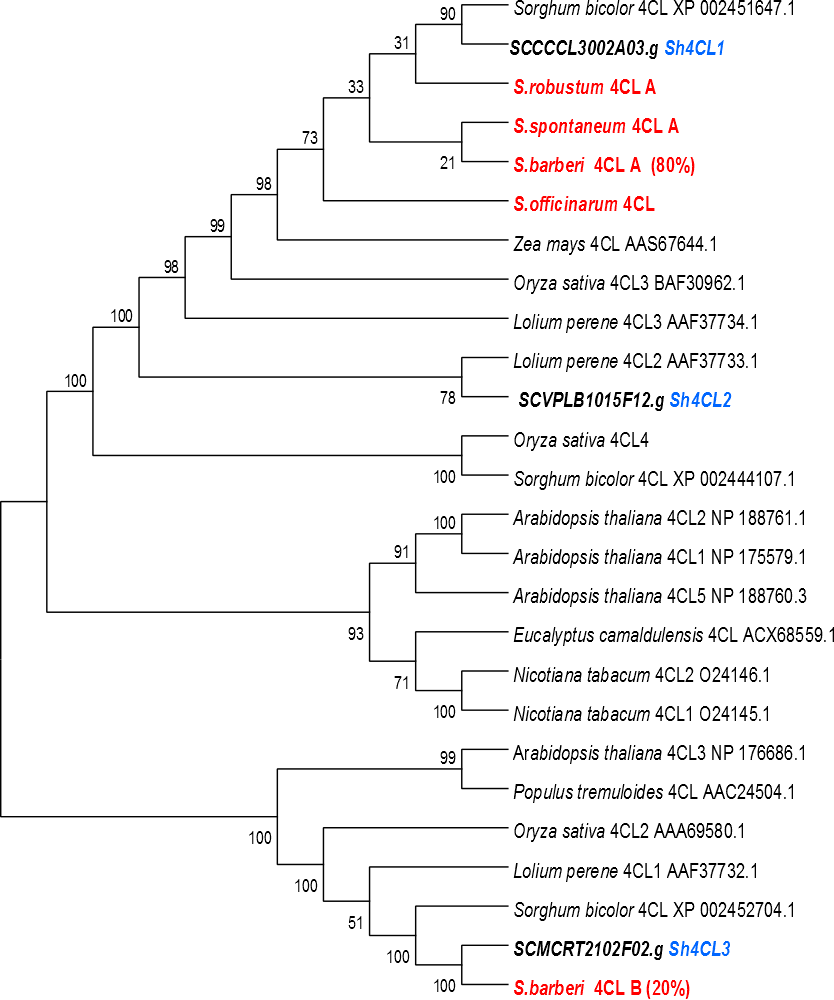


I

II

III

IV

**Supplementary Figure S3 – HCT**


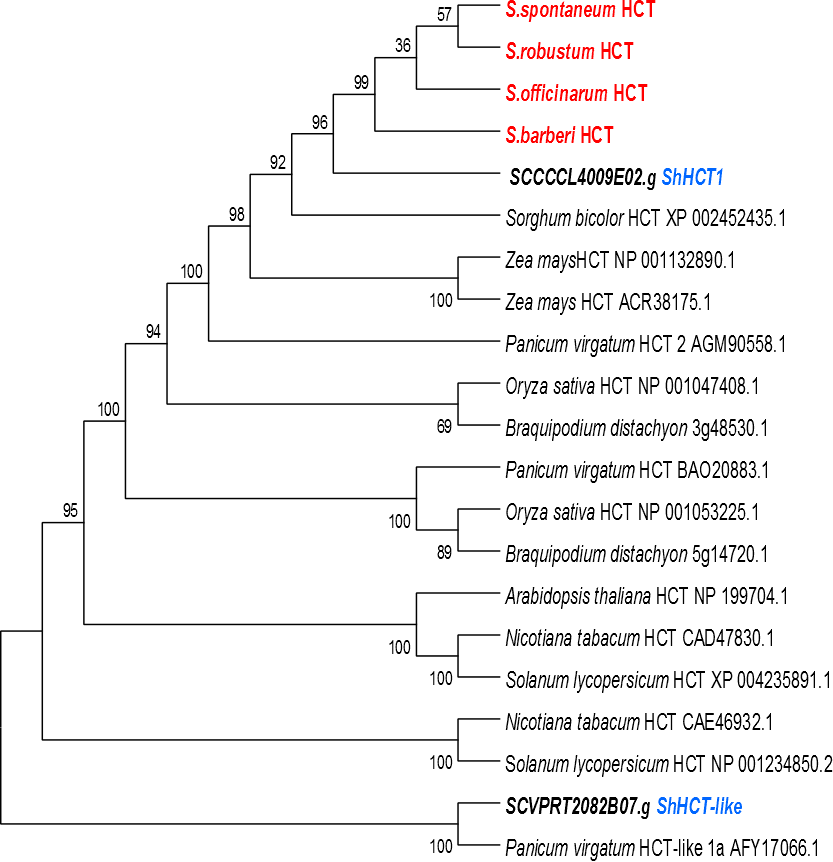


I

II

III

IV

**Supplementary Figure S4 – C3H**


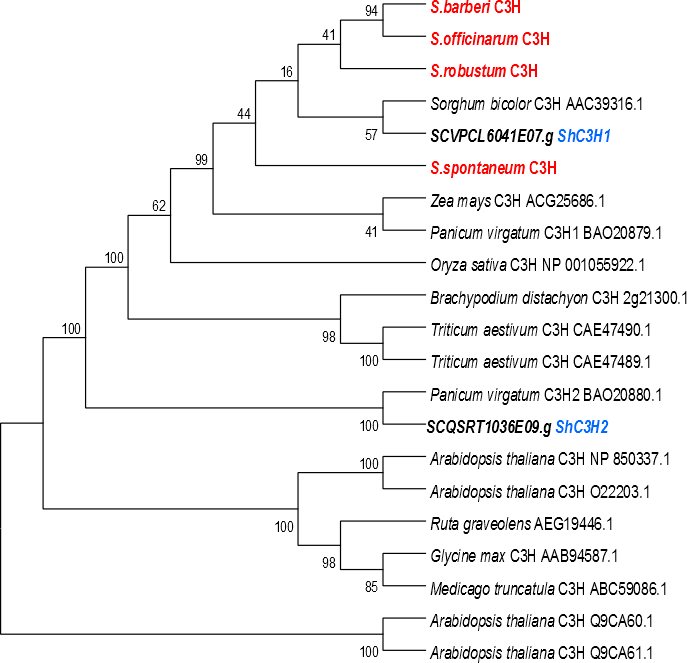


I

II

III

**Supplementary Figure S5 – CCoAOMT**


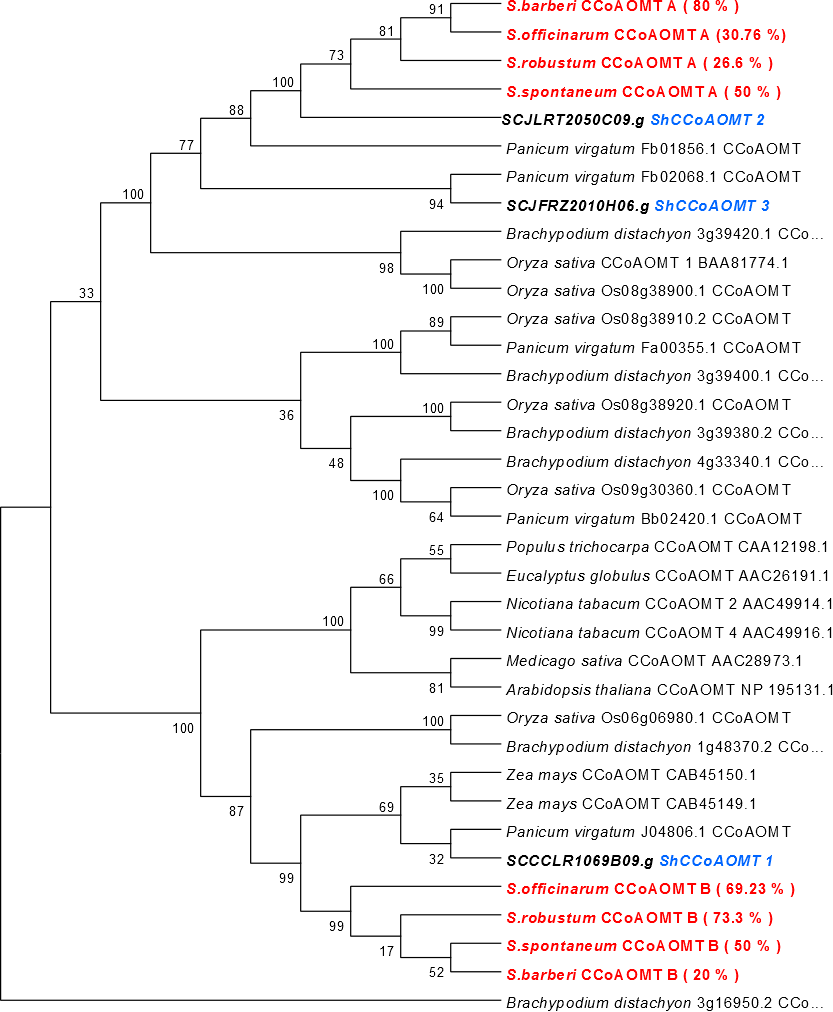


I

II

III

IV

**Supplementary Figure S6 – CCR**

I

II

III

IV

**Supplementary Figure S7 – F5H**


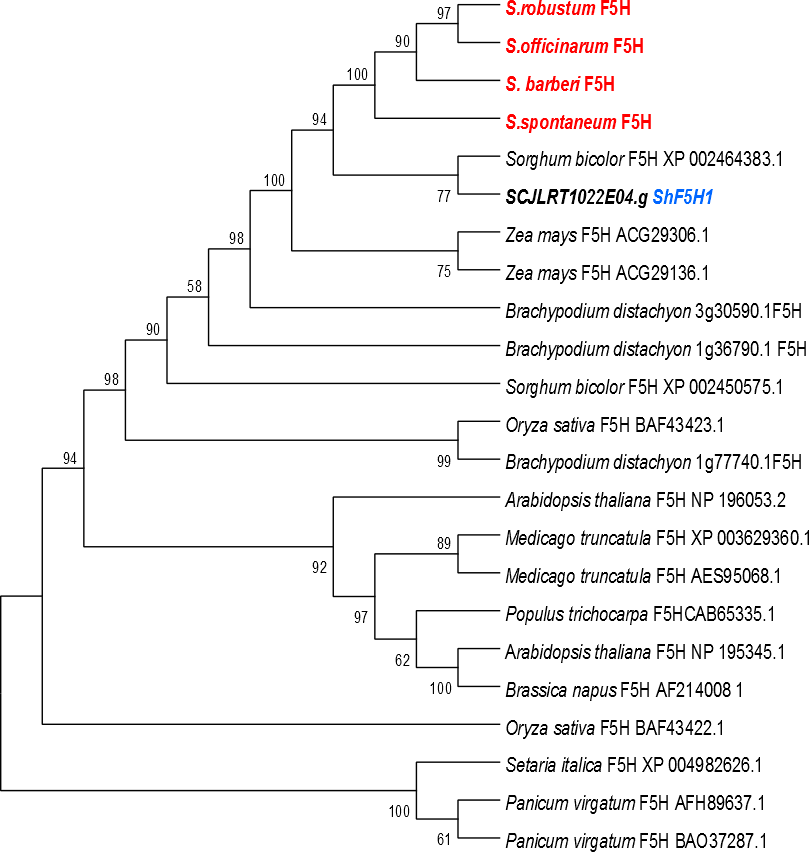


I

II

III

**Supplementary Figure S8 – COMT**


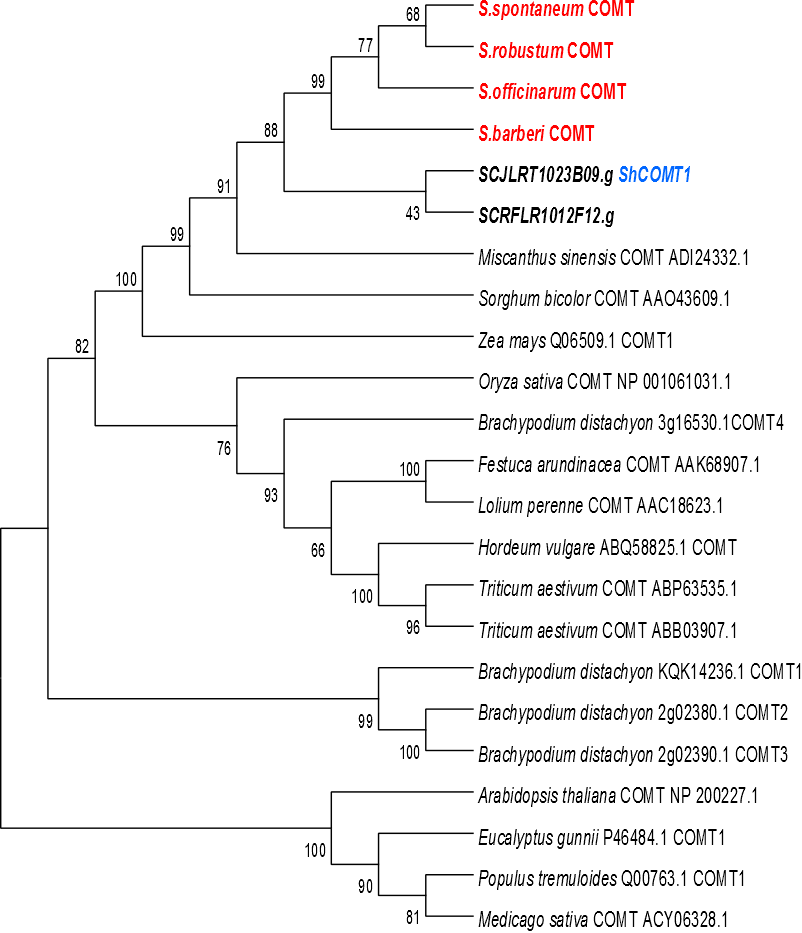


I

II

III

IV

**Supplementary Figure S9 – CAD**


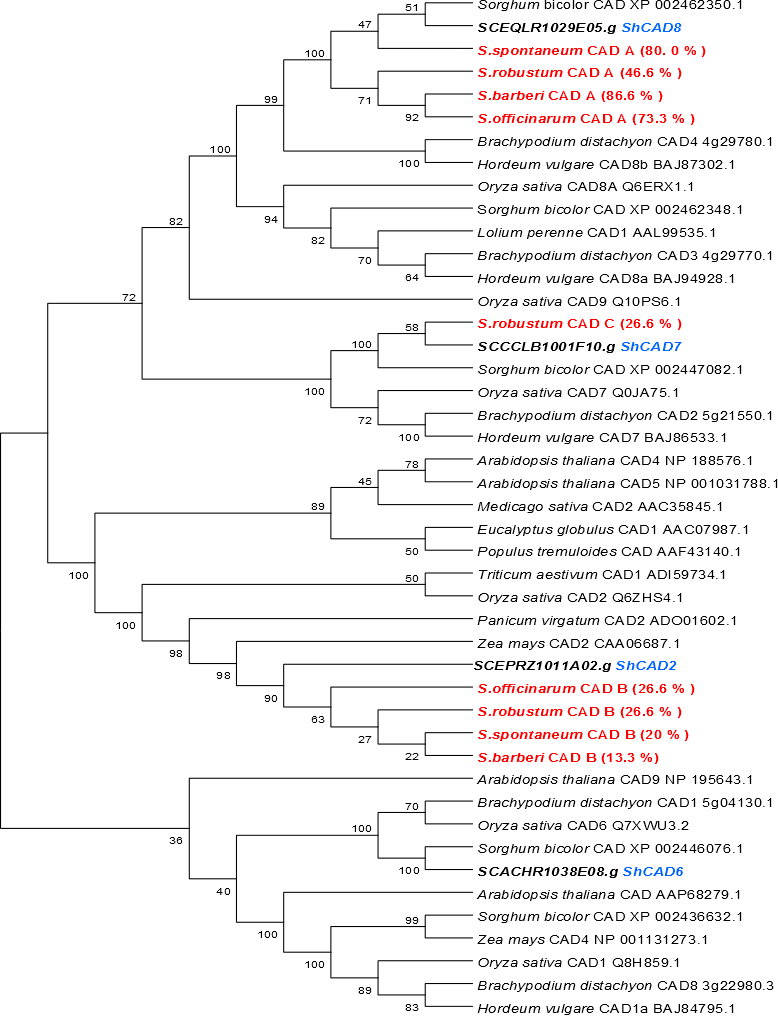


I

II

III

**Supplementary Figures S10-S18**. Alignment of amino acid sequences of the enzymes of the bionosynthetic pathway of the monolignes in the species *Saccharum spontaneum* (Ss), *Saccharum barberi* (Sba), *Saccharum officinarum* (So), *Saccharum robustum* (Sr), *Miscanthus sinensis* (Ms), *Lolium perenne* (Lp), *Panicum virgatum* (Pv) and *Sorghum bicolor* (Sb).

**Supplementary Figure S10-C4H**

*Sh*C4H2 SCJFRZ2030D03.g 1 ------------------------------------------------------------
Zm C4H 1 MDLALLEKALLGLFAAAVVAIAVAKLSGKRYRLPPGPPGAPVVGNWLQVGDDLNHRNLMA
Zm C4H 1 MDLALLEKALLGLFAAAVVAIAVAKLSGKRYRLPPGPPGAPVVGNWLQVGDDLNHRNLMA *Sh*C4H1 SCCCCL4009H01.g 1 MDLVLLEKALLGLFAAAVVAIAVAKLTGKRYRLPPGPPGAPVVGNWLQVGDDLNHRNLMA
*Ss C4H 1 -----------------------------------------------LFGDIFL--RSTP
*So C4H 1 ------------------------------------------------------------
*Sr C4H 1 ------------------------------------------------------------
*Sba C4H 1 ------------------------------------------------------------

*Sh*C4H2 SCJFRZ2030D03.g 1 ------------------------------------------------------------
Zm C4H 61 MAKRFGDIFLLRMGVRNLVVVSTPELAKEVLHTQGVEFGSRTRNVVFDIFTGKGQDMVFT
Zm C4H 61 MAKRFGDIFLLRMGVRNLVVVSTPELAKEVLHTQGVEFGSRTRNVVFDIFTGKGQDMVFT
*Sh*C4H1 SCCCCL4009H01.g 61 LAKRFGDIFLLRMGVRNLVVVSTPELAKEVLHTQGVEFGSRTRNVVFDIFTGKGQDMVFT
*Ss C4H 12 RRRLFGDIFLLRMGVRNLVVVSTPELAKEVLHTQGVEFGSRTRNVVFDIFTGKGQDMVFT
*So C4H 1 ---LFGDIFLLRMGVRNLVVVSTPELAKEVLHTQGVEFGSRTRNVVFDIFTGKGQDMVFT
*Sr C4H 1 --HLFGDIFLLRMGVRNLVVVSTPELAKEVLHTQGVEFGSRTRNVVFDIFTGKGQDMVFT
*Sba C4H 1 ---LFGDIFLLRMGVRNLVVVSTPELAKEVLHTQGVEFGSRTRNVVFDIFTGKGQDMVFT


*Sh*C4H2 SCJFRZ2030D03.g 1 ------------------------------------------------------------
Zm C4H 121 VYGDHWRKMRRIMTVPFFTNKVVAQNRAGWEEEARLVVEDVRKDPEAAAGGVVLRRRLQL
Zm C4H 121 VYGDHWRKMRRIMTVPFFTNKVVAQNRAGWEEEARLVVEDVRKDPEAAAGGVVLRRRLQL
*Sh*C4H1 SCCCCL4009H01.g 121 VYGDHWRKMRRIMTVPFFTNKVVAQNRAGWEEEARLVVEDVRRDPRAAAEGVVIRKRLQL

*Ss C4H 72 VYGDHWRKMRRIMTVPFFTNKVVAQNRAGWEEEARLVVEDVRRDPRAAAEGVVIRKRLQL
*So C4H 58 VYGDHWRKMRRIMTVPFFTNKVVAQNRAGWEEEARLVVEDVRRDPRAAAEGVVIRKRLQL
*Sr C4H 59 VYGDHWRKMRRIMTVPFFTNKVVAQNRAGWEEEARLVVEDVRRDPRAAAEGVVIRKRLQL
*Sba C4H 58 VYGDHWRKMRRIMTVPFFTNKVVAQNRAGWEEEARLVVEDVRRDPRAAAEGVVIRKRLQL


*Sh*C4H2 SCJFRZ2030D03.g 1 -MVKRMVC-AKAQKVNKRWDELLDKLIDDHANATRSESQHVDEESDFIDL----------
Zm C4H 181 MMYNDMFRIMFDRRFDSEHDPLFNKLKALNAERSRLSQSFEYNYGDFIPVLRPFLRGYLN
Zm C4H 181 MMYNDMFRIMFDRRFDSEHDPLFNKLKALNAERSRLSQSFEYNYGDFIPVLRPFLRGYLN
*Sh*C4H1 SCCCCL4009H01.g 181 MMYNDMFRIMFDRRFESEEDPLFNKLKALNAERSRLSQSFEYNYGDFIPVLRPFLRGYLN
*Ss C4H 132 MMYNDMFRIMFDRRFESEEDPLFNKLKALNAERSRLSQSFEYNYGDFIPVLRPFLRGYLN
*So C4H 118 MMYNDMFRIMFDRRFESEEDPLFNKLKALNAERSRLSQSFEYNYGDFIPVLRPFLRGYLN
*Sr C4H 119 MMYNDMFRIMFDRRFESEEDPLFNKLKALNAERSRLSQSFEYNYGDFIPVLRPFLRGYLN
*Sba C4H 118 MMYNDMFRIMFDRRFESEEDPLFNKLKALNAERSRLSQSFEYNYGDFIPVLRPFLRGYLN


*Sh*C4H2 SCJFRZ2030D03.g 49 -------------------------------------LLSVQQEYKLTRDHIKAQLVVMF
Zm C4H 241 RCHDLKTRRMKFFEDNFVQERKKVMAQTGEIRCAMDHILEAERKGEINHDNVLYIVENIN
Zm C4H 241 RCHDLKTRRMKFFEDNFVQERKKVMAQTGEIRCAMDHILEAERKGEINHDNVLYIVENIN
*Sh*C4H1 SCCCCL4009H01.g 241 RCHDLKTRRMKVFEDNFVQERKKVMAQTGEIRCAMDHILEAERKGEINHDNVLYIVENIN
*Ss C4H 192 RCHNLKTRRMKVFEDNFVQERKKVMAQTGEIRCAMDHILEAERKGEINHDNVLYIVENIN
*So C4H 178 RCHNLKTRRMKVFEDNFVQERKKVMAQTGEIRCAMDHILEAERKGEINHDNVLYIVENIN
*Sr C4H 179 RCHDLKTRRMKVFEDNFVQERKKVMAQTGEIRCAMDHILEAERKGEINHDNVLYIVENIN
*Sba C4H 178 RCHDLKTRRMKVFEDNFVQERKKVMAQTGEIRCAMDHILEAERKGEINHDNVLYIVENIN


*Sh*C4H2 SCJFRZ2030D03.g 72 QAGTDSSFIVLEYTMIKLMQNPNIMTKLQDEVRMTIPKGKEFVIEDDLNGMSYLKAVIKE
Zm C4H 301 VAAIETTLWSIEWGIAELVNHPAIQHKLREELASVLGAGV-PVTEPDLERLPYLQAIVKE
Zm C4H 301 VAAIETTLWSIEWGIAELVNHPAIQHKLREELASVLGAGV-PVTEPDLERLPYLQAIVKE
*Sh*C4H1 SCCCCL4009H01.g 301 VAAIETTLWSIEWGVAELVNHPAIQSKLREELASVLGAGV-PVTEPDLERLPYLQAIVKE
*Ss C4H 252 VAAIETTLWSIEWGVAELVNHPAIQSKLREELASVLGAGV-PVTEPDLERLPYLQAIVKE
*So C4H 238 VAAIETTLWSIEWGVAELVNHPAIQSKLREELASVLGAGV-PVTEPDLERLPYLQAIVKE
*Sr C4H 239 VAAIETTLWSIEWGVAELVNHPAIQSKLREELASVLGAGV-PVTEPDLERLPYLQAIVKE
*Sba C4H 238 VAAIETTLWSIEWGVAELVNHPAIQSKLREELASVLGAGV-PVTEPDLERLPYLQAIVKE

*Sh*C4H2 SCJFRZ2030D03.g 132 TLRLHGPAPLLVPHLSMAECDIEGYTIPSGTRVIVNAWALARDPTYWESAESFMPERFLE
Zm C4H 360 TLRLRMAIPLLVPHMNLNDGKLAGYDIPAESKILVNAWFLANDPKRWVRPDEFRPERFLE
Zm C4H 360 TLRLRMAIPLLVPHMNLNDGKLAGYDIPAESKILVNAWFLANDPKRWVRPDEFRPERFLE
*Sh*C4H1 SCCCCL4009H01.g 360 TLRLRMAIPLLVPHMNLNDGKLVGYDIPAESKILVNAWFLANDPKRWVRPDEFRPERFLE
*Ss C4H 311 TLRLRMAIPLLVPHMNLNDGKLVGYDIPAESKILVNAWFLANDPKRWVRPDEFRPERFLE
*So C4H 297 TLRLRMAIPLLVPHMNLNDGKLVGYDIPAESKILVNAWFLANDPKRWVRPDEFRPERFLE
*Sr C4H 298 TLRLRMAIPLLVPHMNLNDGKLVGYDIPAESKILVNAWFLANDPKRWVRPDEFRPERFLE
*Sba C4H 297 TLRLRMAIPLLVPHMNLNDGKLVGYDIPAESKILVNAWFLANDPKRWVRPDEFRPERFLE

*Sh*C4H2 SCJFRZ2030D03.g 192 GGSAMTMDYRGNDFHYLPFGAGRRICPGTSFAISAIEIMLANLVYHFNWELPPELKKSGI
Zm C4H 420 EEKS--VEAHGNDFRFVPFGVGRRSCPGIILALPIIGITLGRLVQNFQLLPPPGLDKIDT
Zm C4H 420 EEKS--VEAHGNDFRFVPFGVGRRSCPGIILALPIIGITLGRLVQNFQLLPPPGLDKIDT
*Sh*C4H1 SCCCCL4009H01.g 420 EEKT--VEAHGNDFRFVPFGVGRRSCPGIILALPIIGITLGRLVQNFQLLPPPGQDKIDT
*Ss C4H 371 EEKT--VEAHGNDFRFVPFGVGRRSCPGIILALPIIGITLGRLVQNFHLLQSLV------
*So C4H 357 EEKT--VEAHGNDFRFVPFGVGRRSCPGIILALPIIGITLGRLVQNFHLLQSLV------
*Sr C4H 358 EEKT--VEAHGNDFRFVPFGVGRRSCPGIILALPIIGITLGRLVQNFHLLQSL-------
*Sba C4H 357 EEKT--VEAHGNDFRFVPFGVGRRSCPGIILALPIIGITLGRLVQNFHLLQSLVQSL---


*Sh*C4H2 SCJFRZ2030D03.g 252 DMTESFGV---TVHRTEKLLLVPVLPQN
Zm C4H 478 --TEKPGQFSNQIAKHATIVCKPLEA--
Zm C4H 478 --TEKPGQFSNQIAKHATIVCKPLEA--
*Sh*C4H1 SCCCCL4009H01.g 478 --TEKPGQFSNQIAKHATIVCKPLEA--
*Ss C4H ----------------------------
*So C4H ----------------------------
*Sr C4H ----------------------------
*Sba C4H ----------------------------

**Supplementary Figure S11-4CL**

*Sh*4CL3 SCMCRT2102F02.g 1 MITVAAPEAQPQVAAAAVAPAAPEETVFRSKLPDIDIPSHLPLHEYCFARAAEVADAPCL
*Sba 4CL B 1 MITVAAPEAQPQVAAAAVAPAAPEETVFRSKLPDIDIPSHLPLHEYCFARAAEVADAPCL
Sb 4CL 1 MGSVDTAVAV-PVPVPEPEAEEKAAVVFRSKLPDIEINNSQSLQTYCFGKMSEVADRACL
*Sr 4CL A 1 ------------------------------------------------------------
*Sba 4CL A 1 ------------------------------------------------------------
*Sh*4CL1 SCCCCL3002A03.g 1 MGSVDTAVAV-PVPVAEPAAEEK-AVVFRSKLPDIEINNSQSLHAYCFGKMSEVADRACL
*Ss 4CL A 1 ------------------------------------------------------------
*So 4CL A 1 ------------------------------------------------------------
*Sh*4CL2 SCVPLB1015F12.g 1 ------------------------------------------------------------
Lp 4CL 1 MGSIA-------------ADAPPAELVFRSKLPDIEIPTHLTLQDYCFQRLPELSARACL

*Sh*4CL3 SCMCRT2102F02.g 61 IAAATGRTYTYAETRLLCRKAAASLHGLGVGQGDRVMILLQNSVEFVLTFFGASFLGAVT
*Sba 4CL B 61 IAAATGRTYTYAETRLLCRKAAASLHGLGVGQGDRVMILLQNYVDFVFTFFGASFLGAVT
Sb 4CL 60 IDGQTGASYTYAEVESLSRRAASGLRAMGVGKGDVVMNLLRNCPEFAFTFLGAARLGAAT
*Sr 4CL A 1 ------------------------------------------TSDFVFTFLGAARLGAAT
*Sba 4CL A 1 ------------------------------------------TSDFAFTFFGAARLGAAT
*Sh*4CL1 SCCCCL3002A03.g 59 IDGQTGASYTYAEVESLSRRAASGLRAMGVGKGDVVMNLLRNCPEFAFTFLGAARLGAAT
*Ss 4CL A 1 -----------------------------------------QLADFAFTFLGAARLGAAT
*So 4CL A 1 --------------------------------------------RFAFTFFGAARLGAAT
*Sh*4CL2 SCVPLB1015F12.g 1 ------------------------------------------------------------
Lp 4CL 48 IDGATGAALTYGEVDALSRRCAAGLRRLGVGKGDVVMALLRNCPEFAFVFLGAARLGAAT

*Sh*4CL3 SCMCRT2102F02.g 121 TAANPFCTPLEIHKQFRASGSKLIVTQSAYVDKLRHEAFPRIGAASDGGEDEDNALTVLT

*Sba 4CL B 121 TAANPFCTPLEIHKQFRASGSKLIVTQSAYVDKLRHEAFPRIGAASDGGEDEDNALTVLT

Sb 4CL 120 TTANPFYTPHEIHRQAEAAGAKVIVTEACAVEKVREFAAGR-------------GVPVVT
*Sr 4CL A 19 TTANPFYTPHEIHRQAEAAGAKLIVTEACAVEKVREFAAGR-------------GVPVVT
*Sba 4CL A 19 TTANPFYTPHEIHRQAEAAGAKLIVTEACAVEKVREFAAGR-------------GVPVVT
*Sh*4CL1 SCCCCL3002A03.g 119 TTANPFYTPHEIHRQAEAAGAKLIVTEACAVEKVREFAAGR-------------GVPVVT
*Ss 4CL A 20 TTANPFYTPHEIHRQAEAAGAKLIVTEACAVEKVREFAAGR-------------GVPVVT
*So 4CL A 17 TTANPFYTPHEIHRQAEAAGAKLIVTEACAVEKVREFAAGR-------------GIPVVT
*Sh*4CL2 SCVPLB1015F12.g 1 ------------------------------------------------------------
Lp 4CL 108 TTANPFYTPHEIHRQATAAGARVIVTEACAVEKVRAFAAER-------------GIPVVS

*Sh*4CL3 SCMCRT2102F02.g 181 IDDAANTPEGCLAFWELVTPTDD-AALPEVSISPDDPVALPFSSGTTGLPKGVVLTHGGQ
*Sba 4CL B 181 IDDAANTPEGCLAFWELVTPADD-AALPEVSISPDDPVALPFSSGTTGLPKGVVLTHGGQ
Sb 4CL 167 VD--GRF-DGCVEFAEVIAAE---ELDADADVHPDDVVALPYSSGTTGLPKGVMLTHRSL
*Sr 4CL A 66 VD--GRF-DGCVEFAEVIAAE---ELEADADVHPDDVVALPYSSGTTGLPKGVMLTHRSL
*Sba 4CL A 66 VD--GRF-DGCVEFAEVIAAE---ELEADADVHPDDVVALPYSSGTTGLPKGVMLTHRSL
*Sh*4CL1 SCCCCL3002A03.g 166 VD--GRF-DGCVEFAEVIAAE---ELEADADVHPDDVVALPYSSGTTGLPKGVMLTHRSL
*Ss 4CL A 67 VD--GRF-DGCVEFAEVIAAE---ELEADADVHPDDVVALPYSSGTTGLPKGVMLTHRSL
*So 4CL A 64 VD--GRF-DGCVEFAEVIAAE---ELEADADVHPDDVVALPYSSGTTGLPKGVMLTHRSL
*Sh*4CL2 SCVPLB1015F12.g 1 -------------------MDAAEPLADDEEVDPDDVVALPYSSGTTGMPKGVMLTHRSL
Lp 4CL 155 VD--EGVDGGCLPFAETLLGEESGERFVDEAVDPDDVVALPYSSGTTGLPKGVMLTHRSL

*Sh*4CL3 SCMCRT2102F02.g 240 VSNVAQQVDGANPNLYMREGDVALCVLPLFHIFSLNSVLLCALRAGAAVMLMPKFEMGTM
*Sba 4CL B 240 VSNVAQQVDGANPNLYMREGDVALCVLPLFHIFSLNSVLLCALRAGAAVMLMPKFEMGAM
Sb 4CL 221 ITSVAQQVDGENPNLYFSKDDVVLCLLPLFHIYSLNSVLLAGLRAGSTIVIMRKFDLGAL
*Sr 4CL A 120 ITSVAQQVDGENPNLYFSKDDVLLCLLPLFHIYSLNSVLLAGLRAGSTIVIMRKFDLGAL
*Sba 4CL A 120 ITSVAQQVDGENPNLYFSKDDVLLCLLPLFHIYSLNSVLLAGLRAGSTIVIMRKFDLGAL
*Sh*4CL1 SCCCCL3002A03.g 220 ITSVAQQVDGENPNLYFSKDDVLLCLLPLFHIYSLNSVLLAGLRAGSTIVIMRKFDLGAL
*Ss 4CL A 121 ITSVAQQVDGENPNLYFSKDDVLLCLLPLFHIYSLNSVLLAGLRAGSTIVIMRKFDLGAL
*So 4CL A 118 ITSVAQQVDGENPNLYFSKDDVLLCLLPLFHIYSLNSVLLAGLRAGSTIVIMRKFDLGAL
*Sh*4CL2 SCVPLB1015F12.g 42 VTSVAQQVDGENPNLYFSSDDVVLCVLPLFHIYSLNSVLLAGLRAGCAIVIMRKFEIGAL
Lp 4CL 213 VTSVAQQVDGENPNLHFSSSDVLLCVLPLFHIYSLNSVLLAGLRAGCAIVIMRKFDHGAL

*Sh*4CL3 SCMCRT2102F02.g 300 LEGHTGDIG---------------------------------------------------
*Sba 4CL B 300 LEGIQRWRVTVAAVVPPAGCSRW--PR---------------------------------
Sb 4CL 281 VDLVRKHGITIAPFVPPIVVEIAKSPRVTADDLASIRMVMSGAAPMGKELQDAFMTKIPN
*Sr 4CL A 180 VDLVRKHAITIAPFVPPIVVEIAKSPRVTAADLASIRMVMSGAAPMGKELQDAFMTKIPN
Sba 4CL A 180 VDLVRKHAITIAPFVPPIVVEIAKSPRVTAADLASIRMVMSGAAPMGKELQDAFMTKIPN
*Sh*4CL1 SCCCCL3002A03.g 280 VDLVRKHAITIAPFVPPIVVEIAKSPRVTAADLASIRMVMSGAAPMGKELQDAFMTKIPN
*Ss 4CL A 181 VDLVRKHAITIAPFVPPIVVEIAKSPRVTAADLASIRMVMSGAAPMGKELQDAFMTKIPN
*So 4CL A 178 VDLVRKHAITIAPFVPPIVVEIAKSPRVTAADLASIRMVMSGAAPMGKELQDAFMTKIPN
*Sh*4CL2 SCVPLB1015F12.g 102 VELVRAHGVTVAPFVPPIVVEIAKSPRVGAHDLASIRMVMSGAAPMGKDLQDAFMAKIPN
Lp 4CL 273 VDLVRTHGVTVAPFVPPIVVEIAKSARVTAADLASIRLVMSGAAPMGKELQDAFMAKIPN

*Sh*4CL3 SCMCRT2102F02.g ------------------------------------------------------------
*Sba 4CL B ------------------------------------------------------------
Sb 4CL 341 AVLGQGYGMTEAGPVLAMCLAFAKEPFQVKSGSCGTVVRNAELKVVDPDTGAALGRNQPG
*Sr 4CL A 240 AVLGQGYGMTEAGPVLAMCLAFAKEPFQVKSGSCGTVVRNAELKIVDPDTGAALGRNQPG
*Sba 4CL A 240 AVLGQGYGMTEAGPVLAMCLAFAKEPFQVKSGSCGTVVRNAELKIVDPDTGAALGRNQPG
*Sh*4CL1 SCCCCL3002A03.g 340 AVLGQGYGMTEAGPVLAMCLAFAKEPFQVKSGSCGTVVRNAELKIVDPDTGAALGRNQPG
*Ss 4CL A 241 AVLGQGYGMTEAGPVLAMCLAFAKEPFQVKSGSCGTVVRNAELKIVDPDTGAALGRNQPG
*So 4CL A 238 AVLGQGYGMTEAGPVLAMCLAFAKEPFQVKSGSCGTVVRNAELKIVDPDTGAALGRNQPG
*Sh*4CL2 SCVPLB1015F12.g 162 AVLGQGYGMTEAGPVLAMCLAFAKEPFEVKSGSCGTVVRNAELKIVDPDTSASLGRNQPG
Lp 4CL 333 AVLGQGYGMTEAGPVLAMCLAFAKEPFAVKSGSCGTVVRNAELKIVDPDTGASLGRNLPG

*Sh*4CL3 SCMCRT2102F02.g ------------------------------------------------------------
*Sba 4CL B ------------------------------------------------------------
Sb 4CL 401 EICIRGEQIMKGYLNDLESTKNTIDKDGWLHTGDIGYVDDDDEIFIVDRLKEIIKYKGFQ
*Sr 4CL A 300 EICIRGEQIMKGYLNDPESTKNTIDKDGWLHTGDIGYVDDDDEIFIVDRLKEIIKYKGFQ
*Sba 4CL A 300 EICIRGEQIMKGYLNDPESTNNTIDKGGWLHTGDIGYVDDDDEIFIVDRLKEIIKYKGFQ
*Sh*4CL1 SCCCCL3002A03.g 400 EICIRGEQIMKGYLNDPESTKNTIDKDGWLHTGDIGYVDDDDEIFIVDRLKEIIKYKGFQ
*Ss 4CL A 301 EICIRGEQIMKGYLNDPESTKNTIDKDGWLHTGDIGYVDDDDEIFIVDRLKEIIKYKGFQ
*So 4CL A 298 EICIRGEQIMKGYLNDPESTKNTIDKDGWLHTGDIGYVDDDDEIFIVDRLKEIIKYKGFQ
*Sh*4CL2 SCVPLB1015F12.g 222 EICIRGEQIMKGYLNDPEATKNTIDKDGWLHTGDIGYVDDDDEIFIVDRLKEIIKYKGFQ
Lp 4CL 393 EICIRGKQIMKGYLNDPVATKNTIDKDGWLHTGDIGYVDDDDEIFIVDRLKEIIKYKGFQ

*Sh*4CL3 SCMCRT2102F02.g ------------------------------------------------------------
*Sba 4CL B ------------------------------------------------------------
Sb 4CL 461 VPPAELEALLITHPEIKDAAVVSMKDDLAGEIPVAFIVRTEGSEVTEDEIKQFVAKEVVF
*Sr 4CL A 360 VPPAELEALLITHPEIKDAAVVSMKDDLAGEIPVAFIVRTEGSEVTEDEIKQFVAKEVVF
*Sba 4CL A 360 VPPAELEALLITHPEIKDAAVVSMKDDLAGEIPVAFIVRTEGSEVTEDVIKQFVAKEVVF
*Sh*4CL1 SCCCCL3002A03.g 460 VPPAELEALLITHPEIKDAAVVSMKDDLAGEIPVAFIVRTEGSEVTEDEIKQFVAKEVVF
*Ss 4CL A 361 VPPAELEALLITHPEIKDAAVVSMKDDLAGEIPVAFIVRTEGSEVTEDEIKQFVAKEVVF
*So 4CL A 358 VPPAELEALLITHPEIKDAAVVSMKDDLAGEIPVAFIVRTEGSEVTEDEIKQFVAKEVVF
*Sh*4CL2 SCVPLB1015F12.g 282 VPPAELEALLITHPEIKDAAVVSMKDELAGEVPVAFIIRSEGSEISENEIKQFVAKEVVF
Lp 4CL 453 VPPAELEALLITHPEIKDAAVVSMQDELAGEVPVAFVVRTEGSEISENEIKQFVAKEVVF

*Sh*4CL3 SCMCRT2102F02.g --------------------------------------------
*Sba 4CL B --------------------------------------------
Sb 4CL 521 YKKIHKVFFTESIPKNPSGKILRKDLRARLAAGVH---------
*Sr 4CL A 420 LQESL---------------------------------------
*Sba 4CL A 420 YKNH----------------------------------------
*Sh*4CL1 SCCCCL3002A03.g 520 YKKVHKVFFTESIPKNPSGKILRKDLRARLAAGV----------
*Ss 4CL A 421 YKNHI---------------------------------------
*So 4CL A 418 YKNH----------------------------------------
*Sh*4CL2 SCVPLB1015F12.g 342 YKRINRVFFTDSIPKNPSGKILRKDLRARLAAGIPSSDNTQSKS
Lp 4CL 513 YKRICKVFFADSIPKSPSGKILRKDLRAKLAAGIPSSNTTQSKS

**Supplementary Figure S12-HCT**

*Ss HCT 1 -----RIH--LGSEMVYPAAETPRRRLWNSGPDLVVP-RFHTPSVYFFRRRDADGNDLTA
Sb HCT 1 -----MKITVRGSEMVYPAAETPRRRLWNSGPDLVVP-RFHTPSVYFFRRRDADGNDLTA
*Sh*HCT1 SCCCCL4009E02.g 1 -----MKITVRGSEMVYPAAETPRRRLWNSGPDLVVP-RFHTPSVYFFRRRDADGNDLTA
*So HCT 1 -------H--LGSEMVYPAAETPRRRLWNSGPDLVVP-RFHTPSVYFFRRRDADGNDLTA
*Sr HCT 1 -------H--LGSEMVYPAAETPRRRLWNSGPDLVVP-RFHTPSVYFFRRRDADGNDLTA
*Sba HCT 1 -------H--LGSEMVYPAAETPRRRLWNSGPDLVVP-RFHTPSVYFFRRRDADGNDLTA
*Sh*HCT-like_SCVPRT2082B07.g 1 MNTMDSEVQVVESSFIVPNEPTPREGLWLSPLDLIVANRGHTPTVYLYSYNNV------A
Pv HCT-like 1a 1 -MKQAEVVVVVDTALVPPSKETPGQPLWLSNLDLAVP-RTHTPLVYYYPAPAQG---AAA


*Ss HCT 53 PDGSFFDGARMRRALAEALVPFYPMAGRLARDE-DGRVEIDCNAAGVLFQEADAPDATID
Sb HCT 55 ADGSFFDGARMRRALAEALVPFYPMAGRLARDE-DGRVEIDCNAAGVLFQEADAPDATID
*Sh*HCT1 SCCCCL4009E02.g 55 PDGSFFDGARMRRALAEALVPFYPMAGRLARDE-DGRVEIDCNAAGVLFQEADAPDATID
*So HCT 51 PDGSFFDGARMRRALAEALVPFYPMAGRLARDE-DGRVEIDCNAAGVLFQEADAPDATID
*Sr HCT 51 PDGSFFDGARMRRALAEALVPFYPMAGRLARDE-DGRVEIDCNAAGVLFQEADAPDATID
*Sba HCT 51 PDGSFFDGARMRRALAEALVPFYPMAGRLARDE-DGRVEIDCNAAGVLFQEADAPDATID
*Sh*HCT-like SCVPRT2082B07.g55 TADDFFDVARLKEAMAKALAAFYPLAGRLGVNDDDDRMEISCNGEGALFVVAHADDLSVE
Pv HCT-like 1a 56 AGTGSFAPDRLTAALAGALVPFYPLAGRLGPGP-DGRPQINCTGEGALFVVARADLTGDD


*Ss HCT 112 YFGDFAPTMELKRLIPTVDFSDDTAFPLLVLQVTHFKCGGVAIGVGMQHHVADGFSGLHF
Sb HCT 114 YFGDFAPTMELKRLIPTVDFSDDTAFPLLVLQVTHFKCGGVAIGVGMQHHVADGFSGLHF
*Sh*HCT1 SCCCCL4009E02.g 114 YFGDFAPTMELKRLIPTVDFSDDTAFPLLVLQVTHFKCGGVAIGVGMQHHVADGFSGLHF
*So HCT 110 YFGDFAPTMELKRLIPTVDFSDDTAFPLLVLQVTHFKCGGVAIGVGMQHHVADGFSGLHF
*Sr HCT 110 YFGDFAPTMELKRLIPTVDFSDDTAFPLLVLQVTHFKCGGVAIGVGMQHHVADGFSGLHF
*Sba HCT 110 YFGDFAPTMELKRLIPTVDFSDDTAFPLLVLQVTHFKCGGVAIGVGMQHHVADGFSGLHF
*Sh*HCT-like SCVPRT2082B07.115 DIKEFKPSPELRRLFVPRIEPSSI---MLAIQVTFLKCGGVALGTALHHVAIDASSAFHF
Pv HCT-like 1a 115 IFEDFEPSPEIRRAFVPSPRPGDASCPLAVFQLTFLKCGGVVLGTGIHHAVLDGVGAFQF


*Ss HCT 172 INSWADLCRGAPI----AVMPFIDRSLLRXXDPPTPAYPHIEYQPAPAMLSSEPPQAALT
Sb HCT 174 INSWADLCRGVPI----AVMPFIDRSLLRARDPPAPVYPHVEYQPAPAMLSSEPPQAALT
*Sh*HCT1 SCCCCL4009E02.g 174 INSWADLCRGVPI----AVMPFIDRSLLRARDPPTPAYPHIEYQPAPAMLSSEPPQAALT
*So HCT 170 INSWADLCRGVPI----AVMPFIDRSLLRARDPPTPAYPHIEYQPAPAMLSSEPPQAALT
*Sr HCT 170 INSWADLCRGVPI----AVMPFIDRSLLRARDPPTPAYPHIEYQPAPAMLSSEPPQAALT
*Sba HCT 170 INSWADLCRGVPI----AVMPFIDRSLLRARDPPTPAYPHIEYQPAPAMLSSEPPQAALT
*Sh*HCT-like SCVPRT2082B07.g172 FKTWSAFSKHGDR--AAVELPCHDRTLLRARFPPTVHPDRT--------LDIAP------
Pv HCT-like 1a 175 IQTWAGLARGLDAAEACGPVPFHDXTLLRAXVPAVPXLR-P--------XSSTPRXSSAA


*Ss HCT 228 AKPATPAAAVAIFRLSRAELGRLRSQLPAR--EGAPRFSTYAVLAAHVWRCASLARGLPA
Sb HCT 230 AKPATPPAAVAIFKLSRAELGRLRSQVPAREREGAPRFSTYAVLAAHVWRCASLARGLPA
*Sh*HCT1 SCCCCL4009E02.g 230 AKPATPPAAVAIFKLSRAELGRLRSQVPAR--EGAPRFSTYAVLAAHVWRCASLARGLPA
*So HCT 226 AKPATPPAAVAIFKLSRAELGRLRSQVPAR--EDAPRFSTYAVLAAHVWRCASLARGLPA
*Sr HCT 226 AKPATPPAAVAIFKLSRAELGRLRSQLPAR--EGAPGFSTYAVLAAHVWRCASLARGLPA
*Sba HCT 226 AKPATPPAAVAIFKLSRAELGRLRSQVPAR--EGAPRFSTYAVLAAHVWRCASLARGLPA
*Sh*HCT-like SCVPRT2082B07.g ------------------------------------------------------------
Pv HCT-like 1a 226 ---ARAPXVTRFYAVSPKLLADLKS-------RCGAGVSTYCAVTAHLWRCVCVARGMAP


*Ss HCT 286 DQPTKLYCATDGRQRLQPPLPEGYFGNVIFTATPLANAGTVTA-GVAEGAGVIQAALDRM
Sb HCT 290 DQPTKLYCATDGRQRLQPPLPEGYFGNVIFTATPLANAGTVTA-GVAEGAAVIQAALDRM
*Sh*HCT1 SCCCCL4009E02.g 288 DQPTKLYCATDGRQRLQPPLPEGYFGNVIFTATPLANAGTVTA-GVAEGAGVIQAALDRM
*So HCT 284 DQPTKLYCATDGRQRLQPPLPEGYFGNVIFTATPLANAGTVTA-GVAEGAGVIQEALDRM
*Sr HCT 284 DQPTKLYCATDGRQRLQPPLPEGYFGNVIFTATPLANAGTVTA-GVAEGAGVIQAALDRM
*Sba HCT 284 DQPTKLYCATDGRQRLQPPLPEGYFGNVIFTATPLANAGTVTA-GVAEGAGVIQAALDRM
*Sh*HCT-like SCVPRT2082B07.g ------------------------------------------------------------
Pv HCT-like 1a 276 GADTRLGLPANVRHRLSPPLPRSFFGNAVVRDLVTARVGDLLGSPLGSVAETIKKAVDGV


*Ss HCT 345 DDGYCRSALDYLELQPDLS-----ALVRGAHTFRCPNLGLTSWVRLPIHDADFGWGRPVF
Sb HCT 349 DDGYCRSALDYLELQPDLS-----ALVRGAHTFRCPNLGLTSWVRLPIHDADFGWGRPVF
*Sh*HCT1 SCCCCL4009E02.g 347 DDGYCRSALDYLELQPDLS-----ALVRGAHTFRCPNLGLTSWVRLPIHDADFGWGRPVF
*So HCT 343 DDGYCRSALDYLELQPDLS-----ALVRGAHTFRCPNLGLTSWVRLPIHDADFGWGRPVF
*Sr HCT 343 DDGYCRSALDYLELQPDLS-----ALVRGAHTFRCPNLGLTSWVRLPIHDADFGWGRPVF
*Sba HCT 343 DDGYCRSALDYLELQPDLS-----ALVRGAHTFRCPNLGLTSWVRLPIHDADFGWGRPVF
*Sh*HCT-like SCVPRT2082B07.g ------------------------------------------------------------
Pv HCT-like 1a 336 GDAFVRSVLDYLELEQAKKRGGDDDSAQGEQMVPATDLWAVSWLGMPMYSADFGSGAPRF

*Ss HCT 400 MGPGGIAYEGLAFVLPSANRDGSLSVAISLQAEHM----------
Sb HCT 404 MGPGGIAYEGLAFVLPSANRDGSLSVAISLQAEHMEKFRKLIYDF
*Sh*HCT1 SCCCCL4009E02.g 402 MGPGGIAYEGLAFVLPSANRDGSLSVAISLQAEHMEKFRKLIYDF
*So HCT 398 MGPGGIAYEGLAFVLPSANRDGSLSVAISLQAEHMEKFRITS---
*Sr HCT 398 MGPGGIAYEGLAFVLPSANRDGSLSVAISLQAEHMEKFRI-----
*Sba HCT 398 MGPGGIAYEGLAFVLPSANRDGSLSVAISLQAEHMEKFRI-----
*Sh*HCT-like SCVPRT2082B07.g ---------------------------------------------
Pv HCT-like 1a 396 VAPAQMFGVGTAYMTPCANKDDGITVIFSMEAEHIECFEKVFYGV

**Supplementary Figure S13- C3H**

*Ss C3H 1 ------------------------------------------------KPIRCRCFQEWA
*Sr C3H 1 -----------------------------------------SINLRQIKPIRCRCFQEWA
*Sba C3H 1 ------------------------------------------INLRQIKPIRCRCFQEWA
*So C3H 1 ------------------------------------------INLRQIKPIRCRCFQEWA
*Sh*C3H1 SCVPCL6041E07.g 1 MDASLLLSVALA-VVLIPLSLALLNRLRLGRLPPGPRPWPVLGNLRQIKPIRCRCFQEWA
Sb C3H 1 MDASLLLSVALA-VVLIPLSLALLNRLRLGRLPPGPRPWPVLGNLRQIKPIRCRCFQEWA
*Sh*C3H2 SCQSRT1036E09.g 1 ------------------------------------------------------------
Pv C3H 1 MNAASFLAVALALAALVPVSLLLLNRLLYGKLPPGPRPRPVVGNLFDVQPVRCRCYQEWA


*Ss C3H 13 ERYGPVISVWFGSGLTVVVSTSELAKEVLKENDQQLADRPRNRSTQRFSRNGQDLIWADY
*Sr C3H 20 ERYGPVISVWFGSGLTVVVSTSELAKEVLKENDQQLADRPRNRSTQRFSRNGQDLIWADY
*Sba C3H 19 DRYGPVISVWFGSGLTVVVSTSELAKEVLKENDQQLADRPRNRSTQRFSRNGQDLIWADY
*So C3H 19 DRYGPVISVWFGSGLTVVVSTSELAKEVLKENDQQLADRPRNRSTQRFSRNGQDLIWADY
*Sh*C3H1 SCVPCL6041E07.g 60 DRYGPVISVWFGSGLTVVVSTSELAKEVLKENDQQLADRPRNRSTQRFSRNGQDLIWADY
Sb C3H 60 ERYGPVISVWFGSGLTVVVSTSELAKEVLKENDQQLADRPRNRSTQRFSRNGQDLIWADY
*Sh*C3H2 SCQSRT1036E09.g 1 ------MTVWLGTSPTVVVSTSELAKEVLKTHDQQLADRCRDRSTESFSRGGQDLIWADY
Pv C3H 61 GRYGPIMTVWLGSQPTVVVSTAELAREVLKTHDQSLADRVRDRSSERFSRGGKDLIWADY


*Ss C3H 73 GPHYIKVRKLCNLELFTPKRLEALRPIREDEVTAMVESVYRAATAPGNEGKPLVVRNHLS
*Sr C3H 80 GPHYIKVRKLCNLELFTPKRLEALRPIREDEVTAMVESVYRAATAPGNEGKPLVVRNHLS
*Sba C3H 79 GPHYIKVRKLCNLELFTPKRLEALRPIREDEVTAMVESVYRAATAPGNEGKPLVVRNHLS
*So C3H 79 GPHYIKVRKLCNLELFTPKRLEALRPIREDEVTAMVESVYRAATAPGNEGKPLVVRNHLS
*Sh*C3H1 SCVPCL6041E07.g 120 GPHYIKVRKLCNLELFTPKRLEALRPIREDEVTAMVESVYRAATSPGNEGKPLVVRNHLS
Sb C3H 120 GPHYIKVRKLCNLELFTPKRLEALRPIREDEVTAMVESVYRAATAPGNEGKPMVVRNHLS
*Sh*C3H2 SCQSRT1036E09.g 55 GPHYIKVRKLCNLELFTQRRLEALRPIREDEVTAMVESVYKAVTSPGNEGKPLVVKNHLS
Pv C3H 121 GAHYIKVRKLCNLELFTPRRLEALRPIREDEVTAMVESVHRAVTAPGNEGKPLVVKNHLS


*Ss C3H 133 MVAFNNITRLAFGKRFMNANADIDEQGREFKTIVNNGIKIGASLSVAEFIWYLRWLCPLN
*Sr C3H 140 MVAFNNITRLAFGKRFMNANGDIDEQGREFKTIVNNGIKIGASLSVAEFIWYLRWLCPLN
*Sba C3H 139 MVAFNNITRLAFGKRFMNANGDIDEQGHEFKTIVNNGIKIGASLSVAEFIWYLRWLCPLN
*So C3H 139 MVAFNNITRLAFGKRFMNANGDIDEQGREFKTIVNNGIKIGASLSVAEFIWYLRWLCPLN
*Sh*C3H1 SCVPCL6041E07.g 180 MVAFNNITRLAFGKRFMNANGDIDEQGREFKTIVNNGIKIGASLSVAEFIWYLRWLCPLN
Sb C3H 180 MVAFNNITRLAFGKRFMNANGDIDEQGREFKTIVNNGIKIGASLSVAEFIWYLRWLCPLN
*Sh*C3H2 SCQSRT1036E09.g 115 MVAFNNITRLAFGKRFVNAAGELDEQGREFKGIVHNGIKIGASLSIAQHIPWLRWLAPVD
Pv C3H 181 MVAFNNITRLAFGKRFVNAAGELDEQGREFKGIVTDGIKIGASLSIARYIPWLKWLTPAD


*Ss C3H 193 EELYKTHNERRDRLTMKIIDEHAKSLKESGAKQHFVDALFTLKEQYDLSEDTVIGLLWDM
*Sr C3H 200 EELYKTHNERRDRLTMKIIEEHAKSLKESGAKQHFVDALFTLKEQYDLSEDTVIGLLWDM
*Sba C3H 199 EELYKTHNERRDRLTMKIIEEHAKSLKESGAKQHFVDALFTLKEQYDLSEDTVIGLLWDM
*So C3H 199 EELYKTHNERRDRLTMKIIEEHAKSLKESGAKQHFVDALFTLKEQYDLSEDTVIGLLWDM
*Sh*C3H1 SCVPCL6041E07.g 240 EELYKTHNERRDRLTMKIIDEHAKSLKESGAKQHFVDALFTLKEQYDLSEDTVIGLLWDM
Sb C3H 240 EELYKTHNERRDRLTMKIIEEHAKSLKESGAKQHFVDALFTLKQQYDLSEDTVIGLLWDM
*Sh*C3H2 SCQSRT1036E09.g 175 EQVFKAHGERRDRLTVKIMEEHAKALKQRGAQQHFVDALFTLRDKYDLSDDTVIGLLWDM
Pv C3H 241 NEVFKAHGDRRDRLTVKIMEEHAKARKQRGAQQHFVDALFTLREQYDLSDDTVIGLLWDM


*Ss C3H 253 ITAGMDTTVISVEWAMAELVRNPRVQKKLQEELDRVVGRDRVMLETDFQNLPYLQAVVKE
*Sr C3H 260 ITAGMDTTVISVEWAMAELVRNPRVQKKLQEELDRVVGRDRVMLETDFQNLPYLQAVVKE
*Sba C3H 259 ITAGMDTTVISVEWAMAELVRSPRVQKKLQEELDRVVGRDRVMLETDFQNLPYLQAVVKE
*So C3H 259 ITAGMDTTVISVEWAMAELVRNPRVQKKLQEELDRVVGRDRVMLETDFQNLPYLQAVVKE
*Sh*C3H1 SCVPCL6041E07.g 300 ITAGMDTTVISVEWAMAELVRNPRVQKKLQEELDRVVGRDRVMLETDFQNLPYLQAVVKE
Sb C3H 300 ITAGMDTTVISVEWAMAELVRNPRVQKKLQEELDRVVGRDRVMLETDFQNLPYLQAVVKE
*Sh*C3H2 SCQSRT1036E09.g 235 ITAGTDTTVISVEWAMAELLRNPRVQEKLQEELDHVVGRDRVLSETDFPNLPYLQAVVKE
Pv C3H 301 ITAGTDTTVISVEWAMAELVRNPRVQEKAQEELDRVVGRDRVLLETDFPNLPYLQALVKE


*Ss C3H 313 SLRLHPPTPLMLPHKASTNVKIGGYDIPKGANVMVNVWAVARDPKVWSNPLEYRPERFLE
*Sr C3H 320 SLRLHPPTPLMLPHKASTNVKIGGYDIPKGANVMVNVWAVARDPKVWSNPLEYRPERFLE
*Sba C3H 319 SLRLHPPTPLMLPHKASTNVKIGGYDIPKGANVMVNVWAVARDPKVWSNPLQYRPERFLE
*So C3H 319 SLRLHPPTPLMLPHKASTNVKIGGYDIPKGANVMVNVWAVARDPKVWSNPLQYRPERFLE
*Sh*C3H1 SCVPCL6041E07.g 360 SLRLHPPTPLMLPHKASTNVKIGGYDIPKGANVMVNVWAVARDPKVWSNPLEYRPERFLE
Sb C3H 360 SLRLHPPTPLMLPHKASTNVKIGGYDIPKGANVMVNVWAVARDPKVWSNPLEYRPERFLE
*Sh*C3H2 SCQSRT1036E09.g 295 SLRLHPPTPLMLPHRASASVKIAGYDIPKGANVVVNVWAVARDPAVWDSPLEFRPERFLR
Pv C3H 361 SLRLHPPTPLMLPHKASASVKIAGYDIPKGATVIVNVWAVARDPEVWDDPLEFRPERFLQ
*Ss C3H 373 ENIDIKGSDFRVLPFGAGRRVCPGAQLGINLVASMIGHLLHHFEWSLPEGTRPEDVNMME
*Sr C3H 380 ENIDIKGSDFRVLPFGAGRRVCPGAQLGINLVASMIGHLLHHFEWSLPEGTRPEDVNMME
*Sba C3H 379 ENIDIKGSDFRVLPFGAGRRVCPGAQLGINLVASMIGHLLHHFEWSLPEGTRPEDVNMME
*So C3H 379 ENIDIKGSDFRVLPFGAGRRVCPGAQLGINLVASMIGHLLHHFEWSLPEGTRPEDVNMME
*Sh*C3H1 SCVPCL6041E07.g 420 ENIDIKGSDFRVLPFGAGRRVCPGAQLGINLVASMIGHLLHHFEWSLPEGTRPEDINMME
Sb C3H 420 ENIDIKGSDFRVLPFGAGRRVCPGAQLGINLVASMIGHLLHHFEWSLPEGTRPEDVNMME
*Sh*C3H2 SCQSRT1036E09.g 355 ENIDIKGADFRVLPFGAGRRVCPGAQLGINLVASMIGHMLHHFRWTLPEGTRPEDVSMME
Pv C3H 421 ENIDIKGADFRVLPFGAGRRVCPGAQLGINLVTTMLGHMLHHFSWSLPEGTRPEDVEMME


*Ss C3H 433 SPGLVTFMGTITQL-------------------
*Sr C3H 440 SPGLVTFMGTNH---------------------
*Sba C3H 439 SPGLVTFMGTITS--------------------
*So C3H 439 SPGLVTFMGTITS--------------------
*Sh*C3H1 SCVPCL6041E07.g 480 SPGLVTFMGTPLQAVAKPRLEMEELYNRVPVEM
Sb C3H 480 SPGLVTFMGTPLQAVAKPRLEKEELYNRVPVEM
*Sh*C3H2 SCQSRT1036E09.g 415 SPGLVTFMATPLQAVATPRLDKEELYRRVPSEI
Pv C3H 481 TPGLVTFMATPLKAVATPRLDREELYRRVPSEM

**Supplementary Figure S14-CCoAOMT**

Pv CCoAOMT 1 MASTAAEA--AKAAEQPANGNGEQKTRHSEVGHKSLLKSDDLYQYILDTSVYPREPESMK
*Sh*CCoAOMT1 SCCCLR1069B09.g 1 MATTATEA--AKAA-PAEQANGEQKTRHSEVGHKSLLKSDDLYQYILDTSVYPREPESMK
*Sba CCoAOMT B 1 ILKLCI-----------QRVGSSPIWSTCRRPRIHFLKSEALYQYILDTSVYPREPESMK
*Ss CCoAOMT B 1 ----------------------------------------TLYQYILDTSVYPREPESMK
*Sr CCoAOMT B 1 ---------------------------------LVILKSEALYQYILDTSVYPREPESMK
*So CCoAOMT B 1 --------------------------------GNSILKSEALYQYILDTSVYPREPESMK
Pv CCoAOMT 1 --------------MAAGGDTIARVHTGIDSSNKTLLKSEALYKYVLDTSVLPHEPECMR
*Ss CCoAOMT A 1 -----------------------------------------LYKYVLDTSVLPHEPDCMR
*Sh*CCoAOMT2 SCJLRT2050C09.g 1 -------------MAPSGDTAIAQVHTGLDSSNKTLLKSEALYKYVLDTSVLPHEPDCMR
*Sba CCoAOMT A 1 -------------IQRVGS---SPIWSTCRRPRIHFLKSEDLYKYVLDTSVLPHEPDCMR
*Sr CCoAOMT A 1 --------------------------------------------------VLPHEPDCMR
*So CCoAOMT A 1 ----------------------------AGGRGNYFLKSEDLYKYVLDTSVLPHEPDCMR
*Sh*CcoAOMT3 SCJFRZ2010H06.g 1 ---------MDPGMATGGGGSIPDVHSNTDSSNKTLLKSQALYKYILDTTVLPNEPECMR
Pv CCoAOMT 1 MDTTASGRGGDPAMATAGGATAPNVHSNTDSSNKTLLKSQALYKYVLDTTVLPNEPECLR


Pv CCoAOMT 59 ELREITAKHPWNLMTTSADEGQFLNMLIKLIGAKKTMEIGVYTGYSLLATALALPEDGTI
*Sh*CCoAOMT1 SCCCLR1069B09.g 58 ELREITAKHPWNLMTTSADEGQFLNMLIKLIGAKKTMEIGVYTGYSLLATALALPEDGTI
*Sba CCoAOMT B 50 ELREITAKHPWNLMTTSADEGQFLNMLIKLIGAKKTMEIGVYTGYSLLATALALPEDGTI
*Ss CCoAOMT B 21 ELREITAKHPWNLMTTSADEGQFLNMLIKLIGAKKTMEIGVYTGYSLLATALALPEDGTI
*Sr CCoAOMT B 28 ELREITAKHPWNLMTTSADEGQFLNMLIKLIGAKKTMEIGVYTGYSLLATALALPEDGTI
*So CCoAOMT B 29 ELREITAKHPWNLMTTSADEGQFLNMLIKLIGAKKTMEIGVYTGYSLLATALALPEDGTI
Pv CCoAOMT 47 ELRLVTDKHEWGLMQSSPDEAQLLRMLIKLMGARNTIEVGVFTGYSLLATALALPADGRV
*Ss CCoAOMT A 20 ELRLVTDKHEWGFMQSSPDEAQLLRMLLKLTGARNTLEVGVFTGYSLLATALALPDDGKV
*Sh*CCoAOMT2 SCJLRT2050C09.g 48 ELRLVTDKHEWGFMQSSPDEAQLLRMLLKLTGARNTLEVGVFTGYSLLATALALPDDGKV
*Sba CCoAOMT A 45 ELRLVTDKHEWGFMQSSPDEAQLLRMLLKLTGARNTLEVGVFTGYSLLATALALPDDGKV
*Sr CCoAOMT A 11 ELRLVTDKHEWGFMQSSPDEAQLLRMLLKLTGARNTLEVGVFTGYSLLATALALPDDGKV
*So CCoAOMT A 33 ELRLVTDKHEWGFMQSSPDEAQLLRMLLKLTGARNTLEVGVFTGYSLLATALALPDDGKV
*Sh*CcoAOMT3 SCJFRZ2010H06.g 52 ELRLLTDKHERRYMATPPDEAQLLRMLIKLSGARNAIEVGVFTGCSLLATALALPHDGKV
Pv CCoAOMT 61 ELRLLTDKHERRNMATPPDEAQLLRMLIRLMGARNTIEVGVFTGCSLISTALALPADGRV


Pv CCoAOMT 119 LAMDINRENYELGLPCIEKAGVAHKIDFREGPALPVLDDLIADEKNHGTFDFAFVDADKD
*Sh*CCoAOMT1 SCCCLR1069B09.g 118 LAMDINRENYELGLPCIEKAGVAHKIDFREGPALPVLDDLIADEKNHGSFDFVFVDADKD
*Sba CCoAOMT B 110 LAMDINRENYELGLPCIEKAGVAHKIDFREGPALPVLDDLIADEKNHGSFDFVFVDADKD
*Ss CCoAOMT B 81 LAMDINRENYELGLPCIEKAGVAHKIDFHEGPALPVLDDLIADEKNHGSFDFVFVDADKD
*Sr CCoAOMT B 88 LAMDINRENYELGLPCIEKAGVAHKIDFREGPALPVLDDLIADEKNHGSFDFVFVDADKD
*So CCoAOMT B 89 LAMDINRENYELGLPCIEKAGVAHKIDFREGPALPVLDDLIADEKNHGSFDFVFVDADKD
Pv CCoAOMT 107 IAIDVDREYYEIGRPFIEKAGVAGKVDFREGPALDHLDALLADERNLGAFDFAFVDADKP
*Ss CCoAOMT A 80 IAFDVSREYYDIGRPFIEKAGVAHKVDFREGPALEGLDALLADEANHGAFDFAFVDADKP
*Sh*CCoAOMT2 SCJLRT2050C09.g 108 IAFDVSREYYDIGRPFIDKAGVAHKVDFREGPALEGLDALLADEANHGAFDFAFVDADKP
*Sba CCoAOMT A 105 IAFDVSREYYDIGRPFIEKAGVAHKVDFREGPALEGLDALLADEANHGAFDFAFVDADKP
*Sr CCoAOMT A 71 IAFDVSREYYDIGRPFINKAGVAHKVDFRGGPALEGLDALLADEANHGAFDFAFVDADKP
*So CCoAOMT A 93 IAFDVSREYYDIGRPFIEKAGVAHKVDFREGPALEGLDALLADEANHGAFDFAFVDADKP
*Sh*CcoAOMT3 SCJFRZ2010H06.g 112 VAIDVSREYYELGLPFIEKAGVAHKVDFREGPALERLDALLADDANHGAFDFAFVDADKP
Pv CCoAOMT 121 VAIDVSREYYEVGRPFFEKAGVAHKVDFREGPALERLHELLADARNLGAFDFAFVDADKP


Pv CCoAOMT 179 NYLNYHERLLKLVKLGGLIGYDNTLWNGSVVLPDDAPMRKYIRYYRDFVLVLNKALAADE
*Sh*CCoAOMT1 SCCCLR1069B09.g 178 NYLNYHERLLKLVKLGGLIGYDNTLWNGSVVLPDDAPMRKYIRFYRDFVLVLNKALAADE
*Sba CCoAOMT B 170 NYLNYHERLLKLVKLGGLIGYDNTLWNGSVVLPDDAPMRKYIRFYRDFVLVLNKALAADE
*Ss CCoAOMT B 141 NYLNYHERLLKLVKLGGLIGYDNTLWNGSVVLPDDAPMRKYIRFYRDFVLVLNKALAADE
*Sr CCoAOMT B 148 NYLNYHERLLKLVKLGGLIGYDNTLWNGSVVLPDDAPMRKYIRFYRDFVLVLNKALAADE
*So CCoAOMT B 149 NYLNYHERLLKLVKLGGLIGYDNTLWNGSVVLPDDAPMRKYIRFYRDFVLVLNKALAADE
Pv CCoAOMT 167 NYVRYHEQLLRLVRVGGTIVYDNTLWAGTVALPPDTPLSDLDRRFSAAIRDLNARLSADD
*Ss CCoAOMT A 140 NYVKYHEQLLRLVRVGESIVYDNTLWAGTVALPPETPMNDLDRRFSAAIRDLNVRLSKDK
*Sh*CCoAOMT2 SCJLRT2050C09.g 168 NYVKYHEQLLRLVRVGGSIVYDNTLWAGTVALPPETPMNDLDRRFSAAIRDLNVRLSKDK
*Sba CCoAOMT A 165 NYVKYHEQLLRLVRVGGSIVYDNTLWAGTVALPPETPMNDLDRRFSAAIRDLNVRLSKDK
*Sr CCoAOMT A 131 NYVKYHEQLLRLVRVGGSIVYDNTLWAGTVALPPETPMNDLDRRFSAAIRDLNVRLSKDE
*So CCoAOMT A 153 NYVKYHEQLLRLVRVGGSIVYDNTLWAGTVALPPETPMNDLDRRFSAAIRDLNVRLSKDE
*Sh*CcoAOMT3 SCJFRZ2010H06.g 172 NYVRYHEQLLRLVRLGGAIVYDNTLWDGTVALPPDAPMSDRDRRFSAAVRDLNARLAADP
Pv CCoAOMT 181 NYVRYHEQLLRLVRVGGAIVYDNTLWDGTVALPPDAPLSDHDRRISAAMRDLNAGLSADE
Pv CCoAOMT 239 RVEICQLPVGDGVTLCRRVK-----
*Sh*CCoAOMT1 SCCCLR1069B09.g 238 RVEICQLPVGDGVTLCRRVKKTC--
*Sba CCoAOMT B 230 RVEVCQLNHIPAAAMAGREHM----
*Ss CCoAOMT B 201 RVEICQLNHI---------------
*Sr CCoAOMT B 208 RVEICQLNHIRGRLQVDHMG-----
*So CCoAOMT B 209 RVEICQLNHIRGRLQVDHMGEL---
Pv CCoAOMT 227 RVEVCQLAIADGVTICRRLV-----
*Ss CCoAOMT A 200 RVEICQLKSLVN-------------
*Sh*CCoAOMT2 SCJLRT2050C09.g 228 RVEVCQLAIADGITICRRLV-----
*Sba CCoAOMT A 225 RVEICQLNHIRGRLQVDHMGELPTR
*Sr CCoAOMT A 191 RVEVCQLNH----------------
*So CCoAOMT A 213 RVEICQLNHIRGRLQVDHMGELPT-
*Sh*CcoAOMT3 SCJFRZ2010H06.g 232 RVEVCQLAVADGVTICRRVV-----
Pv CCoAOMT 241 RVEVCQLTVADGITICRRVV-----

**Supplementary Figure S15-CCR**

Sb CCR 1 MTVVDAVSTDAAGAAPAA--AAAPVVVAQPGNGQTVCVTGAAGYIASWLVKMLLEKGYTV
*Sh*CCR1 SCCCRZ2C01A04.g 1 MTIVDAVSTDAAGAPAA-----AAALVQPAGNGQTVCVTGAAGYIASWLVKLLLEKGYTV
*Sr CCR 1 ------------------------------------------LVIGSWVVKLLLEKGYTV
*Sba CCR 1 -----------------------------------------H--FGSWVVKLLLEKGYTV
*Ss CCR 1 --------------------------------------------FASWVVKLLLEKGYTV
*So CCR 1 --------------------------------------------IASWVVKLLLEKGYTV
*Sh*CCR2 SCCCCL6024F07.g 1 MA-----PPPSVGVLRFAYWVSPL-HLGNSNQQELVCVTGAGGFIGSWVVKELLQRGYRV
Os CCR 1 -------------------MSSNFEANNNNGEKQLVCVTGAGGFIGSWVVKELLIRGYHV


Sb CCR 59 KGTVRNPDDPKNAHLKALDGAAERLILCKADLLDYDAICRAVQGCQGVFHTASPVTDDPE
*Sh*CCR1 SCCCRZ2C01A04.g 56 KGTVRNPDDPKNAHLKALDGAAERLILCKADLLDYDAICRAVQGCHGVFHTASPVTDDPE
*Sr CCR 19 KGTVRNPDDPKNAHLKALDGAAERLILCKADLLDYDAICRAVQGCHGVFHTASPVTDDPE
*Sba CCR 18 KGTVRNPDDPKNAHLKALDGAAERLILCKADLLDYDAICRAVQGCHGVFHTASPVTDDPE
*Ss CCR 17 KGTVRNPDDPKNAHLKALDGAAERLILCKADLLDYDAICRAVQGCHGVFHTASPVTDDPE
*So CCR 17 KGTVRNPDDPKNAHLKALDGAAERLILCKADLLDYDAICRAVQGCHGVFHTASPVTDDPE
*Sh*CCR2 SCCCCL6024F07.g 55 RGTARDPADSKNAHLLALEGAKERLTLCRADVLDRASLHAAFAGCHGVFHVASPVSNDPE
Os CCR 42 RGTARDPADSKNAHLLELEGADQRLSLCRADVLDAASLRAAFSGCHGVFHVASPVSNDPD


Sb CCR 119 QMVEPAVRGTEYVINAAAEAGTVRRVVFTSSIGAVTMDPSRGPDVVVDESCWSDLEFCKK
*Sh*CCR1 SCCCRZ2C01A04.g 116 QMVEPAVRGTEYVINAAAEAGTVRRVVFTSSIGAVTMDPSRGPDVVVDESCWSDLEFCKK
*Sr CCR 79 QMVEPAVRGTEYVINAAAEAGTVRRVVFTSSIGAVTMDPSRGPDVVVDESCWSDLEFCKK
*Sba CCR 78 QMVEPAVRGTEYVINAAAEAGTVRRVVFTSSIGAVTMDPSRGPDVVVDESCWSDLEFCKK
*Ss CCR 77 QMVEPAVRGTEYVINAAAEAGTVRRVVFTSSIGAVTMDPSRGPDVVVDESCWSDLEFCKK
*So CCR 77 QMVEPAVRGTEYVINAAAEAGTVRRVVFTSSIGAVTMDPSRGPDVVVDESCWSDLEFCKK
*Sh*CCR2 SCCCCL6024F07.g 115 L-VPVAVEGTRNVINVAADEG-ARRVVFTSSYGAVHMDPSRSPDAVLDETCWSDYDFCKR
Os CCR 102 L-VPVAVEGTRNVINAAADMG-VRRVVFTSSYGAVHMNPSRSPDAVLDETCWSDYEFCRQ


Sb CCR 179 TRNWYCYGKAVAEQAAWDAARQRGVDLVVVNPVLVVGPLLQPTVNASIAHVLKYLDGSAR
*Sh*CCR1 SCCCRZ2C01A04.g 176 TRNWYCYGKAVAEQAAWDAARQRGVDLVVVNPVLVVGPLLQPTVNASIAHVVKYLDGSAR
*Sr CCR 139 TRNWYCYGKAVAEQAAWDAARQRGVDLVVVNPVLVVGPLLQPTVNASIAHVAKYLDGSAR
*Sba CCR 138 TRNWYCYGKAVAEQAAWDAARQRGVDLVVVNPVLVVGPLLQPTVNASIAHVVKYLDGSAR
*Ss CCR 137 TRNWYCYGKAVAEQAAWDAARQRGVDLVVVNPVLVVGPLLQPTVNASIAHVVKYLDGSAR
*So CCR 137 TRNWYCYGKAVAEQAAWDAARQRGVDLVVVNPVLVVGPLLQPTVNASIAHVVKYLDGSAR
*Sh*CCR2 SCCCCL6024F07.g 173 TDNLYCCAKMMAEITATEEAAARGLQLAVVLPCMTMGPMLQQTLNISNYHVARYVMGTKR
Os CCR 160 TDNLYCCAKMMAEMTATEEAAKRGLELAVVVPSMTMGPMLQQTLNFSSNHVARYLMGTKK


Sb CCR 239 TFANAVQAYVDVRDVADAHLRVFESPAASGRYLCAERVLHREDVVRILAKLFPEYPVPTR
*Sh*CCR1 SCCCRZ2C01A04.g 236 TFANAVQAYVDVRDVADAHLRVFESPRASGRYLCAERVLHREDVVRILAKLFPEYPVPTR
*Sr CCR 199 TFANAVQAYVDVRDVADAHLRVFESPRASGRYLCAERVLHREDVVRILAKLFPEYPVPTR
*Sba CCR 198 TFANAVQAYVDVRDVADAHLRVFESPRASGRYLCAERVLHREDVVRILAKLFPEYPVPTR
*Ss CCR 197 TFANAVQAYVDVRDVADAHLRVFESPRASGRYLCAERVLHREDVVRILAKLFPEYPVPTR
*So CCR 197 TFANAVQAYVDVRDVADAHLRVFESPRASGRYLCAERVLHREDVVRILAKLFPEYPVPTR
*Sh*CCR2 SCCCCL6024F07.g 233 SYPNAVAAYVDVRDVARAHVLVYERPAARGRYLCIGAVLHRAELMAMIRELFPKYPVTAK
Os CCR 220 SYPNAVAAYVDVRDVARAHVLVYERPDARGRYLCIGTVLHRAELLRMLRDLFPQYPATAK


Sb CCR 299 CSDEVNPRKQPYKFSNQKLRDLGLEFRPVSQSLYDTVKNLQEKGHLPVLGEQTTEADKEE
*Sh*CCR1 SCCCRZ2C01A04.g 296 CSDEVNPRKQPYKFSNQKLRDLGLEFRPVSQSLYDTVKNLQEKGHLPVLGEQTTEADDKE
*Sr CCR 259 CSDEVNPRKQPYKFSNQSLVSPTSRTSS--QX----------------------------
*Sba CCR 258 CSDEVNPRKQPYKFSNQSLV----------------------------------------
*Ss CCR 257 CSDEVNPRKQPYKFSNQSLV----------------------------------------
*So CCR 257 CSDEVNPRKQPYKFSNQSLV----------------------------------------
*Sh*CCR2 SCCCCL6024F07.g 293 CEDDGKPMAKPYRFSNQRLRDQGLEFTPLRKSLYETVVCLQHKGHVPVIKQKQRASL---
Os CCR 280 CEDDGKPMAKPYKFSNQRLKDLGLEFTPLRKSLHEAVLCMQQKSHLPLIYPVPKRAYL--


Sb CCR 359 -ANAAAEVQQGGIAIRA
*Sh*CCR1 SCCCRZ2C01A04.g 356 AAPAAAELQQGGIAIRA
*Sr CCR -----------------
*Sba CCR -----------------
*Ss CCR -----------------
*So CCR -----------------
*Sh*CCR2 SCCCCL6024F07.g -----------------
Os CCR -----------------

**Supplementary Figure S16-F5H**

Sb F5H 1 MAAVAKIAMEWLQDPLSCVFLVTLAVVLLQLRRRGKAPLPPGPKPLPIVGNMAMMDQLTH
*Sh*F5H1 SCJLRT1022E04.g 1 MAAVAKIAMEWLQDPLSCVFLVTLAVVLLQLRRRGKAPLPPGPKPLPIVGNMALMDQLTH
*Sba F5H 1 ----------------------------------------------------SLMDQLTH
*So F5H 1 --------------------------------------------PWRPREFDSLMDQLTH
*Ss F5H 1 ------------------------------------------------FTSDSMMDQLTH
*Sr F5H 1 ------------------------------------------------FTSDSLMDQLTH


Sb F5H 61 RGLAALADKYGGLLHLRLGRLHAFAVSTPEYAREVLQTHDGVFSNRPATIAIAYLTYDRA
*Sh*F5H1 SCJLRT1022E04.g 61 RGLAALAEKYGGLLHLRLGRLHAFAVSTPEYAREVLQTQDGVFSNRPATIAIAYLTYDRA
*Sba F5H 9 RGLAALTEKYGGLLHLRLGRLHAFAVSTPEYAREVLQTQDGVFSNRPATIAIAYLTYDRA
*So F5H 17 RGLAALAEKYGGLLHLRLGRLHAFAVSTPEYAREVLQTQDGVFSNRPATIAIAYLTYDRA
*Ss F5H 13 RGLAALAEKYGGLLHLRLGRLHAFAVSTPEYAREVLQTQDGVFSNRPATIAIAYLTYDRA
*Sr F5H 13 RGLAALAEKYGGLLHLRLGRLHAFAVSTPEYAREVLQTQDGVFSNRPATIAIAYLTYDRA


Sb F5H 121 DMAFAHYGPFWRQMRKLCVMKLFSRRRAETWVAVRDECAALVRAVA--TSGGEKAVNLGE
*Sh*F5H1 SCJLRT1022E04.g121 DMAFAHYGPFWRQMRKLCVMKLFSRRRAETWVAVRDECAALVRGVAVSSGGGEKAVNLGE
*Sba F5H 69 DMAFAHYGPFWRQMRKLCVMKLFSRRRAETWVAVRDECAALVRGVAVSSGGGEKAVNLGE
*So F5H 77 DMALAHYGPFWRQMRKLCVMKLFSRRRAETWVAVRDECAALVRGVAVSSGGGEKAVNLGE
*Ss F5H 73 DMAFAHYGPFWRQMRKLCVMKLFSRRRAETWVAVRDECAALVRGVAVSSGGGEKAVNLGE
*Sr F5H 73 DMAFAHYGPFWRQMRKLCVMKLFSRRRAETWVAVRDECAALVRGVAVSSGGGEKAVNLGE

Sb F5H 179 LIFTLTKNVTFRAAFGTRDGEDQEEFIAILQEFSKLFGAFNIGDFLPWLGWMDLQGINRR
*Sh*F5H1 SCJLRT1022E04.g181 LIFNLTKNVTFRAAFGTRDGEDQEEFIAILQEFSKLFGAFNIGDFLPWLGWMDLQGINRR
*Sba F5H 129 LIFNLTKNVTFRAAFGTRDGEDQEEFIAILQEFSKLFGAFNIGDFLPWLGWMDLQGINRR
*So F5H 137 LIFNLTKNVTFRAAFGTRDGEDQEEFIAILQEFSKLFGAFNIGDFLPWLGWMDLQGINRR
*Ss F5H 133 LIFNLTKNVTFRAAFGTRDGEDQEEFIAILQEFSKLFGAFNIGDFLPWLGWMDLQGINRR
*Sr F5H 133 LIFNLTKNVTFRAAFGTRDGEDQEEFIAILQEFSKLFGAFNIGDFLPWLGWMDLQGINRR


Sb F5H 239 LRAARSALDRFIDKIIDEHVKRGKSPDDADADMVDDMLAFFAEAKPAAVNGGAAANGDDL
*Sh*F5H1 SCJLRT1022E04.g241 LRAARSALDRFIDKIIDEHVKRGKSPDDADADMVDDMLAFFAEAK---------------
*Sba F5H 189 LRAARSALDRFIDKIIDEQNHIRGRLQV---DHMGELPTRWM------------------
*So F5H 197 LRAARSALDRFIDKIIDEQITSEFAAA--------CRSTIWESSQRVGCI----------
*Ss F5H 193 LRAARSALDRFIDK----------------------------------------------
*Sr F5H 193 LRAARSALDRFIDKIIDEQIT---------------------------------------


Sb F5H 299 QNTLRLTRDNIKAIIMDVMFGGTETVASAIEWAMAEMMHSPDDLRRVQQELADVVGLDRN
*Sh*F5H1 SCJLRT1022E04.g ------------------------------------------------------------
*Sba F5H ------------------------------------------------------------
*So F5H ------------------------------------------------------------
*Ss F5H ------------------------------------------------------------
*Sr F5H ------------------------------------------------------------


Sb F5H 359 VNESDLDKLPFLKCVIKETLRLHPPIPLLLHETADDCVVGGGGGRRYSVPRGSRVMINVW
*Sh*F5H1 SCJLRT1022E04.g ------------------------------------------------------------
*Sba F5H ------------------------------------------------------------
*So F5H ------------------------------------------------------------
*Ss F5H ------------------------------------------------------------
*Sr F5H ------------------------------------------------------------


Sb F5H 419 AIGRHRGSWKDADVFRPSRFTPDGDAAGLDFKGGCFEFLPFGSGRRSCPGTALGLYALEL
*Sh*F5H1 SCJLRT1022E04.g ------------------------------------------------------------
*Sba F5H ------------------------------------------------------------
*So F5H ------------------------------------------------------------
*Ss F5H ------------------------------------------------------------
*Sr F5H ------------------------------------------------------------


Sb F5H 479 AVAQLAHGFNWSLPDGMKPSELDMSDVFGLTAPRATRLYAVPTPRLNCPLY
*Sh*F5H1 SCJLRT1022E04.g ---------------------------------------------------
*Sba F5H ---------------------------------------------------
*So F5H ---------------------------------------------------
*Ss F5H ---------------------------------------------------
*Sr F5H ---------------------------------------------------

**Supplementary Figure S17- COMT**

Sb COMT 1 MGSTAEDVAAVADEEACMYAMQLASSSILPMTLKNALELGLLEVLQKD--AGKALAAEEV
 *So COMT 1 -------------------------LSILPMTLKNALELGLLEVLQAEAPAGKALAPEEV
 *Sba COMT 1 ------------------------HLSILPMTLKNALELGLLEVLQAEAPAGKALAPEEV
 *Ss COMT 1 ----------------------RIHLSILPMTLKNALELGLLEVLQAEAPAGKALAPEEV
 *Sr COMT 1 ---CIQRVGSSPIWSTC--RRPRIHLSILPMTLKNALELGLLEVLQAEAPAGKALAPEEV
 Ms COMT 1 MGSTAEDVAAVADEEACMYAMQLASSSILPMTLKNALELGLLEVLQAEAPAGKALAPEEV
 *Sh*COMT1 SCJLRT1023B09.g 1 MGSTAEDVAAVADEEACMYAMQLASASILPMTLKNALELGLLEVLQAEAPAGKALAPEEV


 Sb COMT 59 VARLPVAPTNPAAADMVDRMLRLLASYDVVRCQMEDKDGKYERRYSAAPVGKWLTPNEDG
 *So COMT 36 VARLPVAPTNPDAADMVDRMLRLLASYDVVKCQMEDKDGKYERRYSAAPVGKWLTPNEDG
 *Sba COMT 37 VARLPVAPTNPDAADMVDRMLRLLASYDVVKCQMEDKDGKYERRYSAAPVGKWLTPNEDG
 *Ss COMT 39 VARLPVAPTNPDAADMVDRMLRLLASYDVVKCQMEDKDGKYERRYSAAPVGKWLTPNEDG
 *Sr COMT 56 VARLPVAPTNPDAADMVDRMLRLLASYDVVKCQMEDKDGKYERRYSAAPVGKWLTPNEDG
 Ms COMT 61 VARLPVAPTNPGAADMVYRMLRLLASYDVVKCQMEDKDGKYERRYSAAPVGKWLTPNEDG
 *Sh*COMT1 SCJLRT1023B09.g 61 VARLPVAPTNPDAADMVDRMLRLLASYDVVKCQMEDKDGKYERRYSAAPVGKWLTPNEDG


 Sb COMT 119 VSMAALALMNQDKVLMESWYYLKDAVLDGGIPFNKAYGMTAFEYHGTDPRFNRVFNEGMK
 *So COMT 96 VSMAALTLMNQDKVLMESWYYLKDAVLDGGIPFNKAYGMTAFEYHGTDPRFNRVFNEGMK
 *Sba COMT 97 VSMAALTLMNQDKVLMESWYYLKDAVLDGGIPFNKAYGMTAFEYHGTDPRFNRVFNEGMK
 *Ss COMT 99 VSMAALTLMNQDKVLMESWYYLKDAVLDGGIPFNKAYGMTAFEYHGTDPRFNRVFNEGMK
 *Sr COMT 116 VSMAALTLMNQDKVLMESWYYLKDAVLDGGIPFNKAYGMTAFEYHGTDPRFNRVFNEGMK
 Ms COMT 121 VSMAALALMNQDKVLMESWYYLKDAVLDGGIPFNKAYGMTAFEYHGTDPRFNRVFNEGMK
 *Sh*COMT1 SCJLRT1023B09.g121 VSMAALTLMNQDKVLMESWYYLKDAVLDGGIPFNKAYGMTAFEYHGTDPRFNRVFNEGMK


 Sb COMT 179 NHSVIITKKLLEFYTGFDESVSTLVDVGGGIGATLHAITSHHSHIRGINFDLPHVISEAP
 *So COMT 156 NHSVIITKKLLEFYTGFE-GVSTLVDVGGGIGATLHAITSHHPQIKGINFDLPHVISEAP
 *Sba COMT 157 NHSVIITKKLLEFYTGFE-GVSTLVDVGGGIGATLHAITSHHPQIKGINFDLPHVISEAP
 *Ss COMT 159 NHSVIITKKLLEFYTGFE-GVSTLVDVGGGIGATLHAITSHHPQIKGINFDLPHVISEAP
 *Sr COMT 176 NHSVIITKKLLEFYTGFE-GVSTLVDVGGGIGATLHAITSHHPQIKGINFDLPHVISEAP
 Ms COMT 181 NHSVIITKKLLEFYTGFE-GVSTLVDVGGGIGATLHAITSHHPQIRGVNFDLPHVISEAP
 *Sh*COMT1 SCJLRT1023B09.g181 NHSVIITKK---------------------------------------------------

Sb COMT 239 PFPGVQHVGGDMFKSVPAGDAILMKWILHDWSDAHCATLLKNCYDALPEKGGKVIVVECV
 *So COMT 215 PFPGVQHVGGDMFKSVPAGDAILMKWILHDITS---------------------------
 *Sba COMT 216 PFPGVQHVGGDMFKSVPAGDAILMKWILHD------------------------------
 *Ss COMT 218 PFPGVQHVGGDMFKSVPAGDAILMKWI---------------------------------
 *Sr COMT 235 PFPGVQHVGGDMFKSVPAGDAILMKWILHDIT----------------------------
 Ms COMT 240 PFPSVQHVGGDMFKSVPAGDAILMKWILHDWSDAHCATLLKNCYDALPEN-GKVIVVECV
 *Sh*COMT1 SCJLRT1023B09.g ------------------------------------------------------------

Sb COMT 299 LPVTTDAVPKAQGVFHVDMIMLAHNPGGRERYEREFRDLAKAAGFSGFKATYIYANAWAI
 *So COMT ------------------------------------------------------------
 *Sba COMT ------------------------------------------------------------
 *Ss COMT ------------------------------------------------------------
 *Sr COMT ------------------------------------------------------------
 *Ms COMT 299 LPVNTEAVPKAQGVFHVDMIMLAHNPGGRERYEREFRDLAKGAGFSGFKATYIYANAWAI
 *Sh*COMT1 SCJLRT1023B09.g ------------------------------------------------------------

Sb COMT 359 EFIK
 *So COMT ----
 *Sba COMT ----
 *Ss COMT ----
 *Sr COMT ----
 Ms COMT 359 EFIK
 *Sh*COMT1 SCJLRT1023B09.g ----

**Supplementary Figure S18-CAD**

Pv CAD 2 1 ----------------------------------------------------------MG
*So CAD A 1 ------------------------------------------------------------
*Ss CAD A 1 ------------------------------------------------------------
*Sr CAD A 1 ------------------------------------------------------------
*Sba CAD A 1 ------------------------------------------------------------
*Sh*CAD2 SCEPRZ1011A02.g 1 ----------------------------------------------------------MG
Zm CAD 1 ----------------------------------------------------------MG
*Sh*CAD6 SCACHR1038E08.g 1 ------------------------------------------------------------
Sb CAD 1 ---------------------------------------------------------MEV
*Sr CAD C 1 ------------------------------------------------------------
*Sh*CAD7 SCCCLB1001F10.g 1 -------------------------------------------------------MAPVE
Sb CAD 1 MSYHCRALVQGPFLHPPFSPGAAGAPAPSLGVRVGLPSRALRLPRPARASVEKREQQVTM
*Ss CAD B 1 ------------------------------------------------------------
*Sh*CAD8 SCEQLR1029E05.g 1 -----------------------------------------------------------M
*Sr CAD B 1 ------------------------------------------------------------
*Sba CAD B 1 ------------------------------------------------------------
*So CAD B 1 ------------------------------------------------------------


Pv CAD 2 3 SLASERTVVGWAARDATGHLSPYTYTVRKTGPEDVVVKVLYCGICHTDIHQAKNHLGASK
*So CAD A 1 ---------------------------------------IYCGICHTDIHQAKNHLGASK
*Ss CAD A 1 ---------------------------------------IYCGICHTDIHQAKNHLGASK
*Sr CAD A 1 -----------------------------------------CGICHTDIHQAKNHLRASK
*Sba CAD A 1 ---------------------------------------IYCGICHTDIHQAKNHLGASK
*Sh*CAD2 SCEPRZ1011A02.g 3 SLASERKVVGWAARDATGHLAPYTYTLRSTGPEDVVVKVLYCGICHTDIHQAKNHLGASK
Zm CAD 3 SLASERKVVGWAARDATGHLSPYSYTLRNTGPEDVVVKVLYCGICHTDIHQAKNHLGASK
*Sh*CAD6 SCACHR1038E08.g 1 -------------MNESGKVEPFVFKRRENGVDDVTIKVQYCGMCHTDLHFIQNDWGITM
Sb CAD 4 TPNHTQTVAGWAAMNESGKLEPFIFKRRENGVDDVTIKVEYCGMCHTDLHFIQNDWGITM
*Sr CAD C 1 -------------------------------------LVIYCGICHSDLHTIKNEWKNAN
*Sh*CAD7 SCCCLB1001F10.g 6 AEQHPRSALALAAHDASGRLSPIRISRRDTGDDDVAIQILYCGICHSDLHTIKNEWKNAN
Sb CAD 61 EEKAGQAAFGWAARDATGVLSPYNFSRRVPKDDDVTIKVLYCGICHTDLHIIKNDWGNAM
*Ss CAD B 1 ---------------------------------------IYCGICHTDLHIIKNDWGNAM
*Sh*CAD8 SCEQLR1029E05.g 2 EEQGGQAAFGWAARDDTGVLSPYNFSRRVPKDDDVTIKVLYCGICHTDLHIIKNDWGNAM
*Sr CAD B 1 ----------------------------------------YCGICHTDLHIIKNDWGNAM
*Sba CAD B 1 ---------------------------------------IYCGICHTDLHIIKNDWGNAM
*So CAD B 1 ---------------------------------------IYCGICHTDLHIIKNDWGNAM


Pv CAD 2 63 YPMVPRHEVVGEVVEVGPEVSKHRVGDVVGVGVIVGCCRECRPCKANVEQYCNKRIWSYN
*So CAD A 22 YPMVPGHEVVGEVVEVGPEVTKYXVGDVVGVGVIVGXCRECKPCKANVEQYRNKKIWSYN
*Ss CAD A 22 YPMVPGYEVVGEVVEVGPEVTKYGVGDVVGVGVIVGCCRECNPCKANVEQYCNKKIWSYN
*Sr CAD A 20 YPMVPGHEVVGEVVEVGPEVTKYGVGDVVGVGVIVGCCRECNPCKANVEQYCNKKIWSYN
*Sba CAD A 22 YPMVPGHEVVGEVVEVGPEVTKYGVGDVVGVGVIVGCCRECNPCKANVEQYCNKKIWSYN
*Sh*CAD2 SCEPRZ1011A02.g 63 YPMVPGHEVVGEVVEVGPEVTKYGVGDVVGVGVIVGCCRECNPCKANVEQYCNKKIWSYN
Zm CAD 63 YPMVPGHEVVGEVVEVGPEVAKYGVGDVVGVGVIVGCCRECSPCKANVEQYCNKKIWSYN
*Sh*CAD6 SCACHR1038E08.g 48 YPLVPGHEITGVVTRVGSNVSGFRVGDRVGVGCIAASCLDCDHCRRSEENYCDKVTLTYN
Sb CAD 64 YPLVPGHEITGVVTKVGSNVTGFGVGDRVGVGCIAASCLDCDHCRRSEENYCDKVTLTYN
*Sr CAD C 24 YPVVPGHEIAGLITEVGKNVKKFNVGDKVGVGCMVNTCQSCESCEEGHENYCSKIIFTYN
*Sh*CAD7 SCCCLB1001F10.g 66 YPVVPGHEIAGRITEVGKNVKKFNVGDKVGVGCMVNTCQSCESCEEGHENYCSKIIFTYN
Sb CAD 121 YPVVPGHEIVGVVTGVGGGVTRFKAGDTVGVGYFVGSCRSCDSCGKGNENYCAGVVQTSN
*Ss CAD B 22 YPVVPGHEIVGVVTGVGGGVTRFKAGDTVGVGYFVGSCRSCDSCGKGNENYCTGVVQTSN
*Sh*CAD8 SCEQLR1029E05.g 62 YPVVPGHEIVGVVTGVGGGVTRFKAGDTVGVGYFVGSCRSCESCGKGNENYCAGVVQTSN
*Sr CAD B 21 YPVVPGHEIVGVVTGVGGGVTRFKAGDTVGVGYFVGSCRSCESCGKGNENYCAGVVQTSN
*Sba CAD B 22 YPVVPGHEIVGVVTGVGGGVTRFKAGDTVGVGYFVGSCRSCESCGKGNENYCAGVVQTSN
*So CAD B 22 YPVVPGHEIVGVVTGVGGGVTRFKAGDTVGVGYFVGSCRSCESCGKGNENYCAGVVQTSN


Pv CAD 2 123 DVY--TDGRPTQGGFASTMVVDQKFVVPIPAGLAPEQAAPLLCAGVTVYSPLKHFGL-TA
*So CAD A 82 DVY--TDGRPTQGGFASTMVVDQKFVMKIPAGLAPEQAAPLLCAGVTVYSPLKAFGL-TT
*Ss CAD A 82 DVY--TDGRPTQGGFASTMVVDQKFVMKIPAGLAPEQAAPLLCAGVTVYSPLKAFGL-TT
*Sr CAD A 80 DVY--TDGRPTQGGFASTMVVDQKFVMKIPTGLAPEQAAPLLCAGVTVYSPLKAFGL-TT
*Sba CAD A 82 DVY--TDGRPTQGGFASTMVVDQKFVMKIPAGLTPEQAAPLLCAGVTVYSPLKAFGL-TT
*Sh*CAD2 SCEPRZ1011A02.g 123 DVY--TDGRPTQGGFASTMVVDQKFVMKIPAGLAPEQAAPLLCAGVTVYSPLKAFGL-TT
Zm CAD 123 DVY--TDGRPTQGGFASTMVVDQKFVVKIPAGLAPEQAAPLLCAGVTVYSPLKHFGL-TT
*Sh*CAD6 SCACHR1038E08.g 108 GVF--WDGSVTYGGYSRMLVAHKRFVVRVPDTLPLDAAAPLLCAGITVYSPMKHHGMLRS
Sb CAD 124 GVF--PDGSVTYGGYSKMLVAHKRFVVRVPDTLPLDAAAPLLCAGITVYSPMKHHGMLRS
*Sr CAD C 84 SHD--RDGTVTYGGYSDMVVVNERFVIRFPDGMPLDKGAPLLCAGITVYNPMKCHGL-NE
*Sh*CAD7 SCCCLB1001F10.g 126 SHD--RDGTVTYGGYSDMVVVNKRFVIRFPDGMPLDKGAPLLCAGITVYNPMKYHGL-NE
Sb CAD 181 GVDYAHGGVPTKGGFSDVIVVNEHYVVRVPDGLALDRTAPLLCAGVTVYSPMMRHGL-NE
*Ss CAD B 82 GVDQAHGGVPTKGGFSDVIVVNEHYVVRVPDGLALDRTAPLLCAGVTVYSPMMRHGL-NE
*Sh*CAD8 SCEQLR1029E05.g 122 GVDHTHGGVPTKGGFSDVIVVNEHYVVRVPDGLALDRAAPLLCAGVTVYSPMMRHGL-NE
*Sr CAD B 81 GVDNTHGNVPTKGGFSDVIVVNEHYVVRVPDGLALDRTTPLLCAGVTVYSPMMRHGL-NE
*Sba CAD B 82 GVDHTHGGVPTKGGFSDVIVVNEHYVVRVPDGLALDRAAPLLCAGVTVYSPMMRHGL-NE
*So CAD B 82 GVDHTHGGVPTKGGFSDVIVVNEHYVVRVPDGLALDRAAPLLCAGVTVYSPMMRHGL-NE

Pv CAD 2 180 PGLRGGILGLGGVGHMGVKVAKAMGHHVTVISSSSRKRAEAMDELGADAYLVSSDAEAMA
*So CAD A 139 PGLRGAILGLGGVGHMGVKVAKAMGHHVTVISSSSKKRAEAMDHLGADAYLVSSDAAAMA
*Ss CAD A 139 PGLRGAILGLGGVGHMGVKVAKAMGHHVTVISSSSKKRAEAMDHLGADAYLVSSDAAAMA
*Sr CAD A 137 PGLRGAILGLGGVGHMGVKVAKAMGHHVTVISSSSKKRTEAMDHLGADAYLVSSDAAAMA
*Sba CAD A 139 PGLRGAILGLGGVGHMGVKVAKAMGHHVTVISSSSKKRAEAMDHLGADAYLVSSDAAAMA
*Sh*CAD2 SCEPRZ1011A02.g 180 PGLRGAILGLGGVGHMGVKVAKAMGHHVTVISSSSKKRAEAMDHLGADAYLVSSDAAAM-
Zm CAD 180 PGLRGGILGLGGVGHMGVKVAKAMGHHVTVISSSSKKRAEAMDHLGADAYLVSSDAAAMG
*Sh*CAD6 SCACHR1038E08.g 166 PGGSLGVVGLGGLGHVAVKFAKAFGLRVTVISTSPAKEREARERLGADHFVVSTDQKQMQ
Sb CAD 182 AGASLGVVGLGGLGHVAVKFAKAFGLRVTVVSTSPAKEKEARERLGADDFVVSSDQKQMQ
*Sr CAD C 141 PGKHIGVVGLGGLGHVAVKFAKAFGMRVTVISTSPEKREEAMETLGADAFVVSTDANQMK
*Sh*CAD7 SCCCLB1001F10.g 183 PGKHIGVVGLGGLGHVAVKFAKAFGMRVTVISTSPEKREEAMETLGADAFVVSTDANQMK
Sb CAD 240 PGKHLGVVGLGGLGHVAVKFGKAFGMKVTVISTSASKRQEAIESLGADEFLISRDPEEMK
*Ss CAD B 141 AGKHLGVVGLGGLGHVAVKFGKAFGMKVTVISTSASKRQEAIENLGADEFLISRDPEQMK
*Sh*CAD8 SCEQLR1029E05.g 181 PGKHLGVVGLGGLGHVAVKFGKAFGMKVTVISTSASKRQEAIENLGADEFLISRDPEQMK
*Sr CAD B 140 PGKHLGVVGLGGLGHVAVKFGKAFGMKVTVISTSASKRQEAIENLGADEFLISRDPEQMK
*Sba CAD B 141 PGKHLGVVGLGGLGHVAVKFGKAFGMKVTVISTSASKRQEAIENLGADEFLISRDPEQMK
*So CAD B 141 PGKH----------HVAVKFGKAFGMKVTVISTSASKRQEAIENLGADEFLISRDPEQMK


Pv CAD 2 240 AAADSLDYIIDTVPVHHPLEPYLALLRLDGKHVLLGVVGEPLSFVAPMVMLGRKAVTGSF
*So CAD A 199 AAADSLDYIIDTVPVHHPLEPYLALLKLDGKHVLLGVIGEPLSFVSPMVMLGRKAITGSF
*Ss CAD A 199 AAADSLDYIIDTVPVHHPLEPYLALLRLDGKHVLLGVIGEPLSFVSPMVMLGRKAITGSF
*Sr CAD A 197 AAADSLDYIIDTVPVHHPLEPYLALLKLDGKHVLLGVIGEPLSFVSPMVMLGRKAITGSF
*Sba CAD A 199 EAADSLDYIIDTVPVHHPLEPYLALLKLDGKHVLLGVIGEPLGFVSPMVMLGRKAITGSF
*Sh*CAD2 SCEPRZ1011A02.g ------------------------------------------------------------
Zm CAD 240 PAADSLDYIIDTVPVHHPLEPYLALLKLDGKLVLLGVIGEPLSFVSPMVMLGRKAITGSF
*Sh*CAD6 SCACHR1038E08.g 226 AMARSLDYVIDTVSAKHSLGPILELLKVNGKLVLVAAPDQPVELPSFPLIFGKRTVSGSM
Sb CAD 242 AMARSLDYVIDTVSAKHSLGPILELLKVNGKLVLVAAPDQPVELPSFPLIFGKRTVSGSM
*Sr CAD C 201 AVKGTMHGILNTASASMSMYSYLALLKPHGKMILLGLPEKPLQISAFSLVAGGKTLAGSC
*Sh*CAD7 SCCCLB1001F10.g 243 AVKGTMHGILNTASASMSMYSYLALLKPHGKMILLGLPEKPLQISAFSLVAGGKTLAGSC
Sb CAD 300 AATGTMDGIIDTVSAWHPITPLLALLKPLGQMVVVGGPSKPLELPAYAIVPGGKGVAGNS
*Ss CAD B 201 AATGTMDGIIDTVSAWHPITPLLALLKPLGQMVIVGGPSKPLELPVYAFVPGGKGVAGNS
*Sh*CAD8 SCEQLR1029E05.g 241 AATGTMDGIIDTVSAWHPITPLLALLKPLGQMVIVGGPSKPLELPVYAFVPGGKGVAGNS
*Sr CAD B 200 AATGTMDGIIDTVSAWHPITPLLALLKPLGQMVIVGGPSKPLELPAYAFVPGGKGVAGNS
*Sba CAD B 201 AATGTMDGIIDTVSAWHPITPLLALLKPLGQMVIVGGPSKPLELPAYAFVPGGKGVAGNS
*So CAD B 191 AATGTMDGIIDTVSAWHPITPLLALLKPLGQMVIVGGPSKPLELPAYAFVPGGKGVAGNS


Pv CAD 2 300 IGSIDETAELLRFCVDKGLTSQIEVVKMGYVNEALERLERNDVRYRFVVDVAGSNIEEAT
*So CAD A 259 IGSIDETTEVLQFCVDKRLTSQIEVVKMGYVNEALDRLERNDVRYRFVIDIESLV-----
*Ss CAD A 259 IGSIDETAEVLQFCVDKGLTSQIEVVKMGYVNEALDRLERNDVRYXFVIDI---------
*Sr CAD A 257 IGSIDETAEVLQFCVDKGLTSQIEVVKMGYVNEALDRLERNDVRYRFVIDIESL------
*Sba CAD A 259 IGSIDETAEVLQFCVDKGLTSQIEVVKMGYVNEALDRLERNDVRYRFVIDIESL------
*Sh*CAD2 SCEPRZ1011A02.g ------------------------------------------------------------
Zm CAD 300 IGSIDETAEVLQFCVDKGLTSQIEVVKMGYVNEALERLERNDVRYRFVVDVAGSNVEAEA
*Sh*CAD6 SCACHR1038E08.g 286 TGGMKETQEMLDLCGQHNITCDIELVSTDGINEALARLARNDVRYRFVIDIAGDSNSKL-
Sb CAD 302 TGGMKETQEMLDLCGQHGITCDIELVSTDGINDALQRLASNDVRYRFVIDIAGGDSSNSK
*Sr CAD C 261 MGSIRDTQGMMDFAAKHGLTADIELIGPEEVNEAMERLAKGEVRYRFVIDV---------
*Sh*CAD7 SCCCLB1001F10.g 303 MGSIRDTQGMMDFAAKHGLTADIELIGPEEVNEAMERLAKGEVRYRFVIDVGNTLSAASL
Sb CAD 360 VGSVGDCQAMLEFAGKHGIGAEVEVIKMDYVNTAIERLEKNDVRYRFVIDVAGSLGSAA-
*Ss CAD B 261 VGSVGDCQAMLEFAGKHGIGAEVEVIKMDYVNTAIERLEKNDVRYRFVIDI---------
*Sh*CAD8 SCEQLR1029E05.g 301 VGSVGDCQAMLEFAGKHGIGAEVEVIKMDYVNTAIERLEKNDVRYRFVIDVAGSLGSAA-
*Sr CAD B 260 VGSVGDCQAMLEFAGKHGIGAEVEVIKMDYVNTAIERLEKNDVRYRFVIDIESLV-----
*Sba CAD B 261 VGSVGDCQAMLEFAGKHGIGAEVEVIKMDYVNTAIERLEKNDVRYRFVIDIESLV-----
*So CAD B 251 VGSVGDCQAMLEFAGKHGIGAEVEVIKMDYVNTAIERLEKNDVRYRFVIDIESLV-----

Pv CAD 2 360 GAPAN---
*So CAD A --------
*Ss CAD A --------
*Sr CAD A --------
*Sba CAD A --------
*Sh*CAD2 SCEPRZ1011A02.g --------
Zm CAD 360 AAADAASN
*Sh*CAD6SCACHR1038E08.g --------
Sb CAD 362 L-------
*Sr CAD C --------
*Sh*CAD7SCCCLB1001F10.g 363 ASSPVPAL
Sb CAD --------
*Ss CAD B --------
*Sh*CAD8 SCEQLR1029E05.g --------
*Sr CAD B --------
*Sba CAD B --------
*So CAD B --------

**Reference**

1. Bottcher, A. *et al.* Lignification in sugarcane: biochemical characterization, gene discovery, and expression analysis in two genotypes contrasting for lignin content. *Plant Physiol.* **163,** 1539–57 (2013).
